# Tumor-intrinsic PRC2 inactivation drives a context-dependent immune-desert microenvironment and is sensitized by immunogenic viruses

**DOI:** 10.1172/JCI153437

**Published:** 2022-09-01

**Authors:** Juan Yan, Yuedan Chen, Amish J. Patel, Sarah Warda, Cindy J. Lee, Briana G. Nixon, Elissa W.P. Wong, Miguel A. Miranda-Román, Ning Yang, Yi Wang, Mohini R. Pachai, Jessica Sher, Emily Giff, Fanying Tang, Ekta Khurana, Sam Singer, Yang Liu, Phillip M. Galbo, Jesper L.V. Maag, Richard P. Koche, Deyou Zheng, Cristina R. Antonescu, Liang Deng, Ming O. Li, Yu Chen, Ping Chi

**Affiliations:** 1 Human Oncology and Pathogenesis Program, Memorial Sloan Kettering (MSK) Cancer Center, New York, New York, USA.; 2 Weill Cornell Graduate School of Medical Sciences, Cornell University, New York, New York, USA.; 3 Immunology Program, Sloan Kettering Institute,; 4 Louis V. Gerstner Jr. Graduate School of Biomedical Sciences, and; 5 Dermatology Service, Department of Medicine, MSK Cancer Center, New York, New York, USA.; 6 Institute for Computational Biomedicine,; 7 Meyer Cancer Center, and; 8 Department of Physiology and Biophysics, Weill Cornell Medicine, New York, New York, USA.; 9 Department of Surgery, MSK Cancer Center, New York, New York, USA.; 10 Department of Genetics, Albert Einstein College of Medicine, Bronx, New York, USA.; 11 Center for Epigenetics Research, MSK Cancer Center, New York, New York, USA.; 12 Department of Neurology, and; 13 Department of Neuroscience, Albert Einstein College of Medicine, Bronx, New York, USA.; 14 Department of Pathology, MSK Cancer Center, New York, New York, USA.; 15 Weill Cornell Medical College, New York, New York, USA.; 16 Department of Medicine, MSK Cancer Center, New York, New York, USA.

**Keywords:** Oncology, Therapeutics, Antigen presentation, Cancer, Cancer immunotherapy

## Abstract

Immune checkpoint blockade (ICB) has demonstrated clinical success in “inflamed” tumors with substantial T cell infiltrates, but tumors with an immune-desert tumor microenvironment (TME) fail to benefit. The tumor cell–intrinsic molecular mechanisms of the immune-desert phenotype remain poorly understood. Here, we demonstrated that inactivation of the polycomb-repressive complex 2 (PRC2) core components embryonic ectoderm development (*EED*) or suppressor of zeste 12 homolog (*SUZ12*), a prevalent genetic event in malignant peripheral nerve sheath tumors (MPNSTs) and sporadically in other cancers, drove a context-dependent immune-desert TME. PRC2 inactivation reprogramed the chromatin landscape that led to a cell-autonomous shift from primed baseline signaling-dependent cellular responses (e.g., IFN-γ signaling) to PRC2-regulated developmental and cellular differentiation transcriptional programs. Further, PRC2 inactivation led to diminished tumor immune infiltrates through reduced chemokine production and impaired antigen presentation and T cell priming, resulting in primary resistance to ICB. Intratumoral delivery of inactivated modified vaccinia virus Ankara (MVA) enhanced tumor immune infiltrates and sensitized PRC2-loss tumors to ICB. Our results identify molecular mechanisms of PRC2 inactivation–mediated, context-dependent epigenetic reprogramming that underline the immune-desert phenotype in cancer. Our studies also point to intratumoral delivery of immunogenic viruses as an initial therapeutic strategy to modulate the immune-desert TME and capitalize on the clinical benefit of ICB.

## Graphical abstract

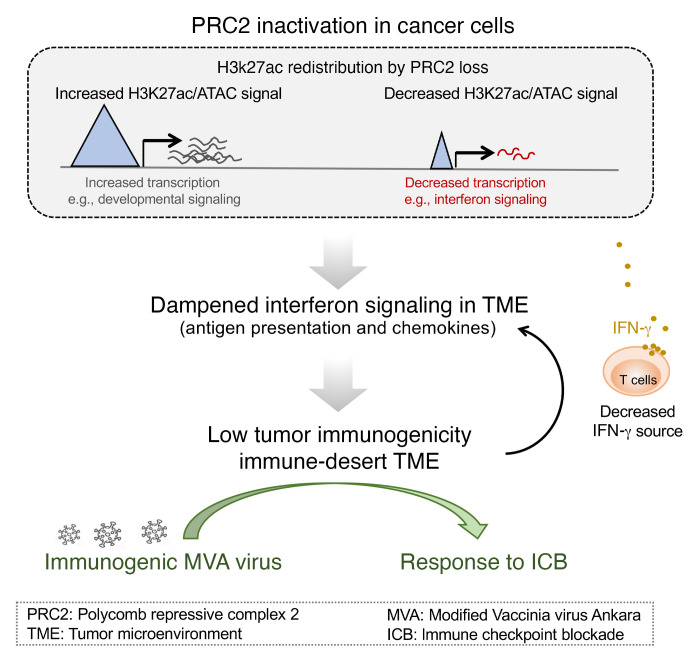

## Introduction

Polycomb-repressive complex 2 (PRC2), consisting of core components of enhancer of zeste homolog 1/-2 (EZH1/2), embryonic ectoderm development (EED), and suppressor of zeste 12 homolog (SUZ12), establishes and maintains H3K27me2/3 in the genome and regulates chromatin structure, transcription, cellular stemness, and differentiation (1). PRC2 is a context-dependent tumor suppressor, whose core components are frequently inactivated genetically or epigenetically in various cancer types, including malignant peripheral nerve sheath tumors (MPNSTs) (2, 3), melanoma (2), myeloid disorders (4, 5), T cell acute lymphocytic leukemia (ALL) (6), early T cell precursor ALL (7), pediatric gliomas (8–11), invasive breast cancer (12), and other cancers. Among all cancer types, high-grade MPNST, a group of aggressive soft tissue sarcomas with no effective therapies, has the highest prevalence of complete loss of PRC2 function through biallelic inactivation of the PRC2 core components *EED* or *SUZ12* (2, 3, 13–15).

There is increasing evidence demonstrating the critical context-dependent role of PRC2 in regulating immune cell identity and function, including cytotoxic CD8^+^ T cell repression (16–21), CD4^+^ T helper cell repression (22–24), and Treg activation (25–27) through regulation of cell lineage–specific gene expression. Therefore, selectively targeting PRC2 in immune cells may modulate the tumor microenvironment (TME) and tumor responses to immunotherapy (28). Beyond immune cells, PRC2 in cancer cells has been shown to maintain bivalency at MHC class I (MHC-I) antigen–processing genes and silence MHC-I expression in selective MHC-I^lo^ cancers (e.g., small cell lung cancer, neuroblastoma); targeting PRC2 can potentially enhance antitumor immunity by increasing MHC-I antigen presentation in this setting (29). However, it remains unclear how tumor-intrinsic PRC2 inactivation affects the tumor immune microenvironment and whether PRC2 has similar regulation of MHC-I in MHC-I^hi^ cancers.

Here, using both human MPNST tissues and engineered PRC2-loss murine models, we demonstrated that tumor-intrinsic PRC2 loss promoted immune evasion and an immune-desert TME through epigenetic reprogramming and consequent deficiency in antigen presentation, chemokine production, and IFN-γ signaling, as well as primary resistance to immune checkpoint blockade (ICB). Further, we demonstrated that intratumoral delivery of the inactivated immunogenic modified vaccinia virus Ankara (MVA) enhanced tumor immunity, altered the immune-desert TME, and sensitized the PRC2-loss tumors to ICB therapy. These studies indicate that genetic inactivation of PRC2 can be used as a biomarker for resistance to ICB therapy in selective cancers and that engineered immunogenic viruses can be used to enhance tumor immunity and serve as an initial immunomodulatory strategy to overcome the cold TME in PRC2-loss tumors.

## Results

### Tumor-intrinsic PRC2 loss is associated with an immune-desert TME in MPNST and other cancers.

**Figure 1:**
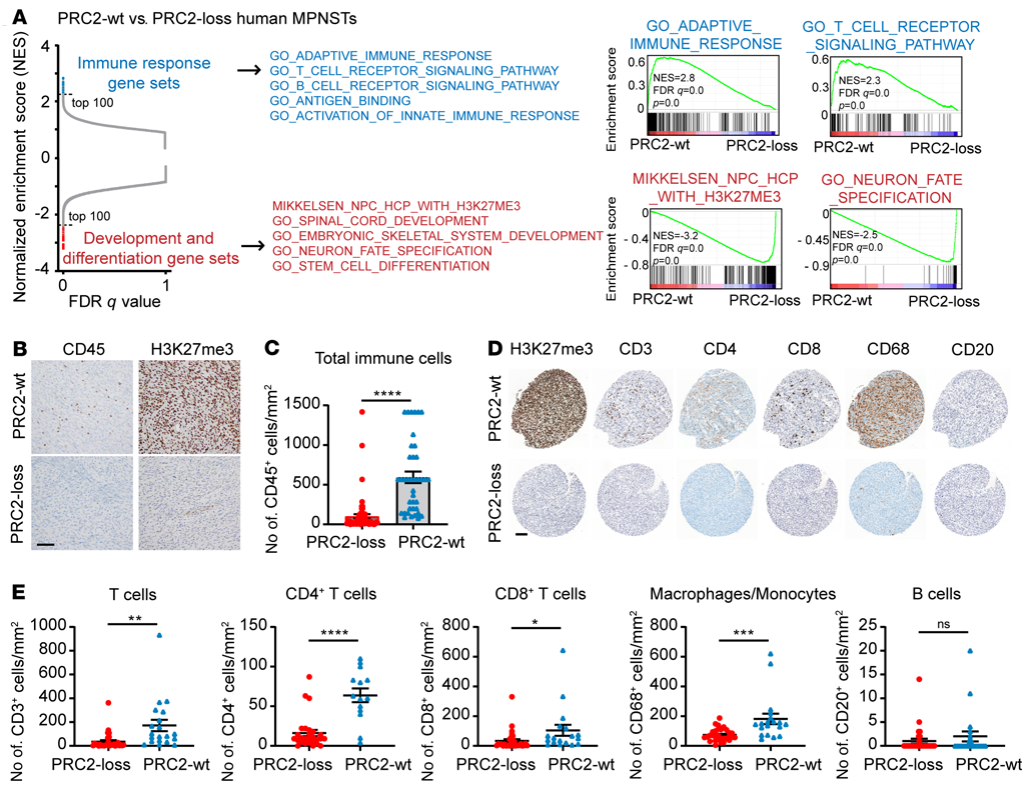
PRC2 loss is associated with a deficiency of broad subclasses of tumor immune infiltrates in MPNSTs. (**A**) Differentially enriched gene sets by GSEA of RNA-Seq transcriptomes of PRC2-wt (*n* = 15) and PRC2-loss (*n* = 26) human MPNSTs. Dashed lines indicate the top 100 ranked gene sets. (**B** and **C**) Representative IHC (**B**) and quantification (**C**) of H3K27me3 and CD45 in PRC2-loss (*n* = 69) and PRC2-wt (*n* = 39) MPNSTs. Scale bar: 100 μm. (**D** and **E**) IHC of representative samples (**D**) and quantification (**E**) of H3K27me3 and immune cell markers in PRC2-loss (*n* >27) and PRC2-wt (*n* >14) MPNST TMAs. Scale bar: 100 μm. *P < 0.05, **P < 0.01, ***P < 0.001, and ****P < 0.0001, by unpaired, 2-tailed *t* test (**C** and **E**). Data indicate the mean ± SEM.

To characterize the role of PRC2 inactivation in cancer pathogenesis, we analyzed the transcriptomes of 41 histologically confirmed high-grade human MPNST tumor samples, consisting of both wild-type PRC2 (referred to hereafter as PCR2-wt) and PRC2-loss samples. PRC2 loss in MPNSTs was confirmed by the loss of H3K27me3 immunostaining and/or genetic inactivation of *EED* or *SUZ12* by MSK-IMPACT (MSK – integrated mutation profiling of actionable cancer targets) (30) (Supplemental Figure 1A and Supplemental Table 1; supplemental material available online with this article; https://doi.org/10.1172/JCI153437DS1). Principal component analysis (PCA), which detects sources of variation, showed that the MPNST samples were readily separated by PRC2 status in the first principal component (PC1) (Supplemental Figure 1B). We generated a gene set composed of genes that were differentially expressed between PRC2-loss and PRC2-wt samples. Hierarchical clustering based on these genes robustly separated the PRC2-loss and PRC2-wt MPNSTs, with the majority of the most differentially expressed genes upregulated in PRC2-loss compared with PRC2-wt MPNSTs, consistent with the role of PRC2 in transcriptional repression (Supplemental Figure 1C). Consistently, gene set enrichment analysis (GSEA) showed that the most enriched pathways and gene sets in PRC2-loss MPNSTs included the PRC2 modules and H3K27me3 target genes, organ development and morphogenesis, neuron cell fate specification, and WNT signaling gene sets (3, 31–33) (Figure 1A, Supplemental Figure 1D, and Supplemental Table 2). A distinct smaller subset of genes was consistently downregulated in PRC2-loss compared with PRC2-wt MPNSTs. Remarkably, nearly all of these gene sets were associated with immune function, including both innate and adaptive immune response pathway genes, T and B cell receptor signaling pathway genes, and antigen-binding and presentation genes (Figure 1A, Supplemental Figure 1C, and Supplemental Table 2).

We next performed IHC immunostaining for several established markers of distinct immune subclasses in human MPNST tumor tissue, including CD45 (pan-leukocyte), CD3 (T cells, CD4^+^ and CD8^+^ subsets), CD68 (macrophages/monocytes), and CD20 (B cells). Quantification of each immune subset revealed that, compared with PRC2-wt, the PRC2-loss MPNSTs were associated with significant reductions in CD45^+^ leukocytes (Figure 1, B and C), CD3^+^, CD4^+^, and CD8^+^ T cells, and CD68^+^ macrophages/monocytes (Figure 1, D and E). Although not significantly different, CD20^+^ B cells were only marginally present in both PRC2-wt and PRC2-loss MPNSTs (Figure 1, D and E). These data corroborate the transcriptome results of diminished tumor immune infiltrates (Supplemental Figure 1E). To further corroborate that PRC2 inactivation in various cancer contexts was associated with a cold TME, we identified available archival tumor tissues with confirmed PRC2 inactivation through *EED* or *SUZ12* loss-of-function mutations by MSK-IMPACT and loss of H3K27me3 immunostaining by IHC (Supplemental Table 3 and Supplemental Figure 1F). IHC of CD45^+^ leukocytes demonstrated that these PRC2-loss tumors had low levels of tumor immune infiltrates that were comparable to those of PRC2-loss MPNSTs (Supplemental Figure 1F and Figure 1C). These results suggest that tumor cell–intrinsic PRC2 inactivation may exclude immune infiltrates and drive an immune-desert TME in various cancer contexts.

### Antigen presentation and IFN-γ signaling are suppressed in PRC2-loss MPNSTs.

**Figure 2:**
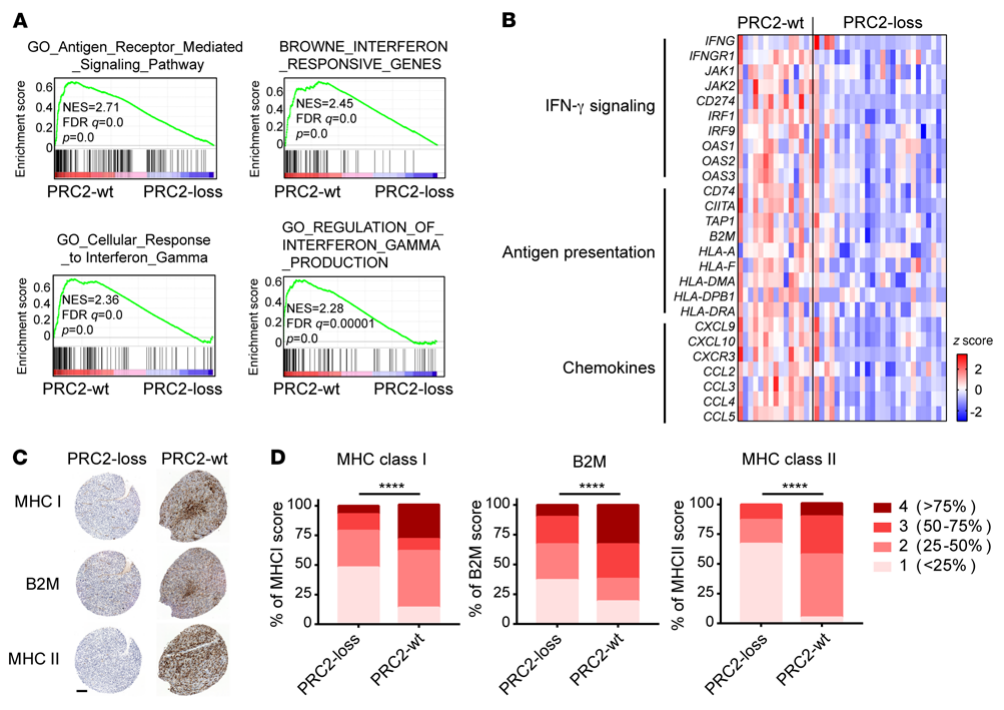
Antigen presentation and IFN-γ pathway genes are diminished in PRC2-loss compared with PRC2-wt MPNSTs. (**A**) GSEA of human MPNST transcriptomes pointed to antigen presentation and IFN-γ pathway defects in PRC2-loss compared with PRC2-wt tumors. (**B**) Heatmap of IFN-γ signaling and downstream gene expression in PRC2-wt (*n* = 15) and PRC2-loss (*n* = 26) human MPNSTs. (**C** and **D**) IHC of representative samples (**C**) and quantification (**D**) of MHC-I, MHC-II, and B2M in PRC2-loss (*n* >27) and PRC2-wt (*n* >14) human MPNST TMAs. Scale bar: 100 μm. ****P < 0.0001, by χ^2^ test for contingency in **D**.

To understand how tumor-intrinsic PRC2 loss leads to an immune-desert TME, we further characterized the transcriptomes of PRC2-loss versus PRC2-wt MPNSTs and focused on the initiating steps of the antitumor immune response (34). Among the genes downregulated by PRC2-loss compared with PRC2-wt tumors, antigen processing and presentation was one of the most negatively enriched gene sets by GSEA (Figure 2A and Supplemental Table 2). IFN-γ is an established key regulator of antigen presentation and chemokine for immune cell recruitment (35, 36). Consistently, IFN-γ signaling was impaired in PRC2-loss tumors, including cellular responses to IFN-γ, IFN-responsive genes, and regulation of IFN-γ production (Figure 2A). Expression levels of the tumor cell–autonomous gene *IFNGR1* and downstream signaling axis and effector genes, including *JAK1*, *JAK2*, and *IRF1*, were all significantly lower in PRC2-loss tumors than in PRC2-wt tumors (*P* < 0.05; Figure 2B). In addition, the expression of *IFNG* encoding IFN-γ, which is usually produced by immune cells, was markedly low in most PRC2-loss tumors (Figure 2B), consistent with the PRC2-loss–associated immune-desert phenotype. Overall, expression of the *IFNG*/*IFNGR1*/*JAK* signaling axis genes was markedly reduced in PRC2-loss compared with PRC2-wt tumors. Consequently, accompanying the PRC2 loss, the expression of genes relevant to antigen processing and presentation, including MHC-I (e.g., *HLA-A* and *B2M*), MHC-II (e.g., *CD74* and *HLA-DMA*), and antigen processing (e.g., *TAP1*) (Figure 2B), was also decreased, suggesting decreased tumor immunogenicity in PRC2-loss compared with PRC2-wt tumors. We further validated the decreased protein expression of MHC-I, B2M, and MHC-II by IHC in PRC2-loss tumors using an MPNST tissue microarray (TMA) (Figure 2, C and D). Furthermore, the key chemokines responsible for immune cell recruitment, including *CXCL9/10* (*P* < 0.01) and *CCL2/3/4/5* (*P* < 0.05), were significantly lower in PRC2-loss tumors than in PRC2-wt tumors (Figure 2B). These data further posit that the tumor cell–intrinsic PRC2-loss–associated immune-desert phenotype is driven by impaired antigen presentation and diminished IFN-γ signaling in tumors.

### PRC2 loss reprograms the chromatin landscape and suppresses a subset of IFN-responsive genes.

**Figure 3:**
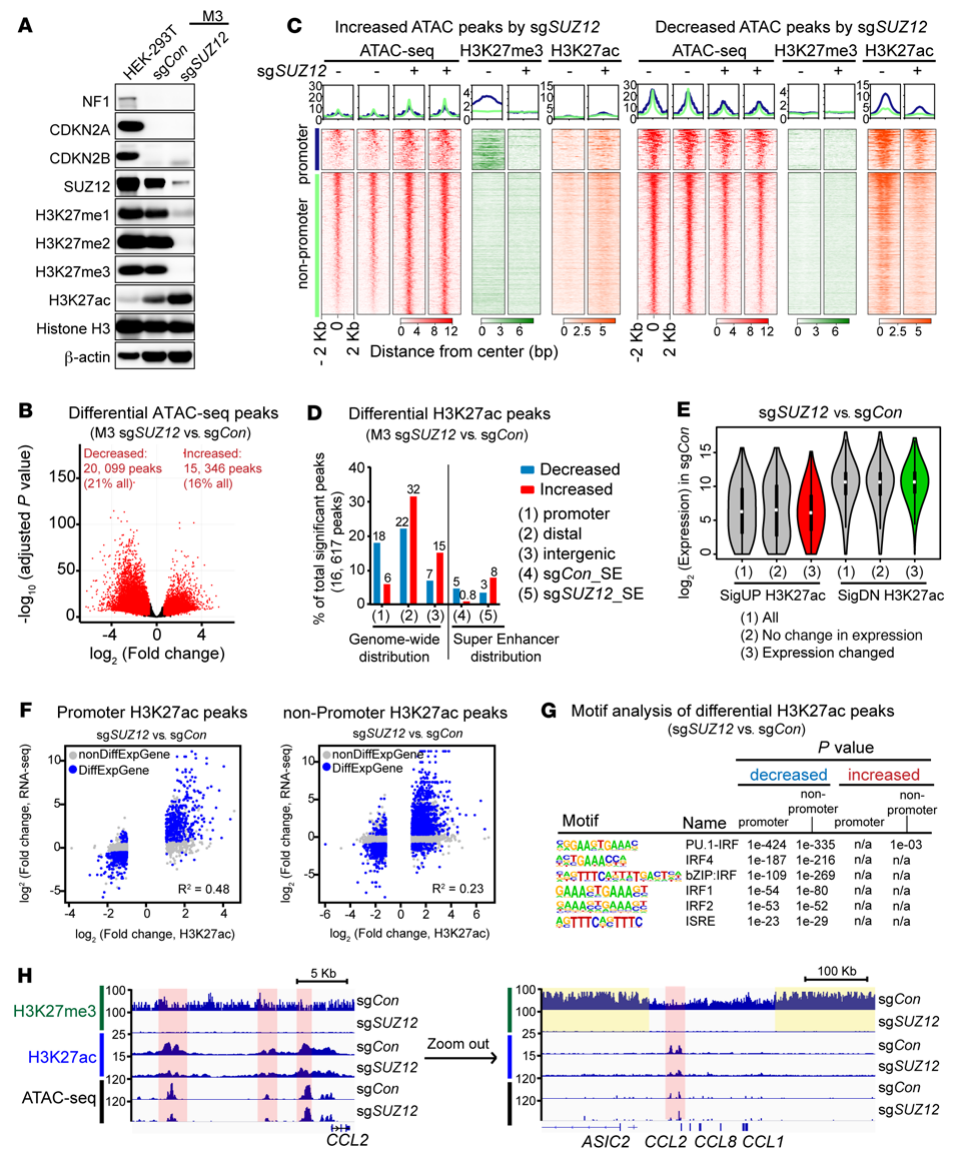
PRC2 loss reprograms the chromatin landscape and suppresses a subset of IFN-γ–responsive genes. (**A**) Immunoblots of the indicated proteins in PRC2-isogenic human MPNST cells. (**B**) Volcano plot of chromatin accessibility changes by ATAC-Seq. Red dots represent markedly changed ATAC peaks (FDR *q* < 0.1, fold change ≥1.5). (**C**) Density plot of histone modifications by ChIP-Seq, centered on markedly increased (left) and decreased (right) ATAC peaks by *SUZ12* knockout in M3 cells. Promoter: transcriptional start site (TSS) ± 2 kb; nonpromoter: rest of the genomic regions other than promoters, including distal regulatory enhancers and intergenic regions. (**D**) Distribution of differential H3K27ac peaks across different genomic regions in PRC2-isogenic M3 cells (FDR *q* ˂ 0.05, fold change ≥2), including promoter (TSS ± 2 kb), distal regulatory (–50 kb from the TSS to the transcriptional end site [TES] + 5 kb), and intergenic (nonpromoter, nondistal regulatory) regions and SEs. (**E**) Violin plots of mRNA baseline expression of genes associated with PRC2 loss induced significantly increased (SigUP) and decreased (SigDN) H3K27ac at their respective loci in M3 sg*Con* cells. (**F**) Correlation of transcriptome and H3K27ac enrichment changes at promoter (TSS ± 2 kb) and nonpromoter (FDR *q* < 0.05, fold change ≥2) regions. Blue and gray dots represent peaks mapped to genes with (DiffExpGene) and without (nonDiffExpGene) significant transcriptome changes, respectively. (**G**) HOMER motif analysis of PRC2-loss–associated significantly decreased and increased H3K27ac peaks in PRC2-isogenic M3 cells. e, exponents of 10. (**H**) ChIP-Seq and ATAC-Seq profiles at the loci of representative IFN-γ–responsive genes, e.g., *CCL2*, in PRC2-isogenic M3 cells. Pink indicates an H3K27ac change; yellow indicates an H3K27me3 change.

To examine the role of PRC2 loss in MPNST while minimizing cell line–specific confounding factors, we generated and validated PRC2-isogenic human MPNST cells using CRISPR/Cas9-mediated knockout of the PRC2 core component *SUZ12* in a PRC2-wt, *NF1^–/–^ CDKN2A^–/–^* M3 cell line derived from a human NF1–associated MPNST (Figure 3A). *SUZ12* loss led to a global reduction of the H3K27me3, H3K27me2, and H3K27me1 marks and a reciprocal global increase in the H3K27ac mark in PRC2-isogenic human MPNST cells (sg*Con* vs. sg*SUZ12*) (Figure 3A). The PRC2-isogenic M3 cells, when orthotopically transplanted into the sciatic nerve pockets of immunodeficient NSG mice, gave rise to high-grade MPNSTs with histological (e.g., monotonous spindle cell morphology, herringbone pattern, and fascicular growth) and immunostaining (e.g., H3K27me3 and Ki67 staining) features resembling those of high-grade human MPNST (13) (Supplemental Figure 2A). Nucleoplasmic and chromatin fractionation demonstrated that the global decrease in H3K27me3 and the increase in H3K27ac occurred on chromatin in *SUZ12*-loss tumor cells (Supplemental Figure 2B). These characterizations combined with the loss of H3K27me3 immunostaining in PRC2-loss M3 cell line–derived MPNST tumors validated the model system for mechanistic studies.

We speculated that PRC2 loss may alter the chromatin context and directly affect the transcriptional regulation of genes related to immune signaling and responses. We first examined the impact of PRC2 loss on genome-wide distribution of chromatin accessibility by the assay for transposase-accessible chromatin using sequencing (ATAC-Seq) and PRC2-relevant chromatin marks by ChIP-Seq and CUT&RUN in PRC2-isogenic M3 cells. Globally, compared with PRC2-wt, loss of PRC2 not only led to a marked increase but also a marked decrease in chromatin accessibility at 15,346 (16% of all ATAC peaks) and 20,099 (21% of all ATAC peaks) genomic loci, respectively (Figure 3B). The substantially changed chromatin accessibility regions (increased or decreased) were relatively similarly distributed at promoter (7.7%, 8.3%) and nonpromoter regions, including distal regulatory (59.9%, 68.8%) and intergenic regions (32.4%, 22.9%) (Supplemental Figure 2C). The markedly increased ATAC peaks by PRC2 loss overlapped more with H3K27me3-enriched loci (10.5%) compared to the decreased ATAC peaks (2.4%) (Supplemental Figure 2D), consistent with the PRC2 function in chromatin compaction and transcription repression. Importantly, the integration of differential ATAC peaks with multiple histone modifications showed that the increased/decreased H3K27ac peaks were well correlated with open/closed chromatin accessibility changes at both promoter and nonpromoter regions as a result of PRC2 loss (Figure 3C and Supplemental Figure 2, E and F). The global distribution of H3K36me2 and H3K36me3 chromatin marks that had been previously described to interact with PRC2 and H3K27me3 (37–42) were not obviously affected, especially surrounding the H3K27me3-enriched regions in the PRC2-wt context (Supplemental Figure 2, F–H).

H3K27ac is an established chromatin mark preferentially enriched at active promoters and distal regulatory enhancers (13, 43, 44). Despite the increase in the total amount of chromatin-bound H3K27ac with PRC2 loss, we found relatively balanced gains and losses in the genome when regions enriched with H3K27ac were compared (Figure 3D). While the markedly increased H3K27ac peaks mainly localized to distal regulatory and intergenic regions and overlapped with H3K27me3 peaks, the markedly decreased H3K27ac peaks mainly localized to promoters and existing superenhancers (SEs) in PRC2-wt control tumor cells, with minimal overlap with H3K27me3 (Figure 3D and Supplemental Figure 2I). Consistently, the increased H3K27ac peaks in the PRC2/H3K27me3 loss context showed a trend toward closer distances to the nearest H3K27me3 peaks compared with the decreased H3K27ac peaks, while their distances to H3K36me3 peaks were reversed (Supplemental Figure 2J). Interestingly, we observed that with PRC2/H3K27me3 loss, a diffuse H3K27ac signal spread into the H3K27me3-enriched regions in the PRC2-wt context, without enrichment of specific genomic loci, leading to a mild overall increase in the baseline H3K27ac signal (e.g., loci 1–4) (Supplemental Figure 2, G and H). Notably, the decreased H3K27ac peaks localized to genes with higher baseline expression levels compared with increased H3K27ac peaks (Figure 3E). Moreover, H3K27ac changes were well correlated with transcriptome changes at both promoter and nonpromoter regions (Figure 3F). Motif analysis revealed significant enrichment of transcription factor binding motifs associated with immune signaling pathways and responses (e.g., the IFN signaling–associated IRF family and ISRE motifs) only in the decreased, but not the increased, H3K27ac peaks associated with PRC2 loss (Figure 3G and Supplemental Table 4). In addition, we observed that a subset of IFN-γ–responsive gene loci were directly affected by PRC2 loss. For example, H3K27ac enrichment at the SE locus of the monocyte chemotactic protein *CCL2* was significantly (*P* > 0.05, fold change > 2) diminished by PRC2 loss (sg*SUZ12*) compared with controls (Figure 3H), whereas H3K27ac enrichment at other IFN-γ target gene loci (e.g., *IRF1*, *CD274*) was unchanged (Supplemental Figure 2K). We observed that many decreased and increased SE regions by PRC2 loss were preferentially flanked by broad enrichment of H3K27me3 in the genome of PRC2-wt tumor cells (Figure 3H and Supplemental Figure 2L). Further, inhibition of the main histone acetyl transferase (CBP/P300) for H3K27ac by A-485, or of the binding of bromodomain (BRD) proteins to H3K27ac by JQ1, suppressed IFN-γ–responsive *CCL2* gene expression in PRC2-wt M3 cells (Supplemental Figure 2M). These data indicated that *CCL2* expression in the PRC2-wt context required H3K27ac modification at the *CCL2* locus and that the diminished expression of *CCL2* in the PRC2-loss context was a direct consequence of the decreased H3K27ac enrichment at the *CCL2* locus. These data suggest that PRC2 loss affects the global distribution of H3K27ac and reprograms the genome-wide chromatin context of both the promoter and enhancer landscapes, which in turn alters signaling-dependent transcriptional responses (Supplemental Figure 2N).

### Decreased chromatin accessibility for IFN-γ–responsive gene loci in PRC2-loss MPNST cells.

**Figure 4:**
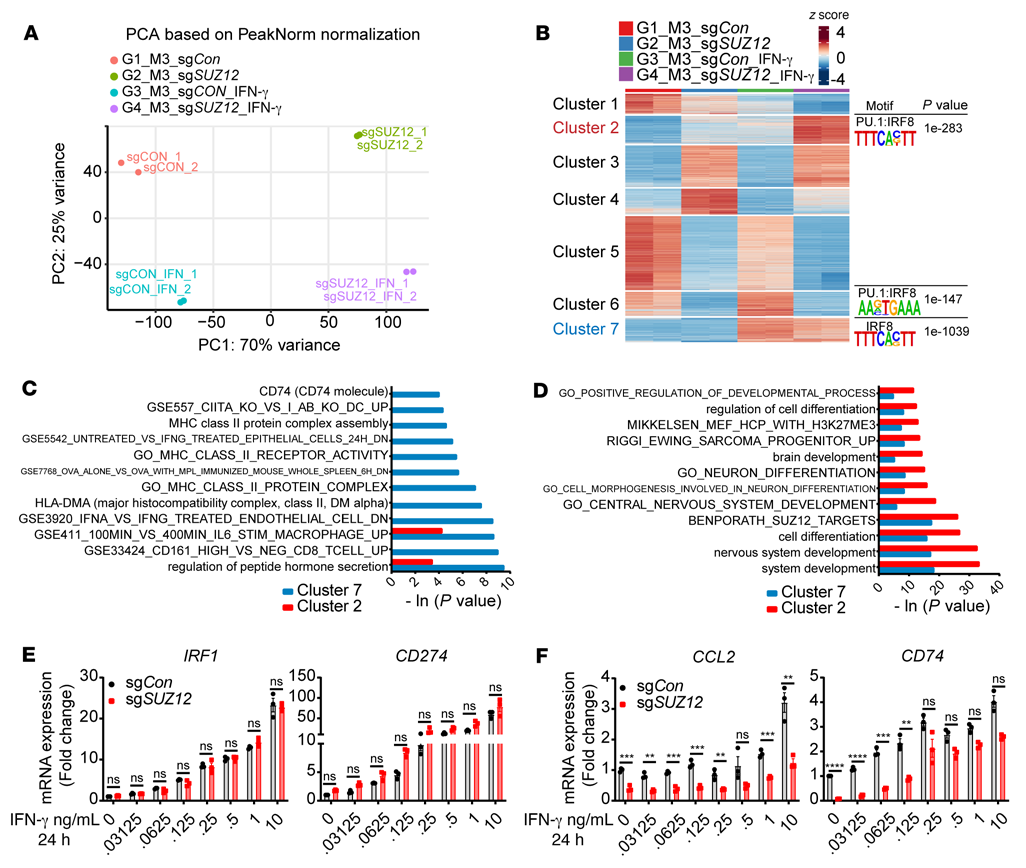
PRC2 loss dampens the IFN-γ response in tumor cells through decreased chromatin accessibility. (**A**) PCA of chromatin accessibility by ATAC-Seq in PRC2-wt (sg*Con*) and PRC2-loss (sg*SUZ12*) human MPNST cells with or without IFN-γ stimulation. (**B**) *K*-means clustering analysis of the chromatin accessibility changes in PRC2-isogenic M3 cells with or without IFN-γ stimulation. Cells were treated with or without 10 ng/mL IFN-γ for 24 hours followed by ATAC-Seq. Enriched IRF motifs by HOMER de novo motif analysis in clusters 2, 6, and 7. (**C** and **D**) Comparison of significantly enriched pathways between cluster 7 (**C**) and cluster 2 (**D**) by GO analysis. (**E** and **F**) IFN-γ dose–dependent mRNA expression changes of IFN-γ–responsive genes by qRT-PCR without (**E**) or with (**F**) PRC2-loss–associated chromatin accessibility changes (*n* = 3). Results were normalized to sg*Con* without the stimulation condition. **P < 0.01, ***P < 0.001, and ****P < 0.0001, by multiple unpaired *t* tests (**E** and **F**). (*P* < 0.05, FDR *q* < 0.05 and a fold change ≥2 was significant). Data indicate the mean ± SEM.

One of the fundamental functions of the chromatin context is to prime the cells for the signaling-dependent transcriptional response. Reasoning that an altered local chromatin context mediated by PRC2 loss may change the transcriptional output, we next examined the transcriptome changes of IFN-γ–responsive genes in response to IFN-γ stimulation in PRC2-isogenic MPNST cells. Stimulation with 10 ng/mL exogenous IFN-γ for 24 hours had no significant impact on the levels of H3K27me3 modification or *SUZ12*/*EED* mRNA expression in PRC2-isogenic M3 cells (Supplemental Figure 3, A and B). PCA of ATAC-Seq replicates under various conditions demonstrated robust clustering of replicates and separation of samples based on PRC2 status (PC1) and IFN-γ stimulation (PC2) (Figure 4A). *K*-means clusters 2, 6, and 7 were most representative of increased chromatin accessibility in response to IFN-γ stimulation in the PRC2-loss (sg*SUZ12*) and PRC2-wt (sg*Con*) contexts, respectively (Figure 4B). Consistently, de novo motif analysis of the *K*-means clusters showed that only clusters 2, 6, and 7 identified IFN-γ stimulation–related motifs (e.g., IRF8 and PU.1:IRF8) but with differential significance. The IFN-γ–related motif IRF8 was the top-most enriched motif of cluster 7, with a *P* value of 1 × 10^–1039^, consistent with a primed chromatin context in response to IFN-γ stimulation through IRF activation in the PRC2-wt context. In contrast, the top-most enriched motif for cluster 2 was the GCN4/AP1 motif (*P* = 1 × 10^–1223^), whereas the classic IFN-γ signaling–relevant motif IRF8/PU.1 was less significantly enriched (*P* = 1 × 10^–283^) (Figure 4B and Supplemental Table 5), suggesting a shift from IRF signaling to GCN4/AP1 signaling in a PRC2-loss–primed chromatin context. Moreover, gene ontology (GO) analysis also revealed substantial differences between the PRC2-loss representative cluster 2 and the PRC2-wt representative cluster 7 of chromatin accessibility changes induced by IFN-γ. GO analysis revealed that cluster 7 was most enriched for immune response–related signaling pathways, including pathways for antigen presentation and IFN signaling (Figure 4C), whereas cluster 2 was most enriched for development-related pathways, e.g., cellular differentiation, organ development, and PRC2 and H3K27me3 target pathways (Figure 4D).

Nevertheless, the transcriptome change tracked changes in the local chromatin context. In response to IFN-γ stimulation, the transcription of IFN-γ response genes (e.g., *IRF1* and *CD274*) was unchanged when the local chromatin context was unperturbed (Figure 4E and Supplemental Figure 2K), and the transcriptional activation of IFN-γ response genes (e.g., *CCL2*, *CD74*, *CIITA*, and *HLA-DRA*) was significantly blunted (Figure 4F and Supplemental Figure 3C) when the gene loci were associated with a decrease in chromatin accessibility and H3K27ac signals as a result of PRC2 loss (Figure 3H and Supplemental Figure 2E). Further, the blunted transcriptional activation of these genes was most pronounced when the IFN-γ source was low (Figure 4F and Supplemental Figure 3C). We also restored EED in an *EED*-mutant human MPNST cell line sNF96.2 and validated the restoration of PRC2 function by H3K27me3 using a doxycycline-inducible *EED* system (Supplemental Figure 3, D and E). Reexpression of wild-type EED (PRC2 restoration) in sNF96.2 cells enhanced the expression of IFN-γ–responsive genes such as *CCL2* and *CD74* that were otherwise downregulated as a result of PRC2 loss (Supplemental Figure 3F). These observations suggest that PRC2 loss reprograms the steady-state primed chromatin context and leads to a blunted IFN-γ response in tumor cells.

### Engineered PRC2 loss recapitulates the diminished IFN-γ signaling and the cold TME in both MPNST and breast cancer murine models.

**Figure 5:**
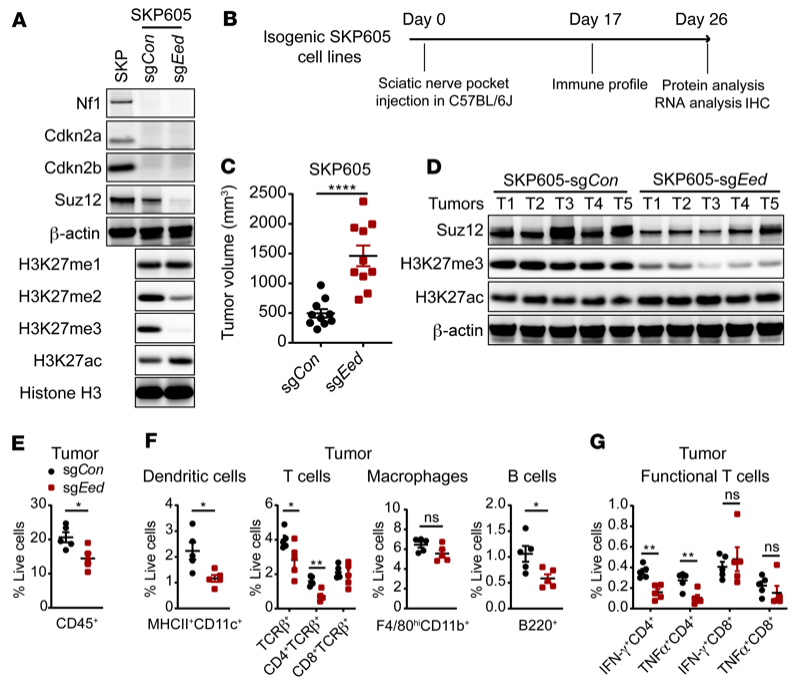
PRC2 loss suppresses tumor immune infiltration in a murine MPNST syngeneic transplantation model. (**A**) Immunoblots of the indicated proteins in PRC2-loss (sg*Eed*) and PRC2-wt (sg*Con*) isogenic murine MPNST SKP605 cells. (**B**) Schematic of the experimental plan and analysis of the orthotopic and syngeneic transplantable murine MPNST model (SKP605). (**C**) Tumor volumes of PRC2-isogenic (sg*Eed* vs. sg*Con*) murine SKP605 tumors explanted on day 26 after sciatic nerve pocket implantation (*n* = 10 tumors bilaterally grafted into C57BL/6J mice for each cohort). (**D**) Immunoblots of the indicated proteins from the PRC2-wt and PRC2-loss murine MPNSTs in **C**. (**E** and **F**) Percentages of total CD45^+^ immune cells (**E**) and subpopulations of immune cells (**F**) among total live cells in PRC2-isogenic murine SKP605 tumors (*n* = 5 tumors per cohort). (**G**) Percentage of IFN-γ^+^CD4^+^ or IFN-γ^+^CD8^+^ T cells among total live cells in PRC2-isogenic SKP605 tumors (*n* = 5 tumors per cohort). *P < 0.05, **P < 0.01, and ****P < 0.0001, by unpaired, 2-tailed *t* test (**C** and **E**–**G)**. Data indicate the mean ± SEM.

To evaluate the impact of tumor cell–intrinsic PRC2 inactivation on the TME in vivo, we generated a histologically confirmed *Nf1^–/–^ Cdkn2a/b^–/–^* murine MPNST tumor–derived cell line (SKP605) from skin-derived precursors (SKPs) of C57BL/6J mice (45, 46) (Supplemental Figure 4, A and B). Using CRISPR/Cas9-mediated knockout of the PRC2 core component *Eed*, we generated and validated PRC2-isogenic murine MPNST cells (SKP605, sg*Con* vs. sg*Eed*) amenable for orthotopic and syngeneic transplantation into immunocompetent C57BL/6J mice (Figure 5, A and B, and Supplemental Figure 4C). The orthotopically transplanted PRC2-loss (sg*Eed*) tumors exhibited accelerated growth in the sciatic nerve pockets of C57BL/6J mice compared with PRC2-wt (sg*Con*) tumors (Figure 5, C and D, and Supplemental Figure 4D). The expression levels of immune cell recruitment chemokines, e.g., *Ccl2* and *Cxcl10*, and the lymphocyte activation cytokine *Il2* were significantly decreased in PRC2-loss compared with PRC2-wt tumors (Supplemental Figure 4E), indicating decreased immune cell infiltration. Profiling of tumor immune infiltrates by FACS demonstrated a significant reduction in CD45^+^ leukocytes in PRC2-loss tumors compared with PRC2-wt tumors (Figure 5E and Supplemental Figure 4F). Further, we observed a reduction of tumor immune infiltrates in PRC2-loss tumors across all major subclasses of immune cells, including MHCII^+^CD11c^+^ DCs, TCRβ^+^ T cells, B220^+^ B cells, and, to a lesser extent, F4/80^hi^CD11b^+^ macrophages (Figure 5F, Supplemental Figure 4G, and Supplemental Figure 5), phenocopying the cold TME of human PRC2-loss MPNSTs. Importantly, the IFN-γ^+^ and TNF-α^+^ CD4^+^ T cells were both significantly reduced in PRC2-loss tumors (Figure 5G and Supplemental Figure 4H). The immune infiltration differences between PRC2-isogenic SKP605 tumors were further confirmed by IHC of CD45^+^ leukocytes and various subclasses of immune cells (Supplemental Figure 4I). These data suggest that PRC2 inactivation in MPNSTs led to diminished recruitment of tumor immune infiltrates and a reduction of functional T cells, which together contributed to a cold TME.

**Figure 6:**
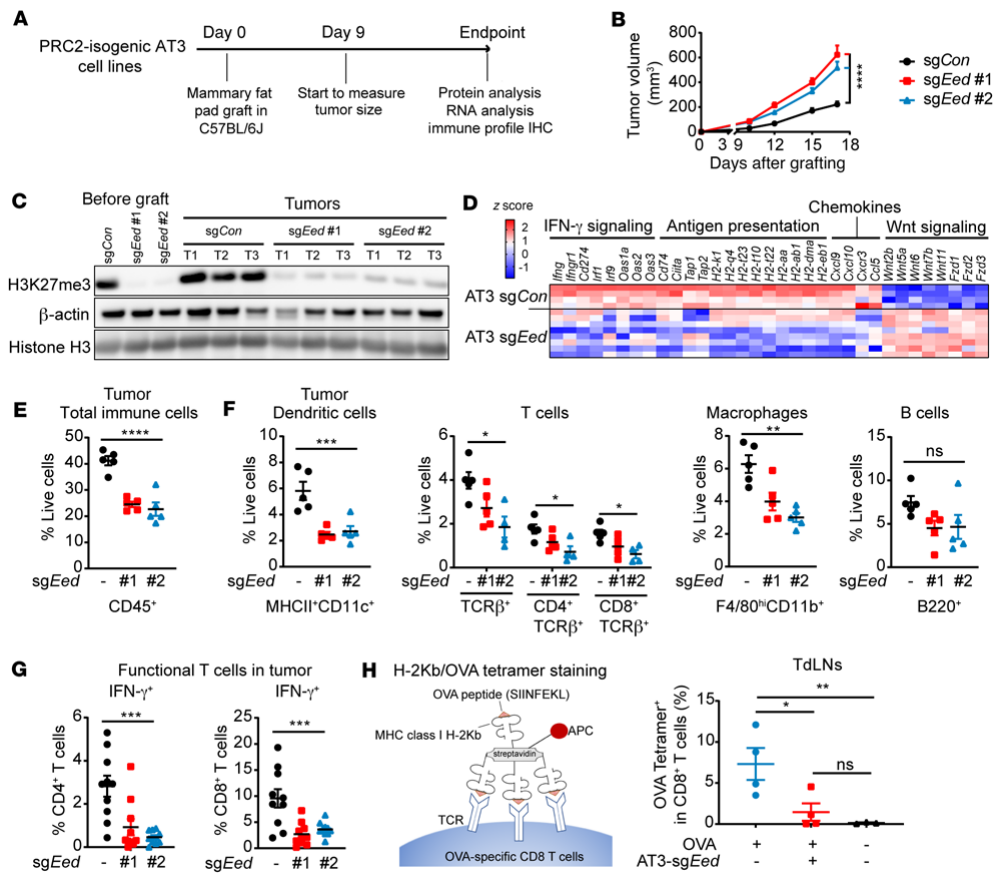
PRC2 loss leads to an antigen presentation defect and recapitulates the immune evasion phenotype in a murine mammary tumor syngeneic transplantation model. (**A**) Schematic of the in vivo experimental and analysis plan for murine AT3 mammary tumors. (**B**) Growth curves of PRC2-isogeneic (sg*Con* vs. sg*Eed*) AT3 syngeneic transplant tumors over time (*n* = 9–10 tumors per cohort). (**C**) Immunoblots of H3K27me3 in pre-graft AT3 cells and explanted AT3 tumors. (**D**) Heatmap of expression changes of genes related to antigen presentation, IFN-γ signaling responses, chemokines, and WNT signaling from RNA-Seq of AT3 tumors. (**E** and **F**) Percentage of total CD45^+^ immune cells (**E**) and subpopulations (**F**) of total live cells in PRC2-isogenic AT3 syngeneic grafted tumors (*n* = 5 tumors per cohort). (**G**) Percentage of IFN-γ^+^ cells among total CD4^+^ and CD8^+^ T cells in PRC2-isogenic AT3 tumors (*n* = 10 tumors per cohort). (**H**) Tetramer staining of OVA-specific CD8^+^ T cells in TdLNs 14 days after transplanting PRC2-isogenic AT3 cells into C57BL/6J mice. TdLNs for OVA^+^ tumors: *n* = 4; TdLNs for OVA^–^ sg*Con* tumors: *n* = 3 pooled from 2 independent experiments. *P < 0.05, **P < 0.01, ***P < 0.001, and ****P < 0.0001, by 1-way ANOVA (**B** and **E**–**G**), with correction for multiple comparisons by 2-stage linear step-up method of Benjamini, Krieger, and Yekutieli (**H**) (FDR *q* < 0.05 was significant). Data indicate the mean ± SEM.

To evaluate whether PRC2 loss has a similar effect on the TME in other cancer types, we used CRISPR/Cas9-mediated knockout of the PRC2 core components *Eed* or *Suz12* and generated a PRC2-isogenic murine mammary tumor model (AT3, sg*Con* vs. sg*Eed* or sg*Suz12*) amenable for syngeneic transplantation into C57BL/6J mice (Supplemental Figure 6A). Although PRC2 loss did not affect tumor cell growth in vitro (Supplemental Figure 6B), it accelerated orthotopically and syngeneically grafted tumor growth in vivo (Figure 6, A and B, and Supplemental Figure 6C). PRC2 and H3K27me3 loss were maintained in grafted PRC2-loss tumors (Figure 6C and Supplemental Figure 6D). Transcriptome analysis of the explanted PRC2-wt (sg*Con*) and PRC2-loss (sg*Eed*) AT3 tumors by RNA-Seq demonstrated that PRC2 loss led to the upregulation of various developmental pathways, including Wnt/β-catenin signaling and PRC2/H3K27me3 targets, and to downregulation of both innate and adaptive immune response pathways, including antigen processing and presentation and IFN-γ response pathways (Supplemental Figure 6E and Supplemental Table 6). Consistently, we observed a significant (*P* < 0.05) reduction in the expression of *Ifng* and IFN-γ signaling–related genes (e.g., *Ifngr1*, *Cd274*, *Irf1*, *Irf9*), antigen presentation–related genes (e.g., *Tap1*, *Tap2*), and MHC-I (e.g., *H2-k1*, *H2-q4*, *H2-23*) and MHC-II (e.g., *H2-t10* and *H2-aa*) genes in PRC2-loss versus PRC2-wt tumors (Figure 6D), indicating impaired tumor immunogenicity by tumor cell–intrinsic PRC2 inactivation. The immune recruitment chemokines were also markedly decreased in PRC2-loss compared with PRC2-wt AT3 tumors (e.g., *Cxcl9*, *Cxcl10, Ccl5*) (Figure 6D). Therefore, engineered PRC2-loss murine mammary tumors phenocopied the transcriptome changes and recapitulated the impaired immunogenicity of PRC2-loss human MPNSTs, irrespective of tumor lineage.

We next analyzed the population change of infiltrating immune cells in explanted PRC2-isogenic syngeneically transplanted AT3 tumors (Supplemental Figure 5). We observed a significant reduction of CD45^+^ leukocytes in PRC2-loss compared with PRC2-wt tumors, which was confirmed by IHC (Figure 6E and Supplemental Figure 6, F and G). Similarly, the reduction of tumor immune infiltrates with PRC2 loss was seen across all major subclasses of immune cells, including MHC-II^+^CD11c^+^ DCs, TCRβ^+^ T cells (both CD4^+^ and CD8^+^), F4/80^hi^CD11b^+^ macrophages, and to a lesser extent B220^+^ B cells (Figure 6F and Supplemental Figure 6H). Importantly, we detected a significant reduction in functional IFN-γ^+^ T cells in both in CD4^+^ and CD8^+^ T cell subclasses (Figure 6G and Supplemental Figure 6I), as well as a reduction of TNFα^+^ and a trend toward a reduction of the granzyme B^+^ (GzmB^+^) CD8^+^ T cells in PRC2-loss tumors (Supplemental Figure 6, J and K). These observations indicate that PRC2 loss in tumors not only led to diminished numbers of T cells, but also significant functional impairment of T cells. Since functional T cells are the main source of IFN-γ, these data indicate that IFN-γ is probably diminished in PRC2-loss tumors, which can further amplify the immune evasion phenotype.

To further dissect how PRC2 loss drives an immune evasion phenotype, we specifically evaluated T cell priming in tumor draining lymph nodes (TdLNs), a critical initial step in the development of antitumor immunity (34, 47). We induced exogenous expression of the model antigen OVA and generated PRC2-isogenic AT3 OVA^+^ cells (sg*Con* vs. sg*Eed*), orthotopically transplanted these cells into the mammary fat pads of C57BL/6J mice, and analyzed the OVA-specific CD8^+^ T cells in TdLNs (Supplemental Figure 6, L and M). The MHC-I OVA tetramer^+^ CD8^+^ T cells were significantly diminished in the TdLNs from mice bearing PRC2-loss OVA^+^ compared with PRC2-wt OVA^+^ AT3 tumors (Figure 6H and Supplemental Figure 6N). These data indicate that PRC2 loss in tumors suppressed the initial antigen cross-presentation by DCs and impaired tumor-specific CD8^+^ T cell priming. These events, combined with the decreased tumor-infiltrating DCs and macrophages as well as diminished expression of immune recruitment chemokines, collectively contributed to the immune-desert TME. The observations from the AT3 murine mammary tumor model also indicated that the PRC2-loss–mediated immune evasion was not restricted to the MPNST context but could be generalized to other cancer types as well.

### Engineered PRC2-loss tumors confer primary resistance to ICB.

**Figure 7:**
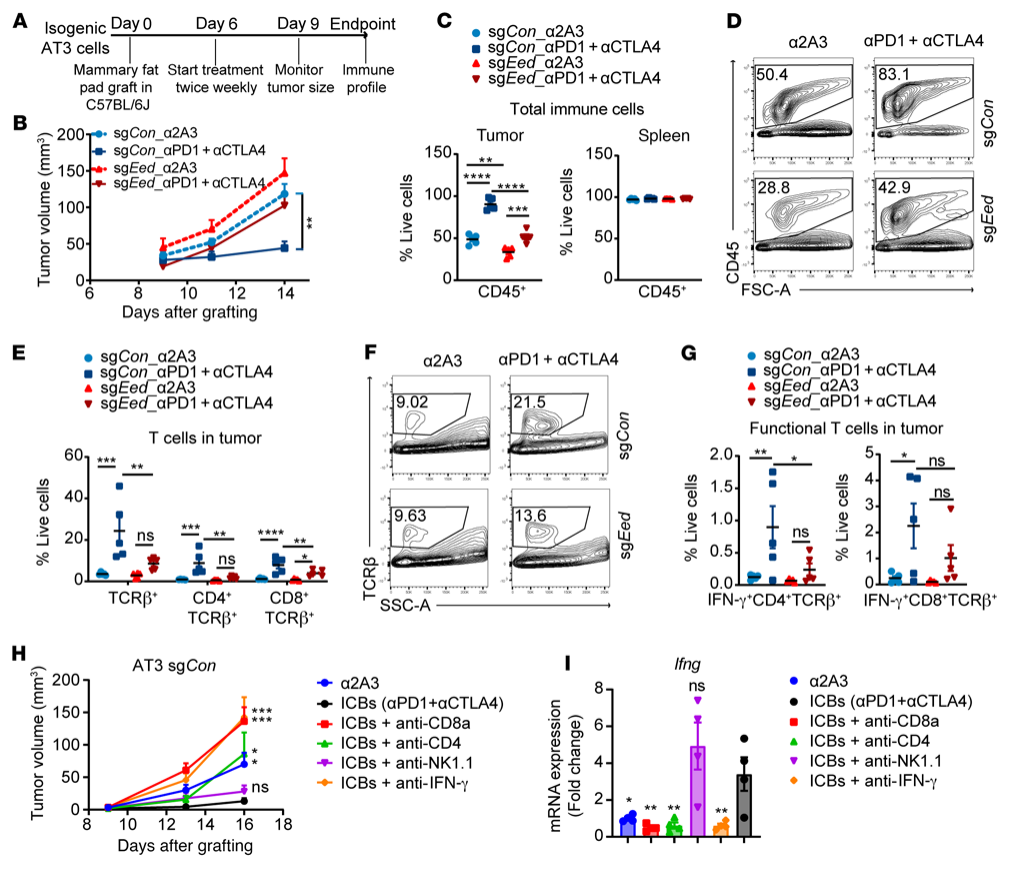
PRC2-loss tumors confer primary resistance to ICB therapies. (**A**) Schematic of the plan for treatment of PRC2-loss AT3 tumors with ICB therapy. (**B**) Tumor growth curves of PRC2-isogenic AT3 tumors with ICB and control treatments over time (*n* = 10 tumors per cohort). (**C**) Percentage of CD45^+^ tumor immune cells among all live cells in PRC2-isogneic AT3 tumors and spleens (*n* = 5 per cohort). (**D**) Representative examples of CD45^+^ cells (gated CD45^+^ cells among all live cells) by FACS for **C**. (**E**) Percentage of T cells in PRC2-isogenic AT3 tumors treated with ICB and controls (*n* = 5 per cohort). (**F**) Representative examples of T cells (gated TCRβ^+^ in CD45^+^ cells) by FACS for **E**. (**G**) Percentage of IFN-γ^+^, CD4^+^, and CD8^+^ T cells among all live cells in tumors (*n* = 5 per cohort). (**H**) Growth curves of AT3 sg*Con* tumors with the indicated treatment over time (*n* = 6–10 tumors per cohort). (**I**) Relative mRNA *Ifng* expression changes by qRT-PCR in tumors from **H**, normalized to the anti-2A3 (α2A3) control treatment condition (*n* = 4 tumors per cohort). *P < 0.05, **P < 0.01, ***P < 0.001, and ****P < 0.0001, by 1-way ANOVA corrected for multiple comparisons by the 2-stage, linear step-up method of Benjamini, Krieger, and Yekutieli (FDR *q* < 0.05 was significant) (**B**, **C**, **E**, and **G**–**I**) compared with the ICB treatment group. Data indicate the mean ± SEM.

Since tumor cell–intrinsic PRC2 loss drives an immune-desert TME, we speculated that PRC2 loss might confer primary resistance to FDA-approved ICB immunotherapies, including anti–programmed cell death 1 (anti–PD-1) and anti–cytotoxic T lymphocyte–associated protein 4 (anti-CTLA4) antibodies (48). We evaluated the therapeutic efficacy of combining anti–PD-1 and anti-CTLA4 antibodies in transplanted PRC2-isogenic AT3 tumor models (Figure 7A). Combined ICB was effective and significantly inhibited the growth of PRC2-wt AT3 tumors; in contrast, it failed to retard the growth of PRC2-loss AT3 tumors (Figure 7B). Moreover, the combined ICB treatment increased CD45^+^ immune infiltrates (Figure 7, C and D, and Supplemental Figure 7A), particularly TCRβ^+^CD4^+^ and TCRβ^+^CD8^+^ T cell infiltrates in the PRC2-wt tumors (Figure 7, E and F, and Supplemental Figure 7, B and C). However, in response to combined ICB treatment, the recruitment of CD45^+^ immune cells, including both CD4^+^ and CD8^+^ T cells, was significantly blunted in the PRC2-loss tumors; this was accompanied by a reduction of functional IFN-γ^+^CD4^+^ and IFN-γ^+^CD8^+^ T cells (Figure 7, E–G). These results demonstrated that PRC2-loss tumors were resistant to ICB therapy.

To evaluate the cellular and molecular components that mediate the ICB treatment responses in PRC2-wt AT3 (sg*Con*) tumors, we used depletion antibodies to selectively deplete CD4^+^ T cells, CD8^+^ T cells, or NK cells and used an IFN-γ–blocking antibody to inhibit IFN-γ signaling. We then examined the ICB treatment responses under these perturbations (Supplemental Figure 7D). We observed that NK cell depletion did not significantly affect the ICB treatment response and that CD4^+^ T cell depletion abolished the ICB treatment response. In contrast, IFN-γ and CD8^+^ T cell depletion not only diminished the ICB treatment responses, but also led to accelerated tumor growth compared with untreated controls (Figure 7H). Consistently, expression levels of *Ifng* and its response genes (e.g., *Cd274*, *Irf1, Ccl4*) were diminished by depletion of IFN-γ^+^, CD4^+^, or CD8^+^ T cells compared with expression levels in the ICB treatment controls (Figure 7I and Supplemental Figure 7E). These data indicate that IFN-γ in the TME was critical for immune surveillance and control of tumor growth as well as for mediating the ICB treatment response, and that the source of IFN-γ was mainly CD8^+^ T cells and partially CD4^+^ T cells.

### Intratumoral delivery of immunostimulatory heat-inactivated MVA sensitizes PRC2-loss tumors to ICB therapy.

**Figure 8:**
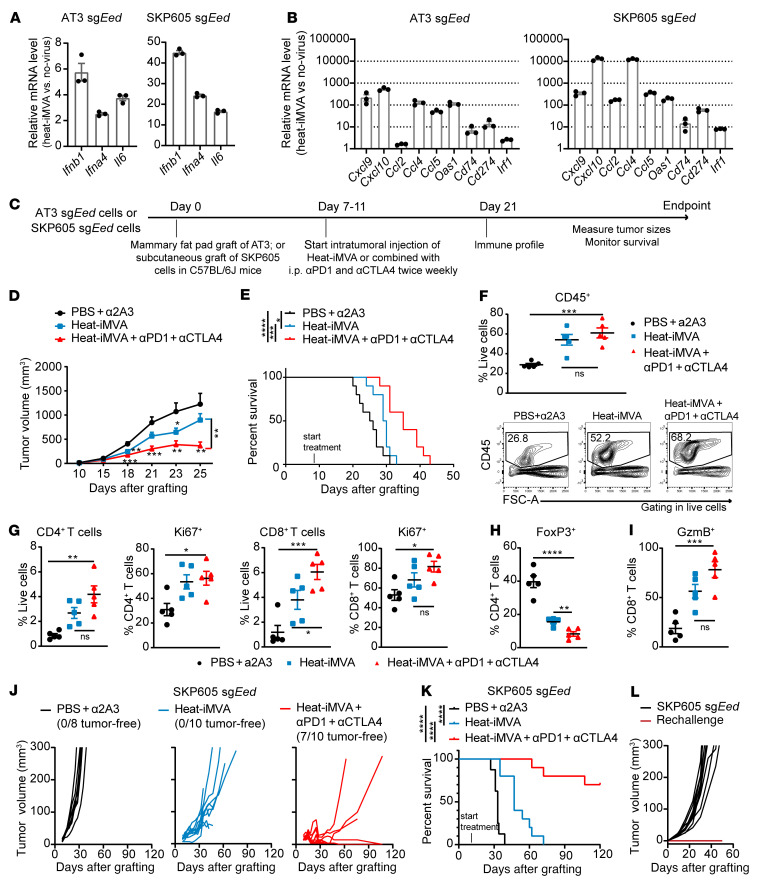
Intratumoral injection of heat-iMVA sensitizes PRC2-loss tumors to anti–PD-1 and anti-CTLA4 therapies in murine cancer models. (**A** and **B**) Fold changes in mRNA expression levels of type I IFN (**A**) and IFN-γ–responsive (**B**) genes by qRT-PCR, 24 hours after heat-iMVA infection in vitro (*n* = 3). (**C**) Schematic of the treatment plan for heat-iMVA and ICB therapy in PRC2-loss AT3 (sg*Eed*) or SKP605 (sg*Eed*) tumors. (**D** and **E**) Tumor growth curves (**D**) and Kaplan-Meier survival curves (**E**) over time in mice with AT3 sg*Eed* tumors under the indicated treatment conditions (*n* = 10 per cohort). (**F**) Percentages and representative FACS profiles of tumor-infiltrating CD45^+^ immune cells in AT3 (sg*Eed*) tumors under the indicated treatment conditions (*n* = 5 per cohort). (**G**) Percentages of CD4^+^ and CD8^+^ T cells among all live cells and the relative Ki67^+^ subpopulation in AT3 (sg*Eed*) tumors under the indicated treatment conditions. (**H** and **I**) Percentages of FoxP3^+^ Tregs among CD4^+^ T cells (**H**) and GzmB^+^ cells among CD8^+^ T cells (**I**) in AT3 (sg*Eed*) tumors under the indicated treatment conditions (*n* = 5 per cohort). (**J** and **K**) Tumor growth curves for each tumor (**J**) and Kaplan-Meier survival curves (**K**) over time for mice with SKP605 (sg*Eed*) tumors under the indicated treatment conditions. SKP605 sg*Eed* cells were s.c. grafted onto the right flank of C57BL/6J mice (*n* = 8–10 tumors per cohort). (**L**) Tumor growth curves for each tumor s.c. grafted onto the left flank of C57BL/6J mice (*n* = 7–10 tumors per cohort). *P < 0.05, **P < 0.01, ***P < 0.001, and ****P < 0.0001, by unpaired, 2-tailed *t* test (**D** and **G**–**I**) between 2 groups; by log-rank (Mantel-Cox) test (**E** and **K)**; and by 1-way ANOVA (**G**–**I**) among 3 groups. Data indicate the mean ± SEM.

Immunogenic modified MVA infection of DCs can induce type I IFN by activating the cyclic GMP-AMP synthase (cGAS) and stimulator of IFN gene–mediated (STING-mediated) cytosolic DNA–sensing pathways (49). Intratumoral (i.t.) delivery of heat-inactivated MVA (heat-iMVA) can generate local and systemic antitumor immunity mediated by CD8^+^ T cells and Batf3-dependent CD103^+^CD8^+^ DCs (50). Thus, we tested whether i.t. delivery of heat-iMVA could enhance the IFN response and innate immunity, modulate the immune-desert TME, and sensitize PRC2-loss tumors to ICB. We observed that heat-iMVA in PRC2-loss murine tumor cells (AT3 [sg*Eed*], SKP605 [sg*Eed*]) not only triggered type I IFN production (e.g., *Ifnb1* and *Ifna4*) (Figure 8A), but also rescued the PRC2-loss–mediated IFN-γ signaling deficiency, resulting in increased chemokines and *Cd274* (*Pdl1*) expression (Figure 8B).

Intratumoral heat-iMVA alone in syngeneically transplanted PRC2-loss AT3 (sg*Eed)* tumors only mildly retarded the tumor growth; however, i.t. heat-iMVA, when combined with anti–PD-1 and anti-CTLA4 ICB treatment, significantly reduced tumor growth (Figure 8, C and D, and Supplemental Figure 8A). This was accompanied by significant prolongation of survival of mice in the heat-iMVA and ICB combination treatment group compared with mice that received ICB alone or vehicle treatment (Figure 8E). Consistently, we observed significantly more cell death in virus-treated PRC2-loss tumors (Supplemental Figure 8B), suggesting an enhanced antitumor effect. Consistently, i.t. heat-iMVA significantly increased CD45^+^ immune cell infiltration in PRC2-loss AT3 (sg*Eed*) tumors compared with vehicle treatment, which was further augmented when combined with ICB treatment (Figure 8F), including TCRβ^+^ T cells and MHC-II^+^CD11c^+^ DCs (Supplemental Figure 8C). We did not observe significant changes in F4/80^hi^CD11b^+^ macrophages or B220^+^ B cells in heat-iMVA–injected tumors (Supplemental Figure 8C). We observed significant enrichment of CD4^+^ and CD8^+^ T cells accompanied by increased proliferation (Ki67^+^) in the heat-iMVA treatment group, with and without ICB treatment, compared with the vehicle-treated tumors (Figure 8G). Moreover, heat-iMVA treatment led to a significant decrease in immune-suppressive FoxP3^+^ Tregs (Figure 8H and Supplemental Figure 8D) as well as a significant increase in GzmB^+^CD8^+^ cytotoxic T cells compared with vehicle-treated tumors (Figure 8I and Supplemental Figure 8E), especially when combined with ICB therapy.

We further investigated i.t. heat-iMVA in a newly established PRC2-isogenic murine MPNST model (SKP605) amenable for syngeneic transplantation that resembles human MPNSTs (Figure 5 and Supplemental Figure 4). In the PRC2-wt context, SKP605 (sg*Con*) tumors already had low levels of tumor immune infiltrates, which were further diminished with PRC2 loss (*sgEed*) (Figure 5E); both PRC2-wt and PRC2-loss SKP605 tumors were resistant to ICB treatment (Supplemental Figure 8F). Using the murine MPNST model, we observed that IT heat-iMVA sensitized the PRC2-loss SKP605 tumors to ICB treatment and significantly prolonged survival (Figure 8, J and K). Notably, 7 of 10 mice treated with the combined i.t. Heat-iMVA and ICB had a complete response (CR) (Figure 8J). Moreover, we did not observe any tumor regrowth when the 7 mice with a CR were rechallenged with SKP605 (sg*Eed*) tumor cells on the opposite side of the previously treated tumor grafts (Figure 8L), suggesting an adaptive immune response. These results demonstrate that i.t. delivery of immunogenic MVA therapy combined with ICB was an effective initial strategy to modify the cold TME and elicit an antitumor effect in PRC2-loss tumors.

## Discussion

Unlike previously described mechanisms of tumor cell–intrinsic immune evasion that primarily affect selective subpopulations of tumor immune infiltrates by, for example, decreasing T lymphocyte infiltration and function or recruiting myeloid-suppressive cells (e.g., *LKB1* mutation in lung cancer) (47, 51–59), our study showed that PRC2 loss in tumors affected a broad spectrum of subpopulations of immune infiltrates, including DCs, T cells, B cells, and/or macrophages, and therefore drives an immune-desert TME, consistent with a prior proteomics study of MPNST (60). Rather than influencing MHC-I and MHC-II gene expression directly (29, 60), we found that PRC2 genetic inactivation in tumors led to a reprogrammed H3K27ac enhancer and promoter landscape and pervasive shifts of multiple chromatin templated processes, particularly activation of WNT/β-catenin signaling, diminished IFN signaling responses, reduced chemokine production (e.g., *CCL2*), and, consequently, impaired antigen presentation. These effects were further amplified in vivo and ultimately resulted in an immune-desert TME with diminished recruitment of the major subclasses of immune cells and resistance to ICB treatment in both MPNSTs and other cancer types. Importantly, our study demonstrated that immunogenic therapeutic viruses could overcome a PRC2-loss–mediated immune-desert TME and sensitize PRC2-loss tumors to ICB treatment.

We have established and characterized a PRC2-isogenic SKP605 murine MPNST model amenable for syngeneic transplantation into C57BL/6J mice. This model harbors the characteristic genetic alterations of and exhibits the histopathological and TME features of human MPNSTs. We noted some differences in tumor immune filtrates between the murine and human MPNSTs, e.g., B220^+^ tumor–infiltrating B cells that appeared more enriched in murine MPNSTs compared with human tumors (Figure 1, D and E, and Figure 5F), probably due to host differences between mice and humans, syngeneic transplant systems, a lack of full recapitulation of the entire genetic alteration landscape, and the differential timing of tumor development. Nevertheless, we believe this model represents a valuable resource for the NF1 and sarcoma community for future mechanistic and therapeutic investigations of MPNSTs in immunocompetent hosts.

Mechanistically, PRC2 loss in tumor cells led to genome-wide redistribution of chromatin accessibility and chromatin modifications (e.g., H3K27ac), and hence reprogramming of the chromatin landscape. Although with PRC2 loss, global H3K27me2/3 histone modifications were lost, with a reciprocal global gain of H3K27ac on the chromatin, the genome-wide change in H3K27ac was not uniform, with distinct genomic regions of increased and decreased H3K27ac enrichment, as well as an increase in diffuse H3K27ac signals over large genomic territories. The global increase in H3K27ac was not due to increased enzymatic activity of CBP/P300, the canonical histone acetyltransferase (HAT) for H3K27ac, as other classical CBP/P300 substrates, such as H3K9ac and H3K18ac, had remained stable with PRC2 loss (data not shown). It is conceivable that the global increase in H3K27ac levels was due to an increased availability of unmodified H3K27 substrate because of H3K27me1/2/3 loss in PRC2-loss tumors. Moreover, global H3K27me1/2/3 loss led to a redistribution and spreading of H3K27ac from adjacent H3K27ac-enriched regions to previously H3K27me3-enriched regions and resulted in dampened enrichment at adjacent preexisting H3K27ac regions (Supplemental Figure 2, H and N). Consistent with chromatin accessibility changes mediated by PRC2 loss, there were many genes with markedly increased H3K27ac enrichment at distal regulatory enhancers and intergenic regions with a corresponding increase in transcriptomes that included master regulators, developmental and lineage specification pathways, and, importantly, genes involved in the WNT signaling pathway that had previously been shown to actively suppress T cell recruitment and affect the local antitumor response through defects in T cell priming (47, 57, 58, 61). The decreased H3K27ac enrichment mediated by PRC2 loss mainly occurred at active promoters and distal regulatory enhancers including existing SEs in PRC2-wt tumor cells and correlated with the decrease in transcriptomes. This negative impact of H3K27ac enrichment preferentially affected the immune signaling pathways, including those for chemotactic cytokines (e.g., *CCL2*) and type I and type II IFN response genes, leading to significantly (*P* < 0.05) diminished expression of chemokines and dampened IFN-γ signaling responses in vitro. The diminished antitumor immune response was further amplified in vivo. Thus, in the relevant cell context, PRC2 loss reprogrammed the chromatin landscape and shifted a baseline primed immune signaling–dependent cellular response to the PRC2-regulated development and cellular differentiation transcriptional programs. Through these mechanisms, PRC2 loss reprogrammed the tumor cell and the TME to decrease antigen presentation and reduce chemotactic cytokine secretion, leading to diminished tumor immune infiltrates and ICB primary resistance. Our observations are consistent with recent reports demonstrating that inactivating mutations of the core components, e.g., *PBRM1* of the PBAF complex, that are antagonistic to the PRC2 complex in tumor cells enhanced IFN-γ signaling and sensitized the *PBAF*-deficient tumors to ICB therapies (62, 63).

Virus-based cancer immunotherapy is a promising strategy to induce host antitumor immunity through multiple mechanisms, including virus-induced oncolysis and alteration of the immunosuppressive TME (64, 65). MVA is a highly attenuated vaccinia strain that has been approved by the FDA as a safe and effective vaccine against smallpox and monkeypox and has been investigated as a promising vaccine vector (66, 67). Infection of tumor and immune cells by i.t. delivery of heat-iMVA has been shown to lead to a robust induction of type I IFN and proinflammatory cytokines and chemokines to attract and activate immune effector cells (50). Here, we showed that i.t. heat-iMVA disrupted immune tolerance and activated immune cells to eliminate malignancies in PRC2-loss tumors, particularly when combined with other immunotherapies. Furthermore, the engineered next-generation MVA created by deletion of immunosuppressive viral genes and exogenous expression of T cell–activating cytokines is currently being developed and will be evaluated clinically in PRC2-loss tumors.

## Methods

### Human tumor tissue collection.

Clinical samples were collected during surgical resection from patients with MPNSTs and other cancers according to MSK IRB protocols. Frozen and paraffin-embedded tissue samples were banked, and TMAs were generated. All MPNSTs and other PRC2-loss cancers were pathologically reviewed and confirmed by an MSK pathology expert. Sample annotations are shown in Supplemental Table 1.

### Cell lines.

HEK-293T and sNF96.2 cell lines were purchased from the American Type Culture Collection (ATCC). The human MPNST cell line M3 (*NF1^–/–^*, *CDKN2A/B^–/–^*) was a gift and derived from an NF1-associated MPNST by William Gerald (MSK). The murine MPNST cell line, SKP605 (*Nf1^–/–^*, *Cdkn2a/b^–/–^*), was generated in-house from skin-derived precursors (SKPs) according to a previously published protocol (46) and confirmed by a pathologist. The murine breast cancer cell line AT3 was obtained in-house. All cell lines were confirmed as mycoplasma free. All cell lines were cultured in DMEM supplemented with l-glutamine (2 mM), penicillin (100 U/mL), streptomycin (100 μg/mL), and 10% heat-inactivated FBS in 5% CO_2_ at 37°C.

### IHC.

Human tissue processing, embedment, sectioning, and staining with H&E were performed at the MSK Department of Pathology. IHC of human TMA tumor samples was performed using a Ventana BenchMark ULTRA Automated Stainer at the MSK Human Oncology and Pathogenesis Program (HOPP) automatic staining facility. Mouse fresh tissues were fixed in 4% paraformaldehyde (PFA) overnight, washed 3 times with PBS for 5 minutes each time, and stored in 70% ethanol. Tissue paraffin embedment, sectioning, and H&E staining were performed by Histoserv Inc. IHC of CD45 (Cell Signaling Technology [CST], catalog 70257, 1:100) was performed at the MSK Molecular Cytology Core Facility. IHC stainings for H3K27me3 (MilliporeSigma, catalog 07-449, 1:500, protocol no. 313), Ki67 (Abcam, catalog ab15580, 1:500, protocol no. 312), and S100B (Abcam, catalog ab52642, 1:2000, protocol no. 313) were performed using the Ventana BenchMark ULTRA Automated Stainer. Slides were scanned at the MSK Molecular Cytology Core Facility and analyzed using CaseViewer software.

### Gene knockout by CRISPR/Cas9.

The pLCP2B plasmid was generated by removing Cas9-P2A-tRFP from pL-CRISPR.EFS.tRFP (Addgene, no. 57819) and replaced with Cas9-P2A-Blast. The lentiCRISPR-v2 vector with puromycin was purchased from Addgene (no. 52961). The sgRNA oligonucleotides described below were engineered into vectors using the standard CRISPR/Cas9 knockout protocol. M3 sg*Con* cells were pooled from 6 single-cell clones, and M3 sg*SUZ12* cells were pooled from 9 single-cell clones. Both SKP605 sg*Con* cells and sg*Eed* cells were pooled from 4 single-cell clones. See the Supplemental Methods for details on the methods used and sgRNA and sequences.

### Transplant mouse model.

Female C57BL/6J (6–8 weeks of age) mice were purchased from the The Jackson Laboratory (stock no. 000664), and female NSG (6–8 weeks of age) mice were purchased from the MSK animal core facility. PRC2-isogenic M3 cells (3 million cells) and SKP605 cells (5 million cells) in 100 μL 1:1 PBS/Matrigel (Corning, catalog 356237) were orthotopically transplanted into the sciatic nerve pockets of NSG and C57BL/6J mice, respectively. For heat-iMVA treatment, PRC2-isogenic SKP605 cells (1 million cells in 100 μL 1:1 PBS/Matrigel) were s.c. grafted onto the flanks of C57BL/6J mice. PRC2-isogenic AT3 cells (100,000–150,000 cells in 100 μL 1:1 PBS/Matrigel) were orthotopically transplanted into the mammary fat pads of C57BL/6J mice. For tumor growth, tumors were measured twice weekly by Vernier caliper and calculated as follows: tumor volume (TV) = 4/3 pi × length/2 × width/2 × height/2. For survival studies, mice were euthanized when the TV reached 1500 mm^3^ for AT3 murine mammary tumors and 300 mm^3^ for SKP605 s.c. tumors or when the mice exhibited signs of illness and discomfort that required euthanasia.

### Monoclonal antibody therapy.

The monoclonal antibodies anti–PD-1 (250 μg, Bio X Cell, CD279, catalog BE0146); anti-CTLA4 (200 μg, Bio X Cell, CD152, catalog BE0164); and anti-2A3 (100 μg, Bio X Cell, catalog BE0089) in 100 μL PBS were delivered via i.p. injection every 3 days. The doses of ICB in combination with heat-iMVA were 125 μg anti–PD-1 plus 100 μg anti-CTLA4. The depletion monoclonal antibodies anti–mouse CD4 (Bio X Cell, GK1.5, catalog BE0003-1); anti–mouse CD8α (Bio X Cell, 2.43, catalog BE0061); anti–mouse NK1.1 (Bio X Cell, PK136, catalog BE0036); and anti–mouse IFN-γ (Bio X Cell, XMG1.2, catalog BE0055), started 2 days prior to ICB treatment, were delivered i.p. once every 3 days at 200 μg in 100 μL PBS for each dose.

### Viruses and intratumoral injection.

The MVA was provided by G. Sutter (University of Munich, Munich, Germany). MVA was propagated in BHK-21 cells (baby hamster kidney cell, ATCC, catalog CCL-10) and purified through a 36% sucrose cushion. Heat-iMVA was generated by incubating purified MVA at 55°C for 1 hour. Seven to 11 days after implantation, tumors were measured and heat-iMVA (an equivalent of 4 × 10^8^ PFU) in 200 μL or PBS was delivered via i.t. injection twice weekly, and the mice were subsequently monitored daily.

### OVA model antigen system.

The pMSCV-EGFP-PGK-Luc2-2A-USA plasmid, which included OT-I– and OT-II–binding antigens for MHC on a C57BL/6J genetic background, was obtained in-house. To make the retrovirus, HEK293T cells were transfected with pMSCV-EGFP-PGK-Luc2-2A-USA and the packaging plasmids pVSVg (Addgene, catalog 8454) and pEco (Takara, catalog PT3749-5). Cells transduced with the retrovirus were sorted for EGFP positivity by flow cytometry, and these cells stably overexpressed OT-I and OT-II–binding OVA model antigens. APC anti–mouse H-2Kb bound to the SIINFEKL antibody (BioLegend, catalog 141605) was used to detect the OVA model antigen on the cancer cell surface. To detect OVA-specific T cell priming, OVA^+^ and OVA^–^ AT3 cells were orthotopically grafted into mammary fat pads of C57BL/6 mice. Eighteen days after grafting, cells isolated from TdLNs were incubated with 2 μg/mL OVA 257-264 in T cell medium for 24 hours at 37°C. First, cells were stained with an Fc-blocking antibody for 15 minutes and then with an Fc-blocking antibody plus iTAg H-2Kb OVA Tetramer-SIINFEKL-APC (MBL International, catalog TB-5001-2) in FACS buffer for 1 hour at room temperature and protected from light. Next, live/dead dye (TONBO, catalog 13-0863-T100) and anti–mouse CD8a-FITC (TONBO, catalog 35-1886-U100) were added to the staining system for 30 minutes at 4°C. Finally, cells were washed with FACS buffer 3 times and analyzed by FACS.

### Protein extraction and Western blotting.

Sample preparation and Western blotting were performed as described previously (31) and in the Supplemental Methods. The following primary antibodies were used for immunoblotting: anti-NF1 (1:2000, Bethyl Laboratories, catalog A300-140A); anti-CDKN2A (1:1000, Delta BioLabs, catalog DB018); anti-CDKN2B (1:500, Abcam, catalog ab53034); anti-SUZ12 (1:1000, CST, catalog 3737); anti-H3K27me3 (1:2000, CST, catalog 9733); anti-H3K27me2 (1:5000, CST, catalog 9728); anti-H3K27me1 (1:1000, Takara, catalog MABI0321-100I); anti-H3K27ac (1:4000, Abcam, catalog ab4729), anti–histone H3 (1:2000, CST, catalog 12648); anti–β-actin (1:5000, Proteintech, catalog 66009-1-Ig); anti–lamin B1 (1:2000, Proteintech, catalog 12987-1-AP); anti-HIRA (1:1000, Active Motif, catalog 39557); and anti-GAPDH (1:3000, CST, catalog 5174S). See complete unedited blots in the supplemental material.

### RNA isolation and quantitative reverse transcription PCR.

Total RNA was isolated from cell lines using the Total RNA kit I (Omega, catalogR6834-02) and homogenizer mini columns (Omega, catalog HCR003), or from tissues using TRIzol (Thermo Fisher Scientific, catalog 15596026). cDNA was prepared using High-Capacity cDNA Reverse Transcription Kit (Thermo Fisher Scientific, catalog 4368814). Quantitative reverse transcription PCR (qRT-PCR) was performed using SYBR Green Master Mix (Thermo Fisher Scientific, catalogA25777) with a V7 Real-Time PCR system (Applied Biosystems). Expressed values relative to the control were calculated using the ΔΔCt method. Housekeeping genes, e.g., *RPL27* and *Rpl27*, were used as reference genes for normalization. The qRT-PCR primer sequences are listed in the Supplemental Methods.

### RNA-Seq and analysis.

Poly-A capture RNA-Seq of total RNA isolated from fresh tissues or cells using TRIzol was performed at the MSK Integrated Genomics Operation (IGO) facility on an Illumina HiSeq 2500 platform with 50 bp paired-end (human MPNST tumors) or single-end (AT3 tumors) reads to obtain a minimum yield of 40 million reads per sample. The sequence data were processed and mapped to the human reference genome (hg19) or mouse reference genome (mm9) using STAR, version 2.3 (68). Gene expression was quantified as transcripts per million (TPM) using STAR (69) and log_2_ transformed. GSEA was performed using the JAVA GSEA 2.0 program (70).

### ATAC-Seq and analysis.

ATAC-Seq was performed at the Center for Epigenetics Research as previously described (71, 72). For each sample, nuclei from 50,000 cells were prepared and incubated with 2.5 μL transposase (Illumina) in a 50 μL reaction for 30 minutes at 37°C. After purification of transposase-fragmented DNA, the library was amplified by PCR and subjected to paired-end, 50 bp high-throughput sequencing on an Illumina HiSeq 2500 platform. For data analysis, ATAC-Seq reads were quality and adapter trimmed using Trim Galore before alignment to the human reference genome (hg19) with Bowtie2 using the default parameters. Motif signatures were obtained using HOMER, version 4.5 (http://homer.ucsd.edu). See the Supplemental Methods for details on the analysis.

### ChIP-Seq, CUT&RUN, and analysis.

Chromatin isolation from the indicated cells and immunoprecipitation were performed as previously described (73). The libraries were sequenced on an Illumina HiSeq 2500 platform with 50 bp paired-end reads. Reads were trimmed using Trim Galore software and then aligned to the human genome (hg19) using Bowtie2 alignment software, version 2.3.5 (74). Duplicated reads were eliminated for subsequent analysis. Peak calling for H3K27ac was performed using MACS2 software, version 2.1.1 (75) in paired mode and comparing ChIP samples to the input, using an FDR of *q* < 10^–3^. H3K27me3 peaks were called using a sliding window approach to find regions enriched with H3K27me3 compared with input reads. Spike-in was used in H3K27me3 ChIP-Seq for normalization because of the global loss of H3K27me3 after PRC2 loss. We discarded peaks mapped to blacklisted genomic regions identified by ENCODE (76, 77). The primary antibodies used for ChIP were anti-H3K27me3 (CST, catalog 9733) and anti-H3K27ac (Abcam, catalog ab4729).

The H3K27ac peaks were separated into promoters and distal and intergenic peaks as described previously (78). They were also used for SE analysis using the ROSE R package (option: –t 2500) (79, 80). The peaks from controls and sg*SUZ12* samples were merged to generate a nonoverlapping list of union peaks. ChIP-Seq reads located to the merged peaks were calculated and used by DESeq2 software to identify peaks with differential modifications at an adjusted *P* value of less than 0.05 and a fold change of greater than 2. The markedly increased or decreased peaks in the sg*SUZ12* samples at promoters and nonpromoter regions were subjected to independent transcription factor binding motif analysis with HOMER software (version 4.7, default parameter) (81), using all peaks as a background. The fold changes in these peaks were also compared with gene expression changes in the RNA-Seq analysis for each of the promoter and distal peaks assigned to genes (note that 1 gene could have multiple peaks).

The CUT&RUN assay was performed using the commercial kit and protocol (CST, catalog 86652). The primary antibodies (1 μg per assay) used for the CUT&RUN assay were as follows: anti-H3K27me3 (CST, catalog 9733); anti-H3K27ac (Abcam, catalog ab4729); anti-H3K36me2 (Thermo Fisher Scientific, catalog MA5-14867); and anti-H3K36me3 (Active Motif, catalog 61021).

### Data availability.

All sequencing data were deposited in the NCBI’s Gene Expression Omnibus (GEO) database: RNA-Seq of human MPNST tumor tissues (GEO GSE206527); RNA-Seq of AT3 tumors (GEO GSE179703); ATAC-Seq of M3 cells (GEO GSE179699); ChIP-Seq of M3 cells (GEO GSM5420909, GSM5420912, GSM5420919, GSM5420922); and CUT&RUN of M3 cells (GEO GSE202555).

### Statistics.

All statistical analyses were performed using GraphPad Prism 7 or 9 (GraphPad Software). Unless otherwise noted in the figure legends, all data are shown as the mean ± SEM combined with a 2-tailed, unpaired *t* test for statistical comparisons between 2 groups, 1-way ANOVA for comparisons of more than 2 groups, and a log-rank (Mantel-Cox) test for survival analyses. A *P* value of less than 0.05 was considered statistically significant. All experiments shown were repeated at least twice.

### Study approval.

Clinical samples were collected according to MSK IRB protocols (IRB nos. 02-060 and 06-107). All procedures related to mouse handling, care, and treatments were performed according to guidelines approved by the IACUC of MSK.

## Author contributions

JY, Yuedan Chen, PC, and Yu Chen conceived the project and designed the experiments. JY, Yuedan Chen, SW, CJL, JS, CRA, SS, PC, and Yu Chen collected clinical samples. JY and Yuedan Chen performed most of the in vitro and in vivo experiments with assistance from AJP, BGN, EWPW, and MAMR and technical support from SW, CJL, MRP, EG, and JS for the in vivo experiments. JY, LD, NY, and YW performed the heat-iMVA–based viral therapy experiments. CRA and PC performed pathological and immunohistochemical review of the clinical and murine tumor samples. MOL, LD, and BGN provided additional conceptual advice, experimental design assistance, and technical support for the immunology experiments. DZ, RPK, JLVM, YL, PMG, FT, EK, Yu Chen, JY, and PC performed RNA-Seq, ChIP-Seq, CUT&RUN, ATAC-Seq, and integrative analyses. JY, PC, and Yu Chen wrote the manuscript. All authors reviewed and edited the manuscript.

## Supplementary Material

Supplemental data

Supplemental table 1

Supplemental table 2

Supplemental table 3

Supplemental table 4

Supplemental table 5

Supplemental table 6

## Version 1 07/19/2022

In-Press Preview

## Version 2 09/01/2022

Electronic publication
