# Supplementary material for: Tumor-intrinsic PRC2 inactivation drives a context-dependent immune-desert microenvironment and is sensitized by immunogenic viruses
Source: J Clin Invest. 2022 Sep 1;132(17):e153437. doi: 10.1172/JCI153437 (PMC9433107; doi:10.1172/JCI153437)
Supplement: Supplemental data [file jci-132-153437-s039.pdf]

Supplementary information for

Tumor-intrinsic PRC2 inactivation drives a context-dependent immune-desert microenvironment and is sensitized by immunogenic viruses

Juan Yan and Yuedan Chen *et al.*

(Date: 07/07/2022)

* Corresponding authors: Dr. Yu Chen, Phone: 646-888-3356, Email: cheny1@mskcc.org. Dr. Ping Chi, Phone: 646-888-3338, Email: chip@mskcc.org. Address: Memorial Sloan Kettering Cancer Center, 1275 York Avenue, New York, NY 10065.

The PDF file includes:

Supplementary Figures. 1 to 8 and figure legends

Supplementary Methods

Supplementary figures and figure legends

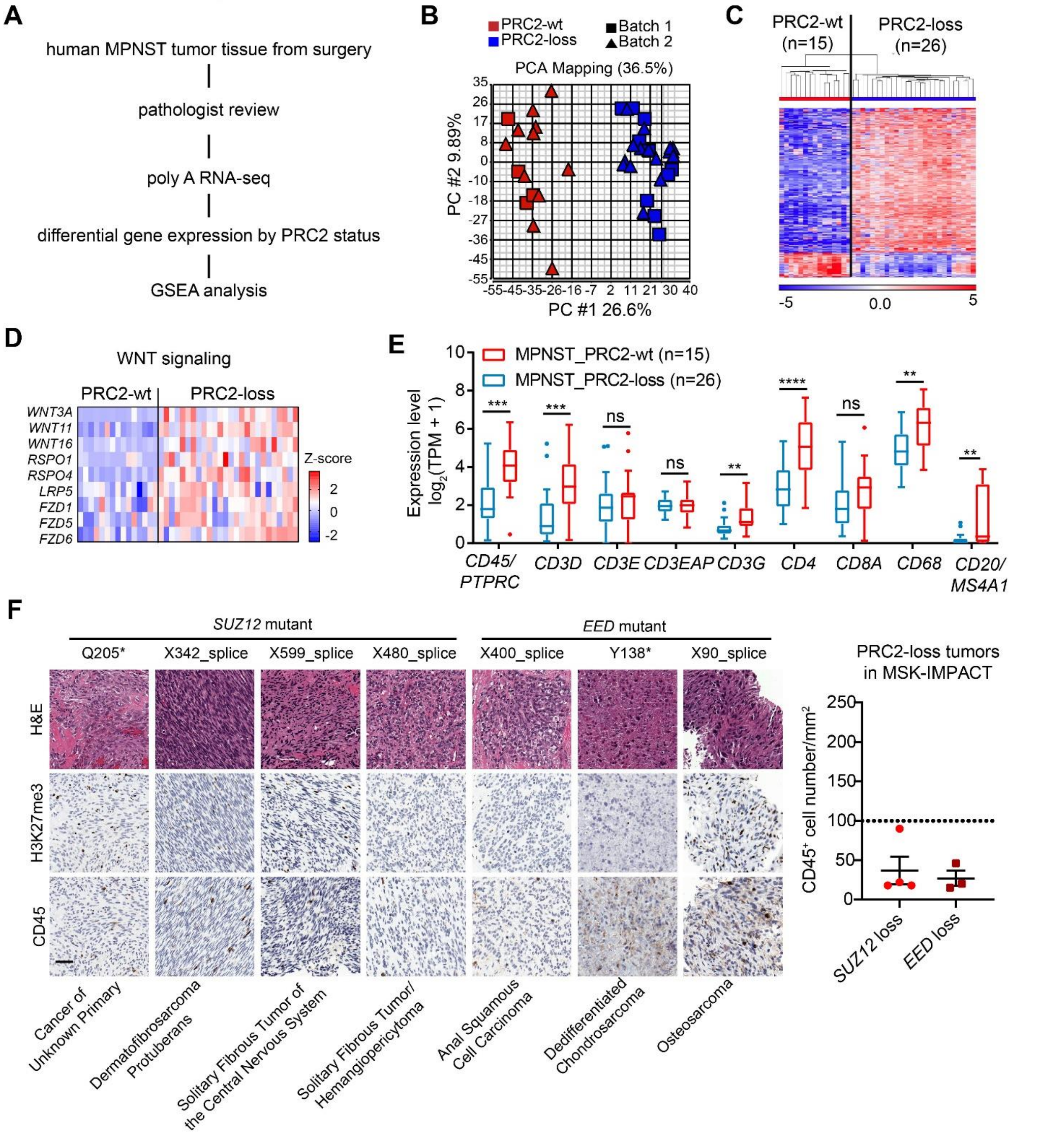

Supplementary Figure 1. Human PRC2-loss tumors are associated with an immune-desert microenvironment, Related to Figure 1. (A) A schematic of human MPNST tumor tissue sample handling and processing. (**B**) Principal component analysis (PCA) of all (1st and 2nd batch) tumor transcriptomes by RNA-seq. (**C**) Hierarchical clustering of the most differentially regulated genes (FDR $q<0.05$, and fold-change≥8) between PRC2-loss and PRC2-wt MPNST tumors in RNA-seq. (**D**) Heatmap of WNT signaling pathway genes in human MPNSTs by RNA-seq. (**E**) Tukey's box

and whiskers plots of mRNA expression levels of immune maker genes in human MPNST by RNA-seq. TPM: transcripts per million. ****P*<0.001, *****P*<0.0001 by unpaired two-tailed t test. (**F**) Representative IHC images (left) and quantification of the $CD45^+$ immune cell population (right) of human PRC2-loss tumors identified by MSK-IMPACT. Scale bar: 50μm. All error bars: mean ± SEM.

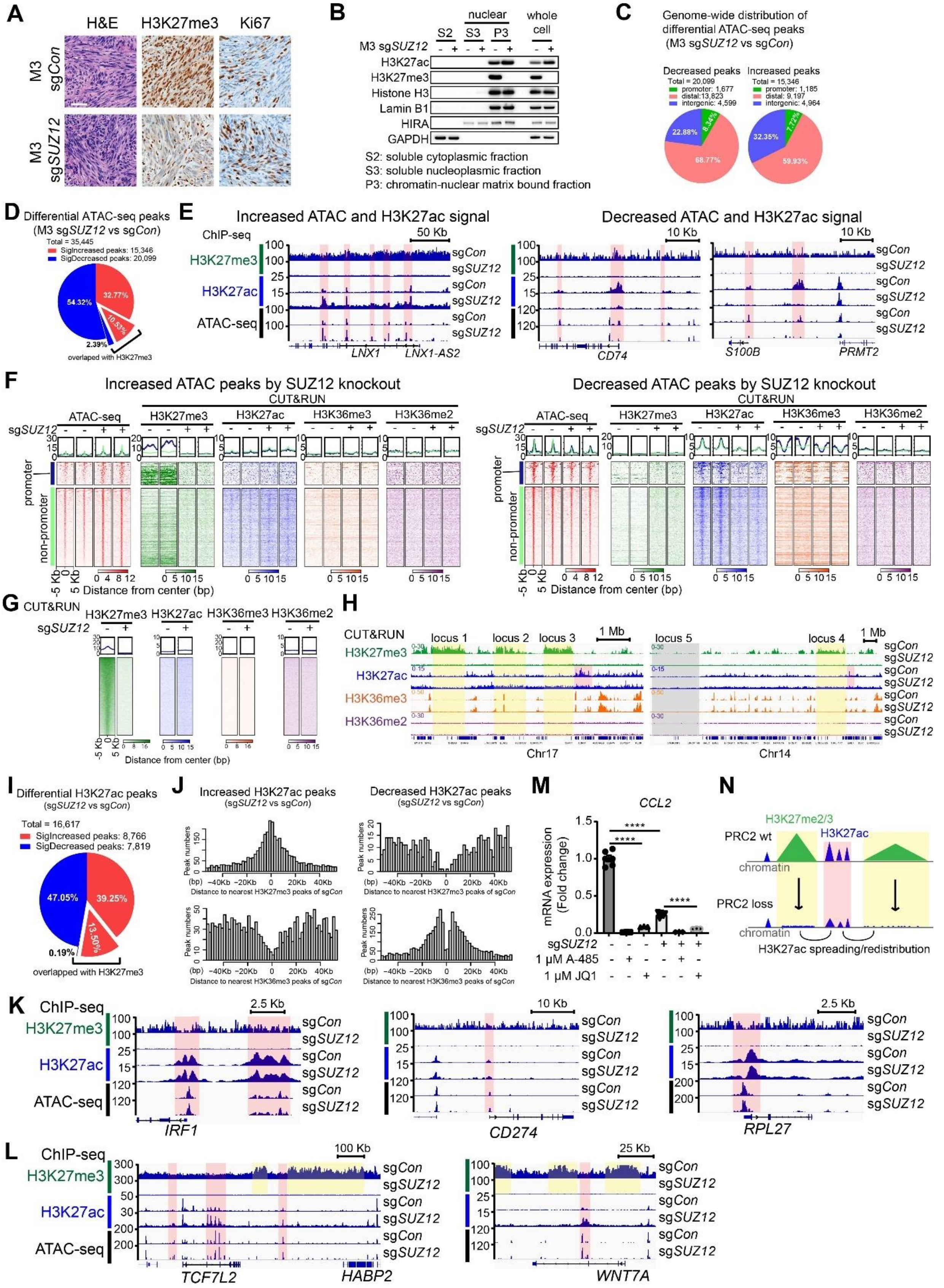
A
H&E
H3K27me3
Ki67
M3 sgCon
M3 sgSUZ12
B
nuclear
S2
S3
P3
whole cell
M3 sgSUZ12
H3K27ac
H3K27me3
Histone H3
Lamin B1
HIRA
GAPDH
S2: soluble cytoplasmic fraction
S3: soluble nucleoplasmic fraction
P3: chromatin-nuclear matrix bound fraction
C
Genome-wide distribution of differential ATAC-seq peaks (M3 sgSUZ12 vs sgCon)
Decreased peaks
Total = 20,099
promoter: 1,677
distal:13,823
intergenic: 4,599
Increased peaks
Total = 15,346
promoter: 1,185
distal: 9,197
intergenic: 4,964
D
Differential ATAC-seq peaks (M3 sgSUZ12 vs sgCon)
Total = 35,445
SigIncreased peaks: 15,346
SigDecreased peaks: 20,099
overlaped with H3K27me3
E
Increased ATAC and H3K27ac signal
Decreased ATAC and H3K27ac signal
ChIP-seq
H3K27me3
H3K27ac
ATAC-seq
LNX1
LNX1-AS2
CD74
S100B
PRMT2
F
Increased ATAC peaks by SUZ12 knockout
Decreased ATAC peaks by SUZ12 knockout
CUT&RUN
ATAC-seq
H3K27me3
H3K27ac
H3K36me3
H3K36me2
promoter
non-promoter
Distance from center (bp)
G
CUT&RUN
H3K27me3
H3K27ac
H3K36me3
H3K36me2
Distance from center (bp)
H
CUT&RUN
locus 1
locus 2
locus 3
1 Mb
locus 5
locus 4
Chr17
Chr14
I
Differential H3K27ac peaks (sgSUZ12 vs sgCon)
Total = 16,617
SigIncreased peaks: 8,766
SigDecreased peaks: 7,819
overlapped with H3K27me3
J
Increased H3K27ac peaks (sgSUZ12 vs sgCon)
Decreased H3K27ac peaks (sgSUZ12 vs sgCon)
Peak numbers
Distance to nearest H3K27me3 peaks of sgCon
Distance to nearest H3K36me3 peaks of sgCon
M
CCL2
mRNA expression (Fold change)
sgSUZ12
1 μM A-485
1 μM JQ1
N
H3K27me2/3
H3K27ac
PRC2 wt
chromatin
PRC2 loss
H3K27ac spreading/redistribution
K
ChIP-seq
H3K27me3
H3K27ac
ATAC-seq
IRF1
CD274
RPL27
L
ChIP-seq
H3K27me3
H3K27ac
ATAC-seq
TCF7L2
HABP2
WNT7A

Supplementary Figure 2. H3K27ac change correlates the chromatin accessibility and transcription change after PRC2 loss in cancer cells, Related to Figure 3. (**A**) Representative IHC of indicated proteins in orthotopically (sciatic nerve pocket) transplanted PRC2-isogenic human MPNST tumors. Scale bar: 50µm. (**B**) Immunoblots of indicated proteins from different fractions of PRC2-isogenic M3 cells. (**C**) Genome-wide distribution of significantly decreased (left) and increased (right) chromatin accessibility sites. promoter (TSS ± 2 kb), distal regulatory (-50 kb from TSS TES + 5 kb) and intergenic (non-promoter, non-distal regulatory) regions. (**D**) Pie chart of the overlap of the significantly changed ATAC-seq peaks with H3K27me3 enrichment. (**E)** ChIP-seq and ATAC-seq profiles at the loci of selective genes with increased (left) and decreased (right) H3K27ac enrichment and chromatin accessibility in PRC2-isogenic M3 cells. (**F-G**) Density plot of various histone modifications by CUT&RUN, centered on significantly changed ATAC peaks (F) and H3K27me3 peaks (G) in PRC2-isogenic M3 cells. (**H**) CUT&RUN profiles of indicated histone modifications at the selective loci in PRC2-isogenic M3 cells. Yellow: regions with H3K27me3 changes; Pink: regions with H3K27ac changes; Grey: background genomic region. (**I**) Pie chart of the overlap of the significantly changed H3K27ac peaks with H3K27me3 enrichment in PRC2-isogenic M3 cells. (**J**) The genomic distance of the significantly changed H3K27ac peaks to the nearest H3K27me3 and H3K36me3 peaks. (**K-L**) ChIP-seq and ATAC-seq profiles at the loci of indicated IFNγ-responsive genes and others in PRC2-isogenic M3 cells. Pink: H3K27ac change; Yellow: H3K27me3 change. *RPL27:* reference gene. (**M**) Relative changes in mRNA expression of *CCL2* assayed at 72 hours after indicated inhibitor treatment in M3 cells. (**N**) A schematic depicting the H3K27ac redistribution model with PRC2 and global H3K27me3 loss. ****P<0.0001 by unpaired two-tailed t test for two cohorts and by one-way ANOVA comparing multiple cohorts in M. All error bars: mean ± SEM.

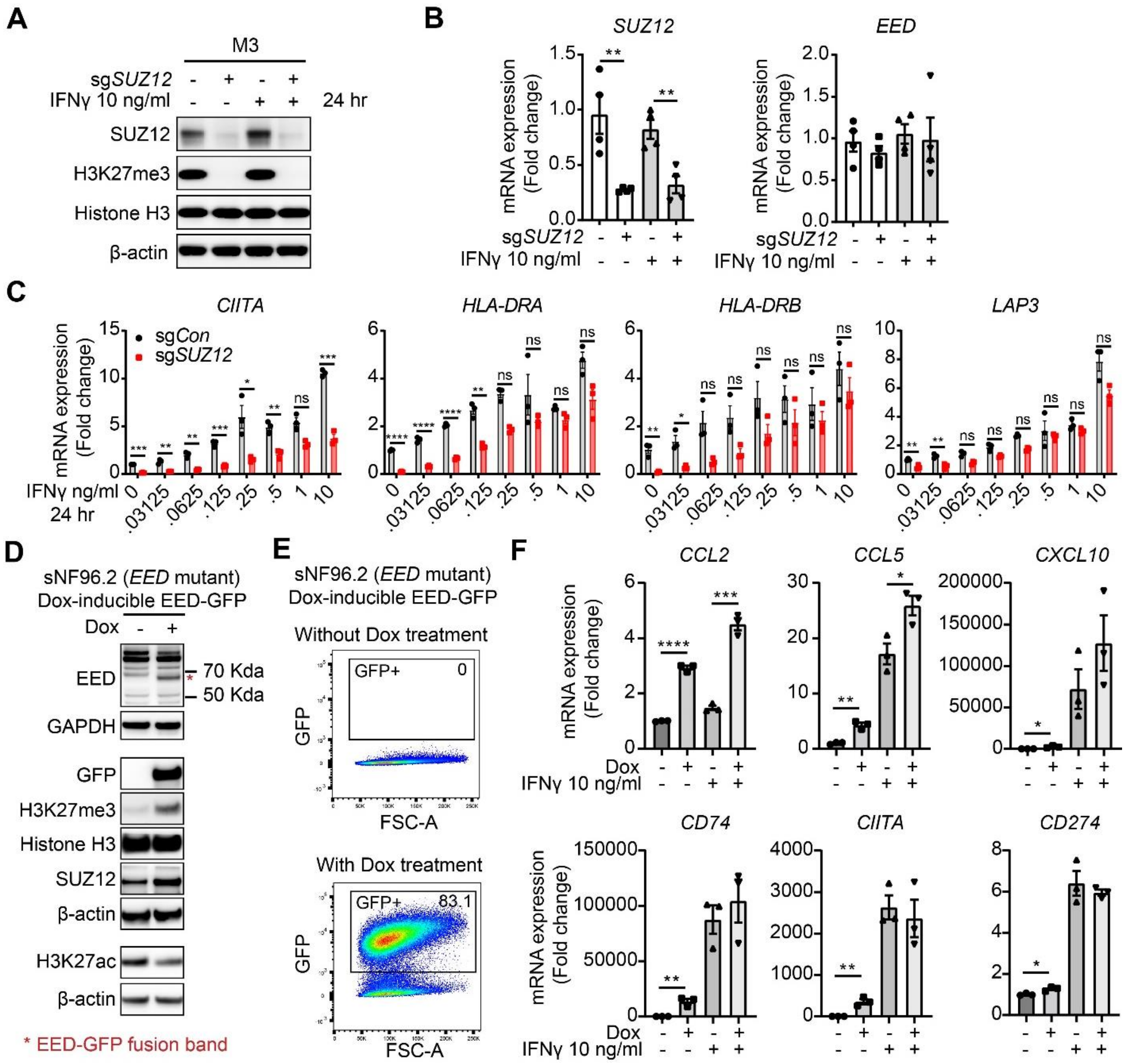

Supplementary Figure 3. IFNγ response is diminished by PRC2 loss in human MPNST cells, Related to Figure 4. (**A-B**) Immunoblots of indicated proteins (A) and relative changes in mRNA expression of S*UZ12* and *EED* by qRT-PCR (B, n=4) in PRC2-isogenic M3 cells stimulated with IFNγ (10 ng/ml) for 24 hours. (**C**) IFNγ dose-dependent relative changes in mRNA expression of IFNγ-responsive genes by qRT-PCR. n=3. (**D-E**) Validation of sNF96.2 cells harboring endogenous *EED* mutation with doxycycline (Dox)-inducible EED-GFP by western blot (D) and flow cytometry (E). Dox: doxycycline 1μg/ml for 2 weeks. Immunoblots were derived from multiple gels, each with its own loading control as presented. (**F**) Relative changes in mRNA expression of IFNγ-responsive genes by qRT-PCR in PRC2-isogenic sNF96.2 cells in (D) and treated with IFNγ (10 ng/ml) as indicated for 24 hours. n=3. *P<0.05, **P<0.01, ***P<0.001, ****P<0.0001 by unpaired two-tailed t test in B and F, by multiple unpaired t test in C (P<0.05, FDR q<0.05 and fold change≥2 as significant). All error bars: mean ± SEM.

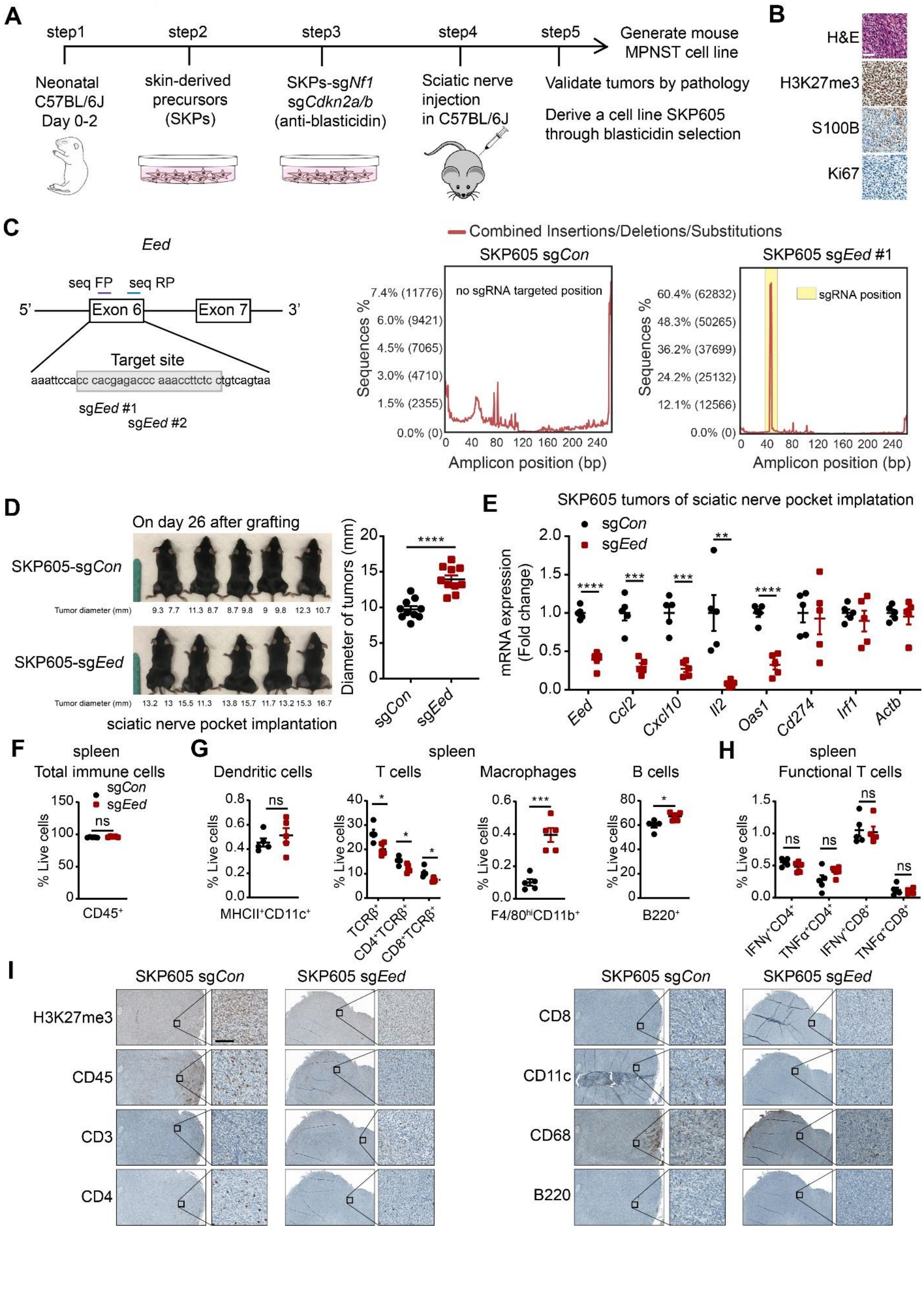

A
step1
step2
step3
step4
step5
Generate mouse MPNST cell line
Neonatal C57BL/6J Day 0-2
skin-derived precursors (SKPs)
SKPs-sgNf1 sgCdkn2a/b (anti-blasticidin)
Sciatic nerve injection in C57BL/6J
Validate tumors by pathology
Derive a cell line SKP605 through blasticidin selection
B
H&E
H3K27me3
S100B
Ki67
C
Eed
seq FP
seq RP
5'
Exon 6
Exon 7
3'
Target site
aaattccacc cacgagaccc aaaccttctc ctgtcagtaa
sgEed #1
sgEed #2
Combined Insertions/Deletions/Substitutions
SKP605 sgCon
no sgRNA targeted position
SKP605 sgEed #1
sgRNA position
Sequences %
Amplicon position (bp)
D
On day 26 after grafting
SKP605-sgCon
SKP605-sgEed
Tumor diameter (mm) 9.3 7.7 11.3 8.7 8.7 9.8 9 9.8 12.3 10.7
Tumor diameter (mm) 13.2 13 15.5 11.3 13.8 15.7 11.7 13.2 15.3 16.7
sciatic nerve pocket implantation
Diameter of tumors (mm)
sgCon
sgEed
E
SKP605 tumors of sciatic nerve pocket implatation
sgCon
sgEed
mRNA expression (Fold change)
Eed
Ccl2
Cxcl10
Il2
Oas1
Cd274
Irf1
Actb
F
spleen
Total immune cells
% Live cells
CD45+
G
spleen
Dendritic cells
T cells
Macrophages
B cells
MHCII+CD11c+
TCRβ+
CD4+TCRβ+
CD8+TCRβ+
F4/80hiCD11b+
B220+
H
spleen
Functional T cells
IFNγ+CD4+
TNFα+CD4+
IFNγ+CD8+
TNFα+CD8+
I
SKP605 sgCon
SKP605 sgEed
H3K27me3
CD45
CD3
CD4
CD8
CD11c
CD68
B220

Supplementary Figure 4. PRC2 loss promotes immune evasion in a murine MPNST model, Related to Figure 5. (**A**) A schematic of the development of a murine MPNST SKP605 model amenable for orthotopic (sciatic nerve) and syngeneic transplant in C57BL/6J mice. (**B**) Representative histology and IHC of indicated proteins in orthotopically transplanted murine MPNST tumors derived from step 5 in (A). Scale bar: 50µm. (**C**) Left: A schematic of *Eed* single guide RNA location and PCR primer location for amplicon-seq; Right, percent of mutations relative to amplicon position at sg*Eed* targeted region by CRISPR-sequencing in SKP605 cells. (**D**) Images (left) and tumor sizes (right) of orthotopically and syngeneic transplanted PRC2-isogenic (sg*Con* vs. sg*Eed*) SKP605 tumors explanted on day 26 post-transplant. Diameter = (length + width + height)/3. n=10 tumors per cohort. (**E**) Relative changes in mRNA expression of indicated genes by qRT-PCR in PRC2-isogenic SKP605 tumors in (D). n=5 tumors per cohort. (**F-H**) Percentage of $CD45^+$ cells (F), the subpopulations of immune cells (G), and functional T cells (H) of all live cells from the spleen of mice bearing PRC2-isogenic tumors. n =5 spleens per cohort. (**I**) Representative IHC images of immune cell populations in PRC2-isogenic SKP605 tumors in (D). Scale bar: 100µm. **P*<0.05, ***P*<0.01, ****P*<0.001, *****P*<0.0001 by unpaired two-tailed t test in D-H. All error bars: mean ± SEM.

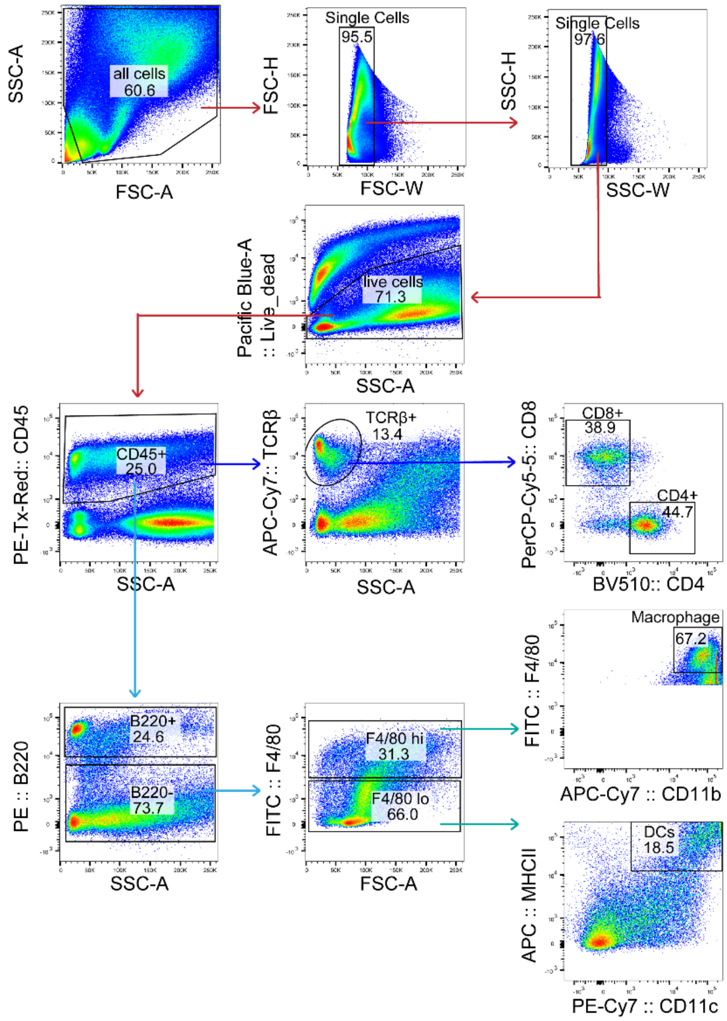

Supplementary Figure 5. Gating strategies of multi-color flow cytometry for immune profile, Related to Figure 5. Representative examples of CD45, B220, F4/80, MHCII, CD11c, CD11b, TCRβ, CD4, CD8 staining (gated for total immune cells, dendritic cells, T cells, macrophages, and B cells) for immune subpopulations analysis by FACS.

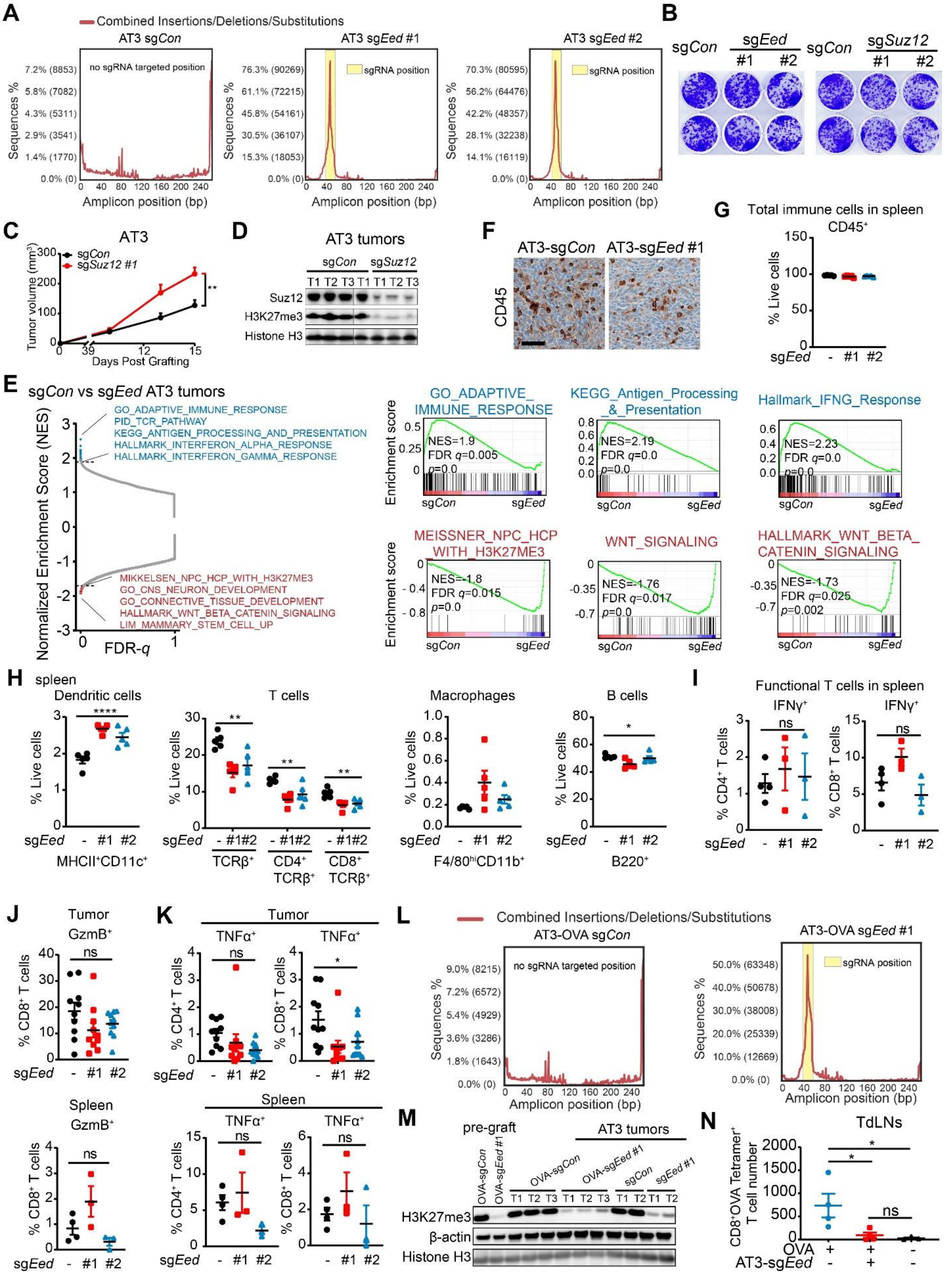
A
Combined Insertions/Deletions/Substitutions
AT3 sgCon
AT3 sgEed #1
AT3 sgEed #2
no sgRNA targeted position
sgRNA position
Sequences %
Amplicon position (bp)
B
sgCon
sgEed
sgSuz12
C
AT3
Tumor volume (mm³)
Days Post Grafting
D
AT3 tumors
Suz12
H3K27me3
Histone H3
E
sgCon vs sgEed AT3 tumors
Normalized Enrichment Score (NES)
FDR-q
GO_ADAPTIVE_IMMUNE_RESPONSE
PID_TCR_PATHWAY
KEGG_ANTIGEN_PROCESSING_AND_PRESENTATION
HALLMARK_INTERFERON_ALPHA_RESPONSE
HALLMARK_INTERFERON_GAMMA_RESPONSE
MIKKELSEN_NPC_HCP_WITH_H3K27ME3
GO_CNS_NEURON_DEVELOPMENT
GO_CONNECTIVE_TISSUE_DEVELOPMENT
HALLMARK_WNT_BETA_CATENIN_SIGNALING
LIM_MAMMARY_STEM_CELL_UP
Enrichment score
F
AT3-sgCon
AT3-sgEed #1
CD45
G
Total immune cells in spleen
CD45⁺
% Live cells
H
spleen
Dendritic cells
T cells
Macrophages
B cells
I
Functional T cells in spleen
J
Tumor
K
L
AT3-OVA sgCon
AT3-OVA sgEed #1
M
pre-graft
AT3 tumors
β-actin
N
TdLNs
CD8⁺OVA Tetramer⁺ T cell number

Supplementary Figure 6. PRC2 loss promotes immune evasion in a murine mammary tumor model, Related to Figure 6. (**A, K**) Percent of mutations relative to amplicon position at sg*Eed* targeted region by CRISPR-sequencing in AT3 (A) and AT3-OVA cells (K). **(B)** Clonogenic assays of PRC2-isogenic AT3 cells *in vitro*. (**C**) Tumor growth curves of orthotopically (mammary fat pad) transplanted PRC2-isogenic (sg*Con* vs. sg*Suz12*) AT3 mammary tumors in C57BL/6J mice. n=10 tumors for each cohort. (**D**) Immunoblots of indicated proteins in representative AT3 tumors in (C). (**E**) GSEA of transcriptomes derived from PRC2-isogenic AT3 (sg*Con* vs. sg*Eed*) tumors in Figure 6B. sg*Con*: n=4 tumors; sg*Eed*: n=8 tumors pooled from 4 sg*Eed* #1 and 4 sg*Eed* #2 tumors. (**F**) Representative IHC of $CD45^+$ immune cells in PRC2-isogenic AT3 tumors. Scale bar: 50µm. (**G-I**) Percentage of $CD45^+$ (G), subpopulations of immune cells (H), and functional T cells (I) of total live cells from the spleen of mice bearing PRC2-isogenic AT3 tumors. n=5 spleens per cohort. (**J-K**) Percentage of $GzmB^+$ (J) and $TNF\alpha^+$ (K) cells of $CD4^+$ or $CD8^+$ T cells in PRC2-isogenic AT3 tumors (upper) and spleens (lower). n=10 tumors per cohort; n=3-4 spleens per cohort. (**M**) Representative immunoblots of indicated proteins in pre-graft OVA-AT3 cells and explanted tumors. (**N**) Absolute cell numbers of MHCI-OVA $tetramer^+$ $CD8^+$ T cells in TdLNs related to Figure 6H. *P<0.05, **P<0.01, ****P<0.0001 by unpaired two-tailed t test in C, by one-way ANOVA in G-K, by one-way ANOVA corrected for multiple comparisons by two-stage linear step-up method of Benjamini, Krieger, and Yekutieli in N (FDR q<0.05 as significant) . All error bars: mean ± SEM.

Supplementary Figure 7

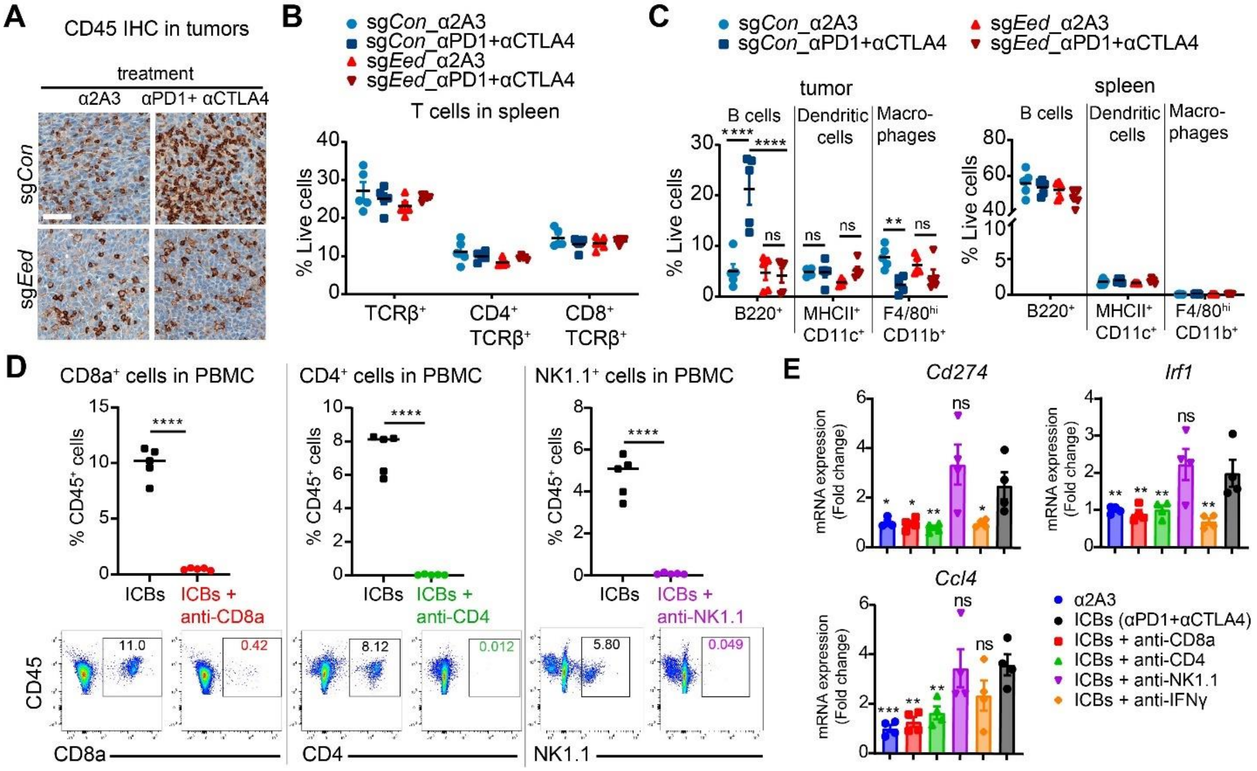

Supplementary Figure 7. PRC2 loss confers primary resistance to ICB in murine syngeneic transplant mammary tumor models, Related to Figure 7. (**A**) Representative IHC of CD45+ immune cells in PRC2-isogenic AT3 tumors related to Figure 7B. Scale bar: 50μm. (**B**) Percentage of T cells of all live cells in the spleens of mice related to Figure 7B. n=5 for each cohort. (**C**) Percentage of subpopulations of immune cells of all live cells in PRC2-isogenic AT3 tumors (left) and spleens (right) of mice related to Figure 7B. n=5 for each cohort. (**D**) The immune cell population change in PBMC after depletion antibody treatment for 19 days related to Figure 7H. (**E**) Relative changes in mRNA expression of indicated genes by qRT-PCR in tumors related to Figure 7H. *P<0.05, **P<0.01, ****P<0.0001 by one-way ANOVA corrected for multiple comparisons by two-stage linear step-up method of Benjamini, Krieger, and Yekutieli in B-C and E compared to ICBs group (FDR q<0.05 as significant), by unpaired two-tailed t test in D. All error bars: mean ± SEM.

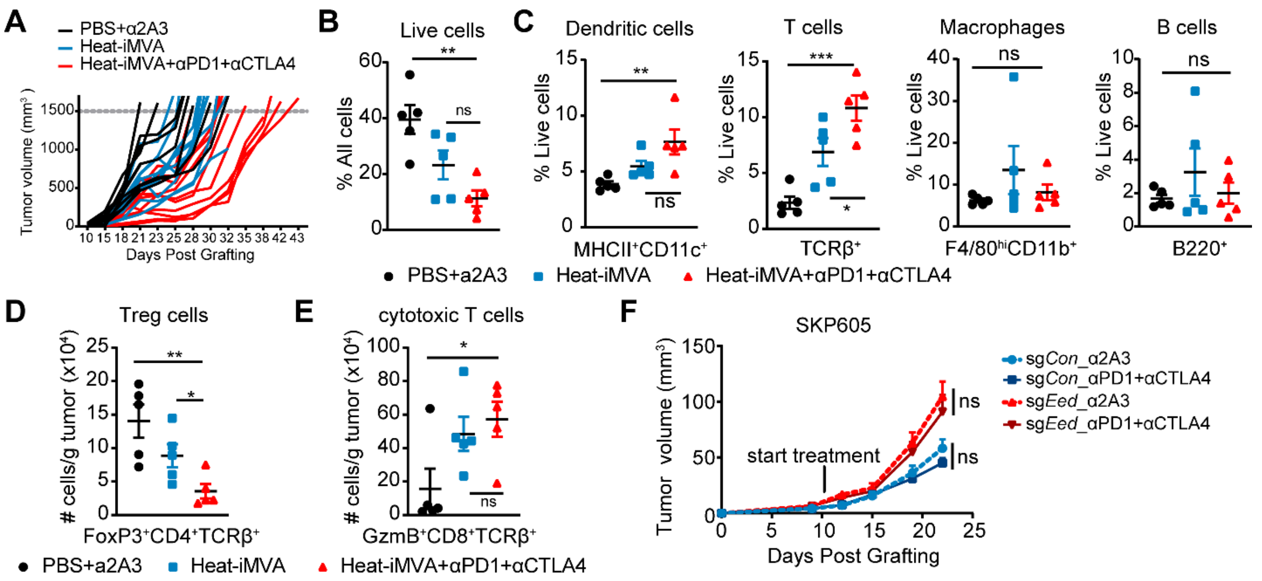

Supplementary Figure 8. Intratumoral injection of heat-iMVA combined with anti-PD1 and anti-CTLA4 retarded the tumor growth of PRC2-loss tumors, Related to Figure 8. (**A**) Tumor growth curves of individual AT3 (sg*Eed*) tumors under indicated treatment. n=10 tumors per cohort. (**B-E**) Percentage of live cells in all cells (B) and subpopulations of immune cells of all live cells (C), and number of Tregs (D) and cytotoxic T cells (E) per gram of tumors in AT3 (sg*Eed*) tumors under indicated treatment. n = 5 tumors for each cohort. (**F**) Tumor growth curves of SKP605 tumors under indicated treatment. n=6-8 tumors per cohort. *P<0.05, **P<0.01, ***P<0.001 by unpaired two-tailed t test in B-F between two cohorts; by one-way ANOVA in B-F comparing multiple cohorts. All error bars: mean ± SEM.

Supplementary Methods:

Gene knockout by CRISPR/Cas9

pLCP2B plasmid was generated by replacing the Cas9-P2A-tRFP with Cas9-P2A-Blast in the pL-CRISPR.EFS.tRFP (Addgene, #57819) vector. lentiCRISPR-v2 vector with puromycin was purchased from Addgene#52961. The sgRNA oligos as below were annealed, digested using *BsmB*I (New England BioLab, #R0580L) and cloned into vectors, e.g., pLCP2B or lentiCRISPR-v2. The lentivirus was generated by co-transfecting the transfer plasmids with the packaging plasmids pVSVg (Addgene, #8454) and psPAX2 (Addgene, #12260) into HEK-293T cells. To delete target genes, cells were transduced with respective sgRNA and selected with 5-10µg/ml blasticidin S HCl (Thermo Fisher, # A1113903) or 2-5µg/ml Puromycin Dihydrochloride (Thermo Fisher, # A1113803). The M3 sg*Con* and M3 sg*SUZ12* single clones were selected, expanded and validated by immunoblotting. Similarly, sg*Nf1* and sg*Cdkn2a* were cloned into the pLCP2B and selected by blasticidin; sg*Cdkn2b* was cloned into the LentiCRISPRv2-mCherry vector (Addgene, #99154) in SKP cells. sg*Eed* was cloned into the lentiCRISPR-v2, and SKP605 cells were selected by puromycin. Single clones were selected, expanded, and validated. M3 (sg*Con*) cells were pooled from 6 single clones; M3 (sg*SUZ12*) cells were pooled from 9 single clones. SKP605 sg*Con* cells and sg*Eed* cells were pooled from 4 single clones each. The doubling time for M3 cells is about 48 hours, while the doubling time for SKP605 is about 24 hr. The cells are typically used for experiments within 1 month (15-30 passages) of pooling single clones.

sgRNA Oligos are as following Sequence (5' to 3') and their PAM are NGG:

sg*Con* (Control): F, caccgGCTGATCTATCGCGGTCGTC, R, aaacGACGACCGCGATAGATCAGCc; Human_sg*SUZ12*_VEFS: F, caccgTCCATTTCTTGTGGACGGAG, R, aaacCTCCGTCCACAAGAAATGGAc; Mouse_sg*E*ed#1_WD40: F, accgGAAGGTTTGGGTCTCGTGGG, R, aaacCCCACGAGACCCAAACCTTCc; Mouse_sg*Eed*#2_WD40: F, caccgGGAGAAGGTTTGGGTCTCGT, R, aaacACGAGACCCAAACCTTCTCCc; Mouse_sg*Suz12*_VEFS:F, tcccGAATTTTCTGATGTGAATGA, R, aaacTCATTCACATCAGAAAATTC; Mouse_*Nf1*: F, caccgGGGAGAACTCCCTATAGCTA, R, aaacTAGCTATAGGGAGTTCTCCCc; Mouse_sg*Cdkn2a*: F, caccgCGGTGCAGATTCGAACTGCG, R, aaacCGCAGTTCGAATCTGCACCGc; Mouse_sg*Cdkn2b*: F, caccgTTGGGCGGCAGCAGTGACGC, R, aaacGCGTCACTGCTGCCGCCCAAc;

Tumor infiltrate analysis by flow cytometry

Fresh tumor tissues were minced with razor blades and then digested with 10 ml digestion buffer separately (MPNST tumors: 200 U/ml Collagenase I + 300 U/ml Collagenase IV + 4µg/ml DNase I + RPMI-1640, breast cancer tumors: 300 U/ml Collagenase III + 4µg/ml DNase I + HBSS). All these enzymes were purchased from Worthington: Collagenase I #LS004196, Collagenase III #LS004182, Collagenase IV # LS004188, and DNase I #LS002006. HBSS buffer was purchased from Thermo Fisher Scientific #14025076. Samples were incubated for 1 hour in 37°C shaker. After digestion, single cells were isolated by mashing tumors through 70µm filters, enriched with Ficoll (Thermo Fisher Scientific, #17144002). Spleens were minced directly and lysed in ACK lysis buffer (Thermo Fisher Scientific, #A1049201) to remove red blood cells. For FACS, about

1.5 million isolated cells were resuspended in 70μl FACS buffer (2% FBS + 1 mM EDTA (0.5M, 1:500) + 10 mM HEPES (1M, 1:100) in PBS) with Fc blocking for 15 minutes on ice first, followed by incubation with indicated antibodies plus Fc blocking for 30 minutes on ice, protected from light. Foxp3 / Transcription Factor Staining Buffer Kit (Tonbo, #TNB-0607-KIT) was used to stain intracellular markers. All FACS were performed on BD LSR Fortessa, and data were analyzed using FlowJo software. Antibodies for FACS: Anti-Mouse CD16 / CD32 (1:500, 2.4G2) (TONBO, #70-0161-U100), Ghost Dye™ Violet 450 (1:1000, TONBO, #13-0863-T100), PE-Texas Red_CD45 (1:200, Thermo Fisher Scientific, #MCD4517), APC/Cy7_TCR β (1:200, BioLegend, #109220), PerCP-Cyanine5.5_ CD8a (1:200, TONBO, #65-1886-U100), BV510_CD4 (1:200, BD, #563106), PE_B220 (1:400, TONBO, #50-0452-U100), FITC_F4/80 (1:200, TONBO, #35-4801-U100), APC-Cyanine7_CD11b (1:200, TONBO, #25-0112-U100), PE-Cyanine7_CD11c (1:200, TONBO, #60-0114-U025), APC_MHC Class II (I-A/I-E) (1:600, TONBO, #20-5321-U100), FITC_IFN gamma (1:100, TONBO, #35-7311-U100), PE-Cyanine7_TNF alpha (1:100, Thermo Fisher Scientific, #25-7423-82).

T-cell stimulation

Cell Stimulation Cocktail (plus protein transport inhibitors) (500X) (Thermo Fisher, #00-4975-93) was diluted in T cell medium (RPMI 1640 + 10%FBS + L-glutamine (2 mM) + penicillin (100 U/ml + streptomycin (100μg/ml) + 55μM 2-Mercaptoethanol (Thermo Fisher, #21985023)). 2 million cells were suspended in 200μl T-cell medium with stimulation cocktail in plates, then incubated for 4 hours at 37°C incubator. After incubation, cells were harvested, stained with surface markers and then intracellular markers for FACS analysis.

Protein extraction and immunoblotting

After washed by precooling PBS, cells were lysed using preheated 2% SDS followed by boiling for 30 min at 95°. Protein concentrations were determined using BCA protein assay kit (Thermo Fisher, #23225). Protein samples were prepared by adding LDS loading buffer (4X) (Thermo Fisher, #NP0008) and 1 M DTT followed by boiling for 30 min at 95°. Proteins were separated by 4-12% Bis-Tris Gel (Thermo Fisher, #NP0336BOX) with MES SDS running buffer (20X) (Thermo Fisher, #NP0002), and transferred to the nitrocellulose membrane (BioRad, #1620115). Membranes were blocked with blocking buffer (Thermo Fisher, #UH289384) for 1 hour at room temperature and incubated with the indicated primary antibodies at 4° overnight. After washing with 1xTris-buffered saline Tween-20 (TBST) (25 mM Tris, 150 mM NaCl, 2 mM KCl, pH 7.4, supplemented with 0.2% Tween-20) three times for 30 minutes, membranes were incubated with peroxidase-conjugated secondary antibodies at room temperature for 1 hour. The membranes were washed again with TBST three times and visualized with chemiluminescence using HRP substrate (Millipore, #WBKLS0500) or Pico (Thermo Fisher, #34578).

RNA isolation and qRT-PCR

The sequences of primers used for qPCR analysis were listed as follows (5'--3').

Human_*RPL27*: F, CATGGGCAAGAAGAAGATCG, R TCCAAGGGGATATCCACAGA; Human_*SUZ12*: F, TTGCAGCTTACGTTTACTGGTT, R, GGAACTTGCCTTATTGGACAACT; Human_*EED*: F, CTGTAGGAAGCAACAGAGTTACC, R, CATAGGTCCATGCACAAGTGT; Human_*CD274*: F, TGGCATTTGCTGAACGCATTT, R, TGCAGCCAGGTCTAATTGTTTT;

Human_*IRF1*: F, CTGTGCGAGTGTACCGGATG, R, ATCCCCACATGACTTCCTCTT; Human_*CCL2*: F, CAGCCAGATGCAATCAATGCC , R, TGGAATCCTGAACCCACTTCT; Human_*CD74*: F, GATGACCAGCGCGACCTTATC, R, GTGACTGTCAGTTTGTCCAGC; Human_*CIITA*: F, CCTGGAGCTTCTTAACAGCGA, R, TGTGTCGGGTTCTGAGTAGAG; Human_*HLA-DRA*: F, AGTCCCTGTGCTAGGATTTTTCA, R, ACATAAACTCGCCTGATTGGTC; Human_*HLA-DRB*: F, CGGGGTTGGTGAGAGCTTC, R, AACCACCTGACTTCAATGCTG; Human_*LAP3*: F, GTCTGGCCGTGAGACGTTT , R, ACCATAAAAGGTTCGAGTCTTCC; Mouse_*Rpl27*: F, AAAGCCGTCATCGTGAAGAAC, R, GATAGCGGTCAATTCCAGCCA; Mouse_*Eed*: F, AGCCACCCTCTATTAGCAGTT, R, GCCACAAGAGTGTCTGTTTGGA; Mouse_*Ccl2*: F, TAAAAACCTGGATCGGAACCAAA, R, GCATTAGCTTCAGATTTACGGGT; Mouse_*Cxcl10*: F, CCAAGTGCTGCCGTCATTTTC, R, TCCCTATGGCCCTCATTCTCA; Mouse_*Il2*: F, AACCTGAAACTCCCCAGGAT, R, TCATCGAATTGGCACTCAAA; Mouse_*Oas1*: F, GGGCCTCTAAAGGGGTCAAG, R, TCAAACTTCACTCCACAACGTC; Mouse_*Cd274*: F, AGTATGGCAGCAACGTCACG, R, TCCTTTTCCCAGTACACCACTA; Mouse_*Irf1*: F, GGCCGATACAAAGCAGGAGAA, R, GGAGTTCATGGCACAACGGA; Mouse_*Actb*: F, GTGACGTTGACATCCGTAAAGA, R, GCCGGACTCATCGTACTCC; Mouse_*Ifnb1*: F, TGGAGATGACGGAGAAGATG; R, TTGGATGGCAAAGGCAGT; Mouse_*Ifna4*: F, CCTGTGTGATGCAGGAACC, R, TCACCTCCCAGGCACAGA; Mouse_*Il6*: F, AGGCATAACGCACTAGGTTT, R, AGCTGGAGTCACAGAAGGAG.

Cell colony formation assay

AT3 cells were seeded into 6-well plates at a concentration of 200 cells per well. After 9 days. colonies were fixed with fixation solution (10% methanol + 10% acetic acid) at room temperature for 15 minutes and then stained with a solution of 1% crystal violet in methanol for 15 minutes.

Assay for transposase-accessible chromatin using sequencing (ATAC-seq) and analysis

For data analysis, ATAC-seq reads were quality and adapter trimmed using Trim Galore before aligning to human genome assembly hg19 with Bowtie2 using the default parameters. Aligned reads with the same start position and orientation were collapsed to a single read before subsequent analysis. Density profiles were created using the BED Tools suite, with subsequent normalization to a sequencing depth of ten million reads for each library. To ascertain regions of open chromatin, MACS2 (https://github.com/taoliu/MACS) was used with a p-value setting of 0.001 against a cell line-matched input sample. A global peak atlas was created by first removing blacklisted regions then merging all peaks within 500 bp and counting reads with version 1.6.1 of feature counts (http://subread.sourceforge.net). Differential enrichment was scored using DESeq2 for all pairwise group contrasts. Differential peaks were then merged into a union set, and k-means clustering was performed from k=4:10, stopping when redundant clusters emerged. Peak-gene associations were created by assigning all intragenic peaks to that gene, and otherwise using linear genomic distance to transcription start site. GSEA (http://software.broadinstitute.org/gsea) was performed with the pre-ranked option and default parameters, where each gene was assigned the single peak with the largest (in magnitude) log2 fold change associated with it. Motif signatures were obtained using HOMER v4.5 (http://homer.ucsd.edu).

Tumor-intrinsic PRC2 inactivation drives a context-dependent immune-desert microenvironment and is sensitized by immunogenic viruses

Juan Yan and Yuedan Chen *et al*.

(Date: 07/07/2022)

Corresponding authors: Dr. Yu Chen, Phone: 646-888-3356, Email: cheny1@mskcc.org. Dr. Ping Chi, Phone: 646-888-3338, Email: chip@mskcc.org. Address: Memorial Sloan Kettering Cancer Center, 1275 York Avenue, New York, NY 10065.

The PDF file includes "Full unedited gel for Figures"

Full unedited gel for Figure 3A

Full unedited gel for Figure 3A

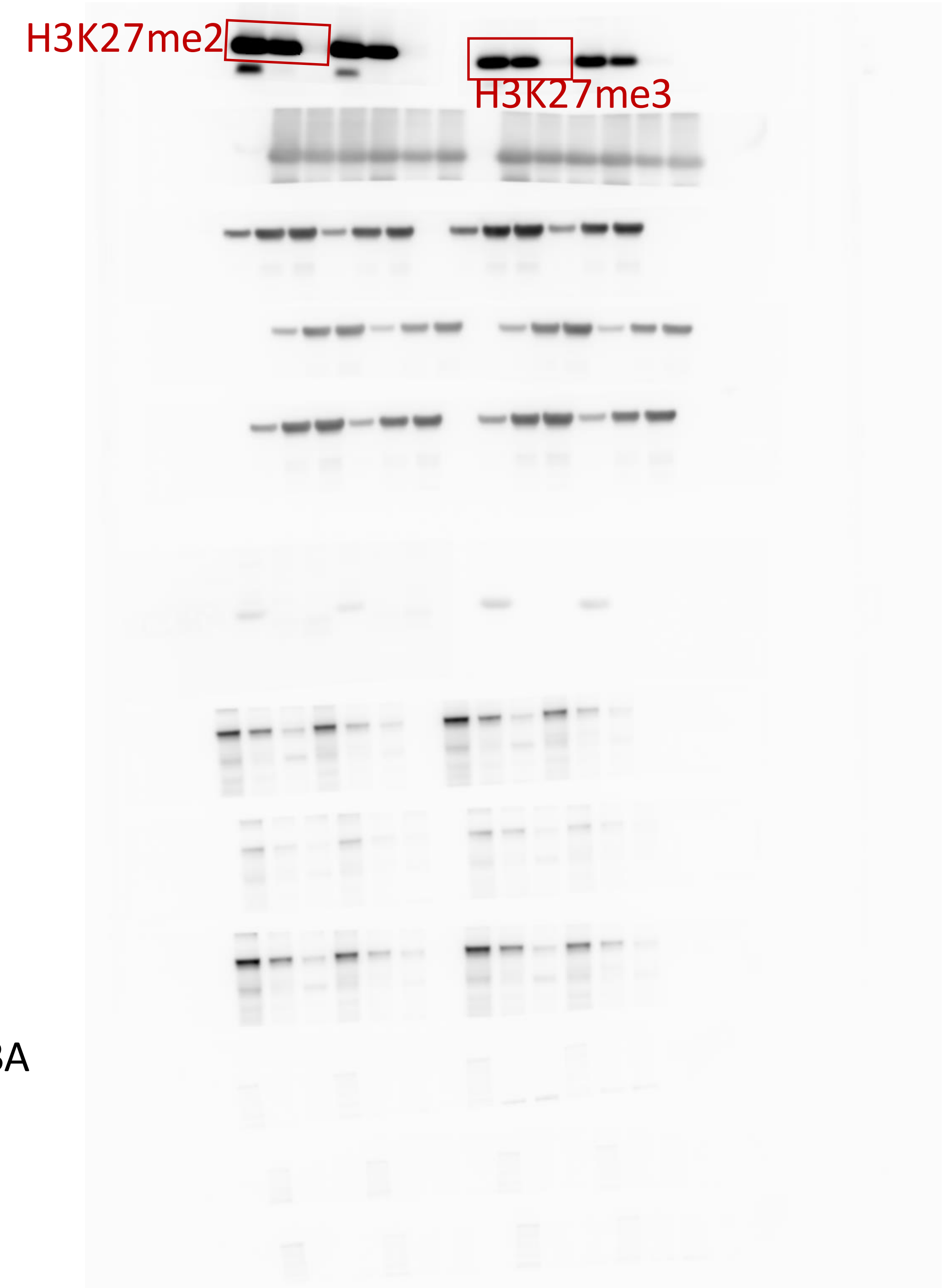

Full_unedited_gel_for_Figure_3A
_20191105_1106_1.tif

Full unedited gel for Figure 3A

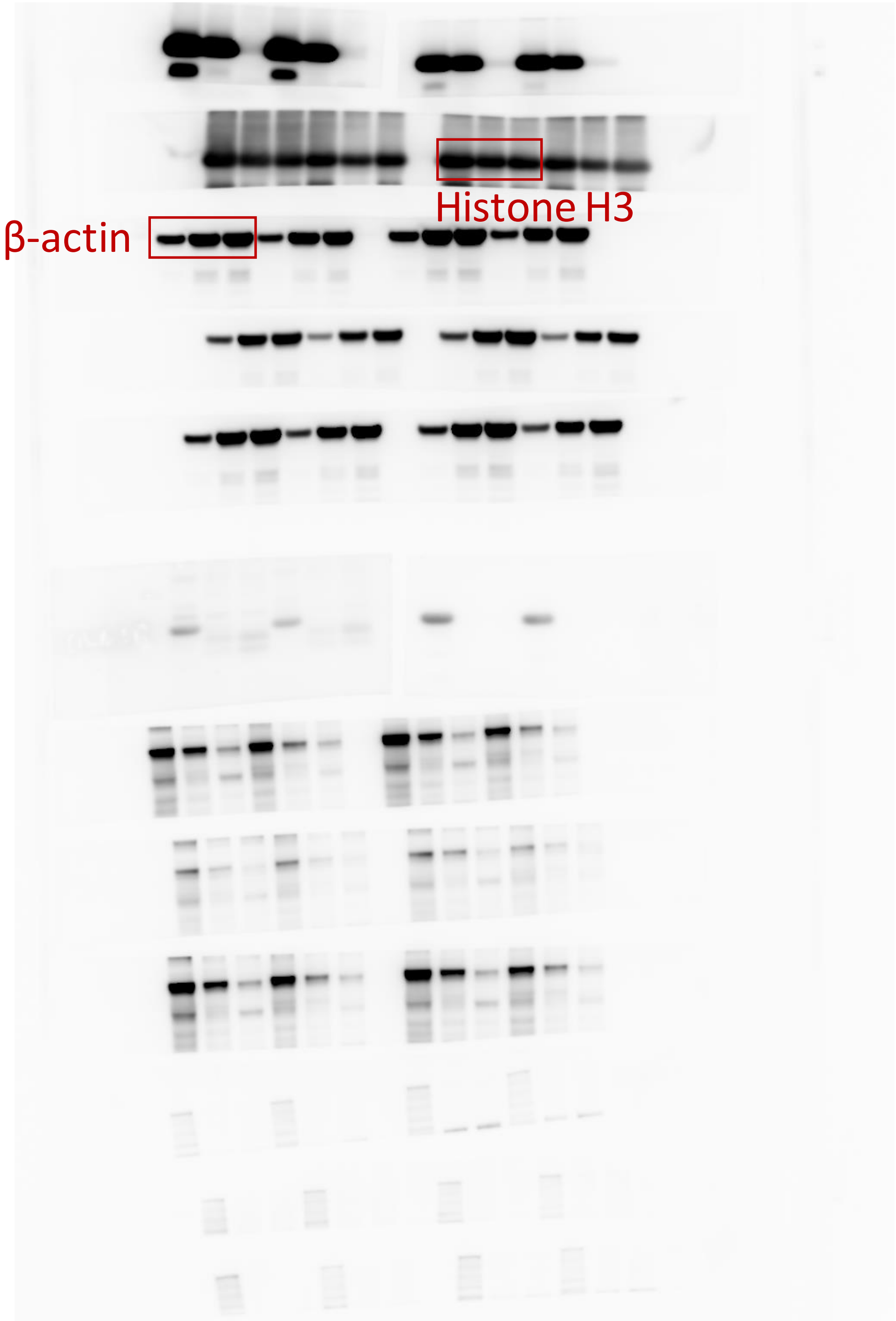

Full_unedited_gel_for_Figure_3A
_20191105_1106_3.tif

Full unedited gel for Figure 3A

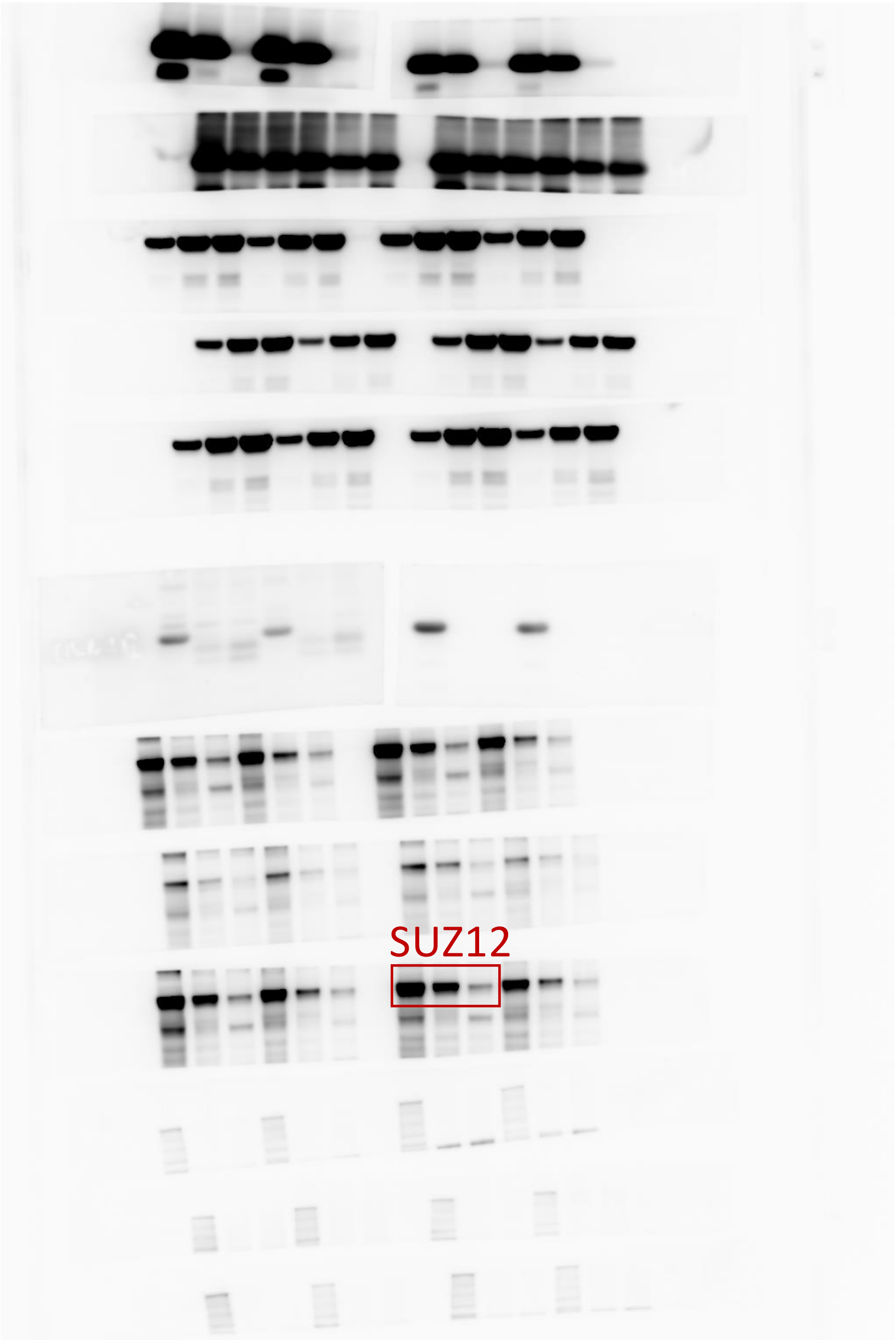

Full_unedited_gel_for_Figure_3A _20191105_1106_5.tif

Full unedited gel for Figure 3A

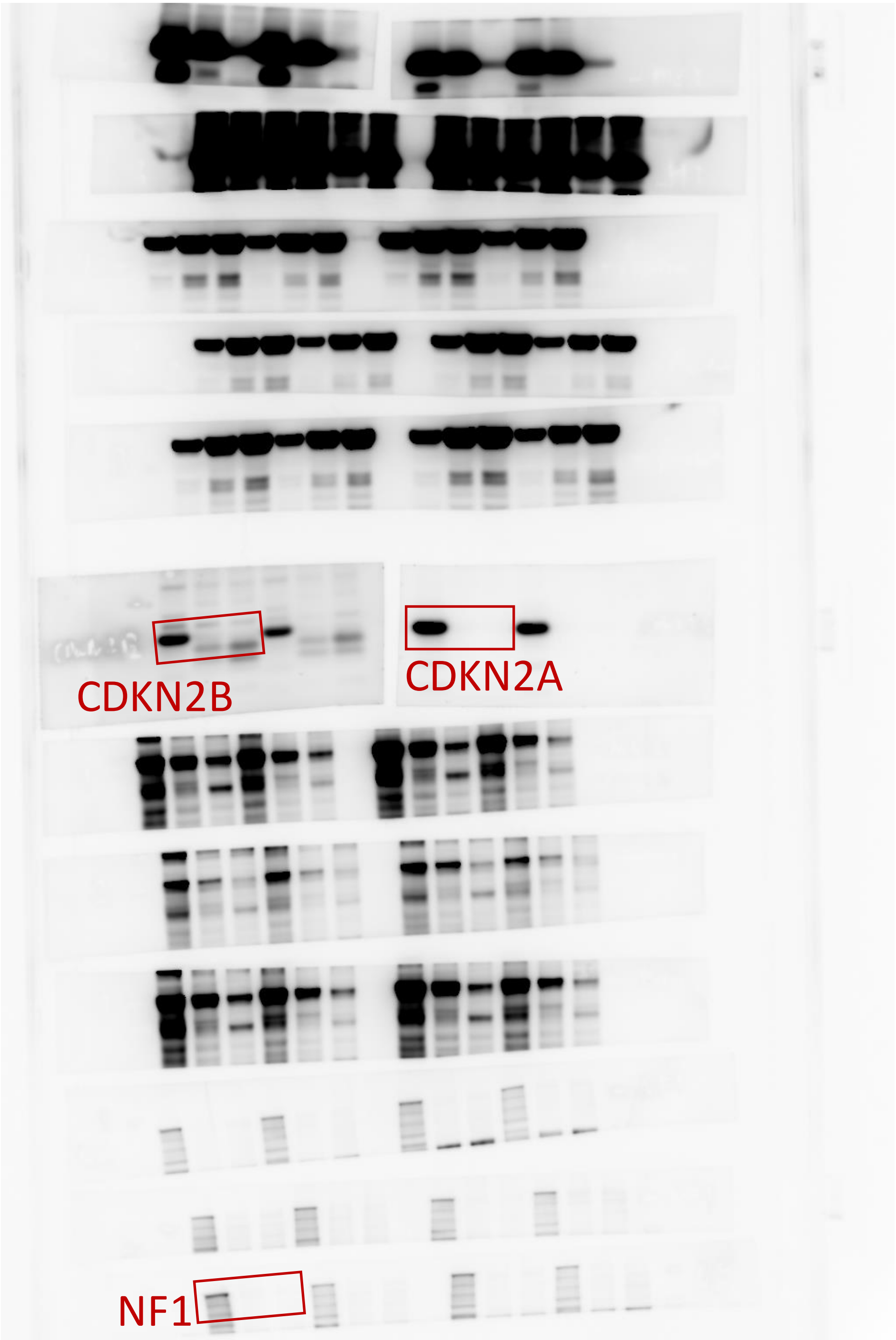

Full_unedited_gel_for_Figure_3A
_20191105_1106_15.tif

Full unedited gel for Figure 3A

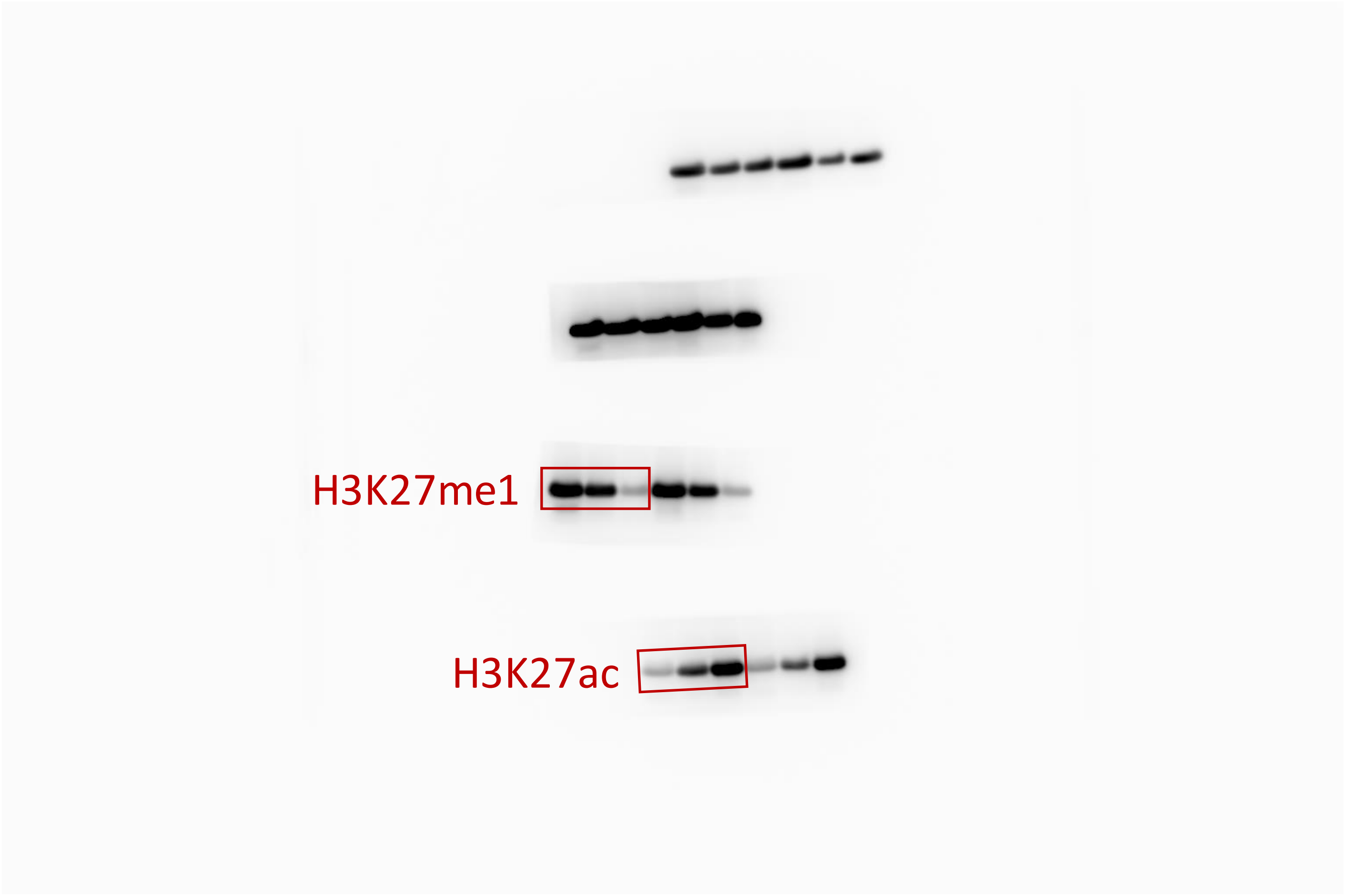

Full_unedited_gel_for_Figure_3A_20200131_1727_2.tif

Full unedited gel for Figure 5A

Full unedited gel for Figure 5A

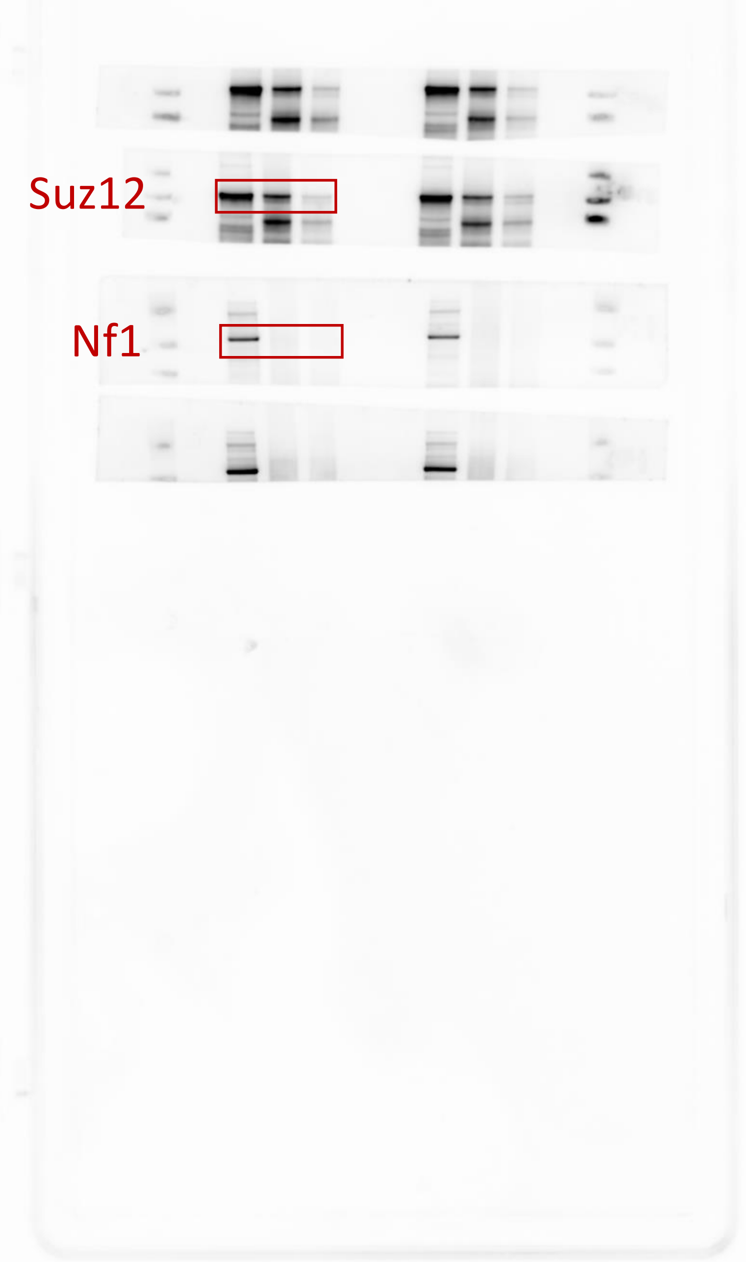

Full_unedited_gel_for_Figure_5A_
20200811_164933-11.tif

Full unedited gel for Figure 5A

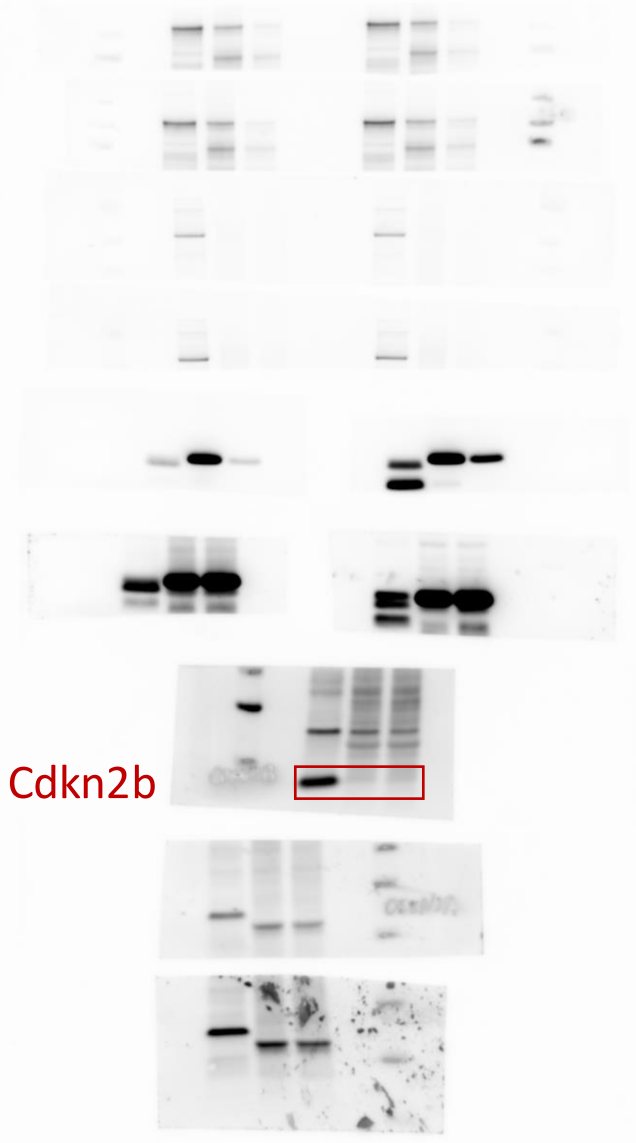

Full_unedited_gel_for_Figure_5A_
20200811_163106-13.tif

Full unedited gel for Figure 5A

Full_unedited_gel_for_Figure_5A_20200811_163106-15.tif

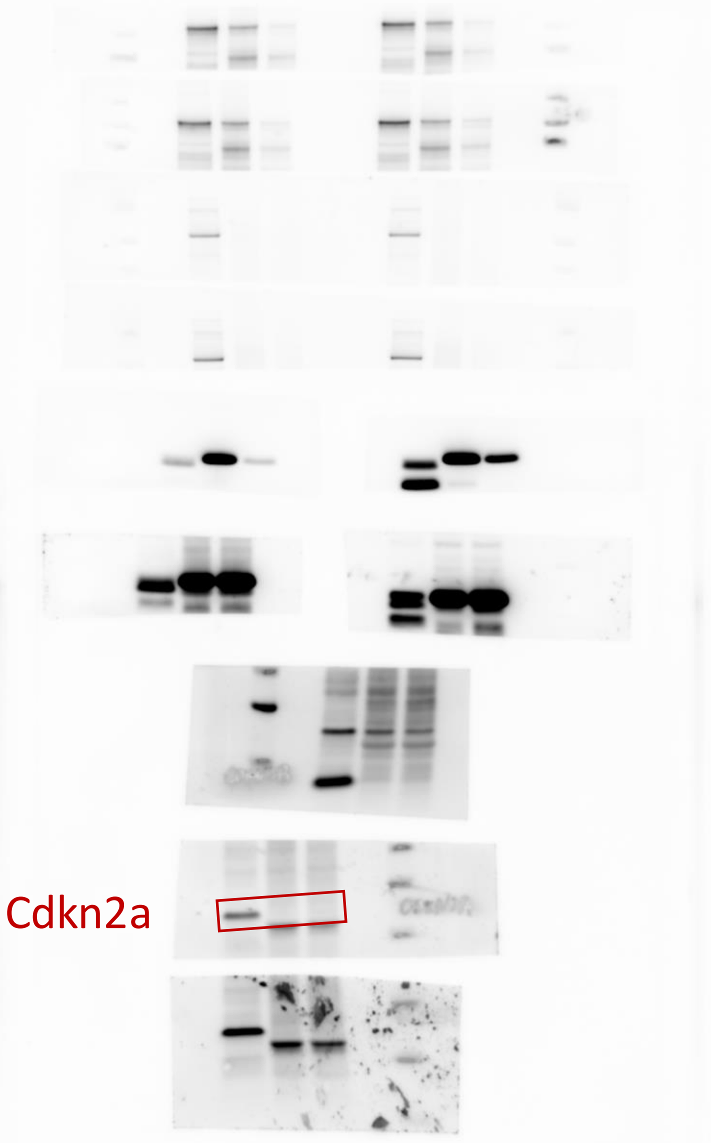

Full unedited gel for Figure 5A

Full_unedited_gel_for_Figure_5A_
20200811_164101-06.tif

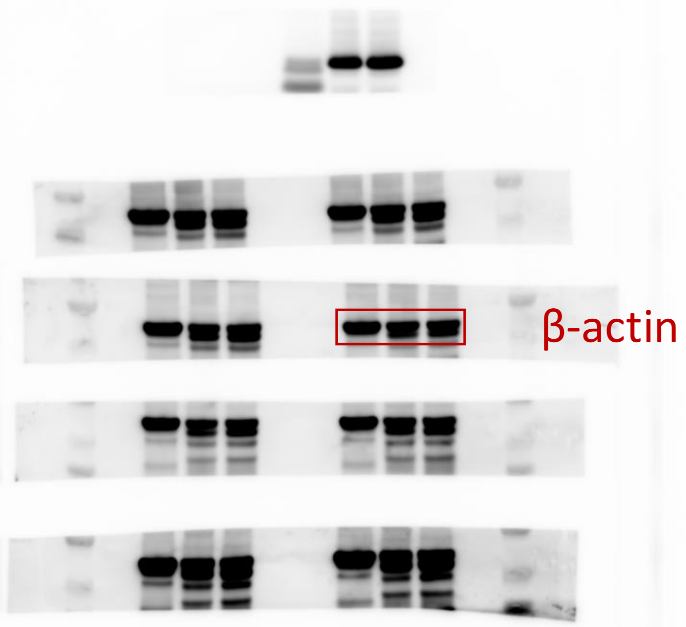

Full unedited gel for Figure 5A

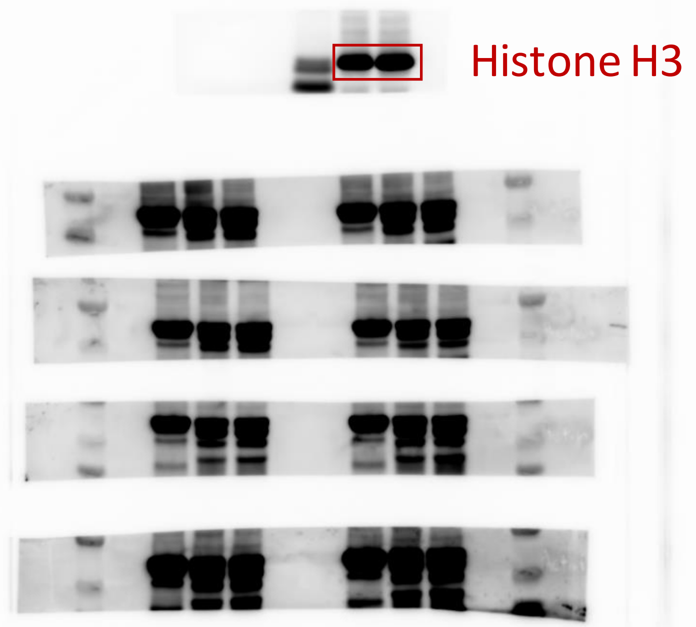

Full_unedited_gel_for_Figure_5A_
20200811_164101-12.tif

Full unedited gel for Figure 5A

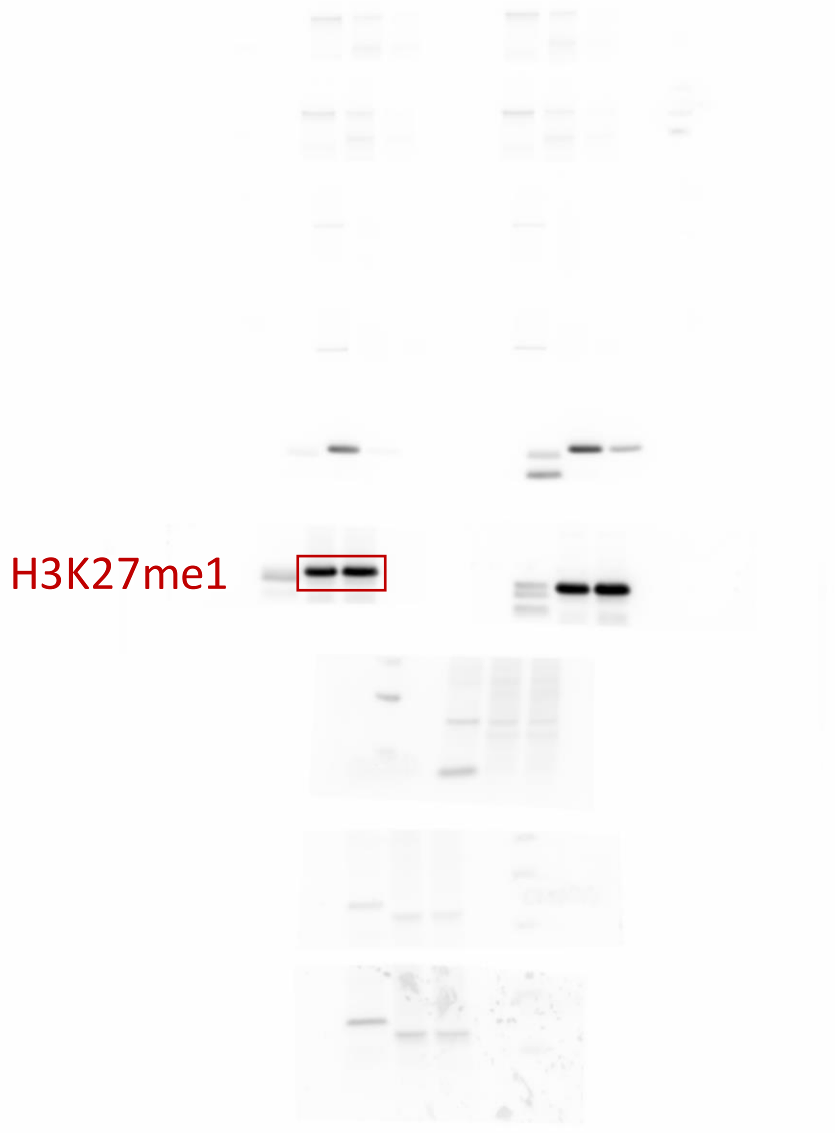

Full_unedited_gel_for_Figure_5A_
20200811_163106-02.tif

Full unedited gel for Figure 5A

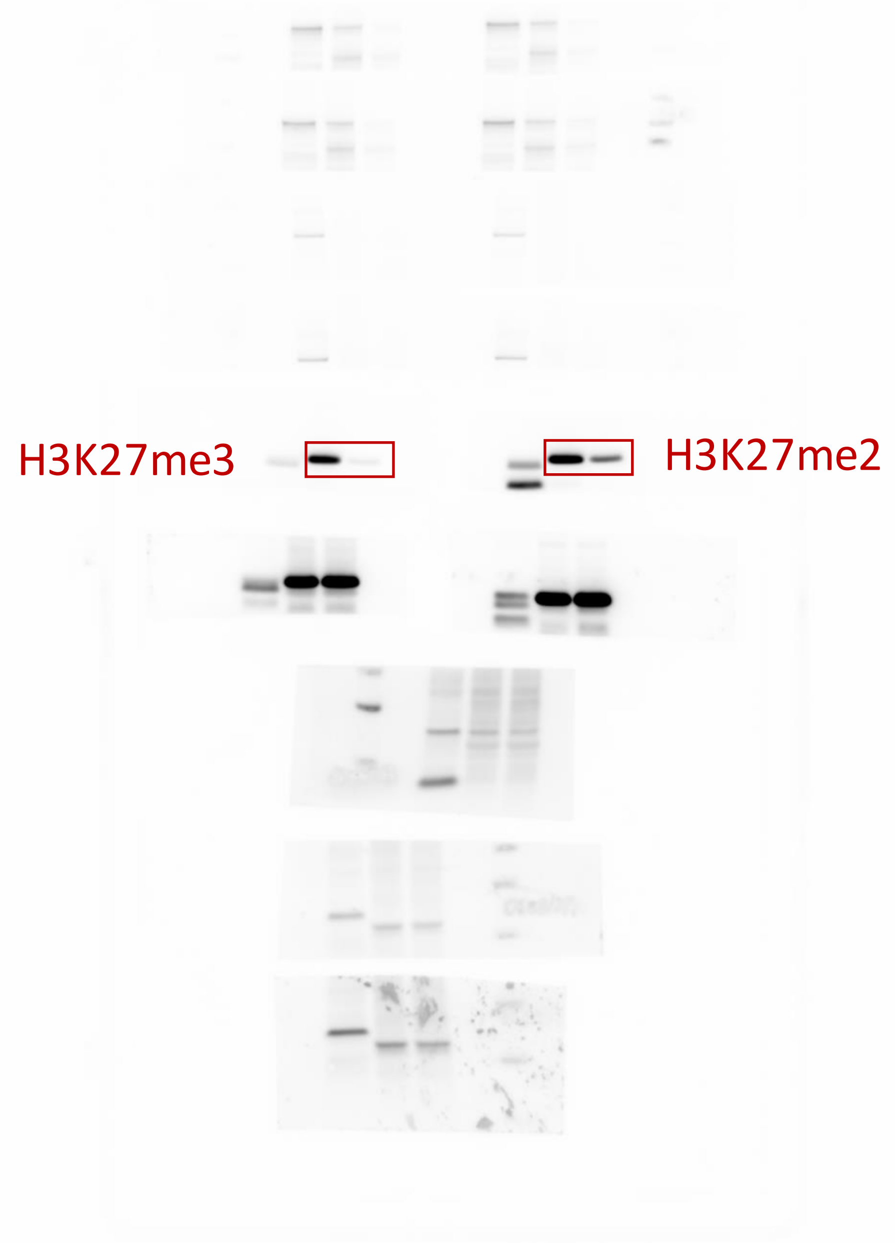

Full_unedited_gel_for_Figure_5A_
20200811_163106-05.tif

Full unedited gel for Figure 5A

H3K27ac

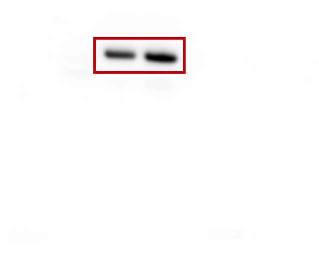

Full_unedited_gel_for_Figure_5A_
20200820_132918-02.tif

Full unedited gel for Figure 5D

Full unedited gel for Figure 5D

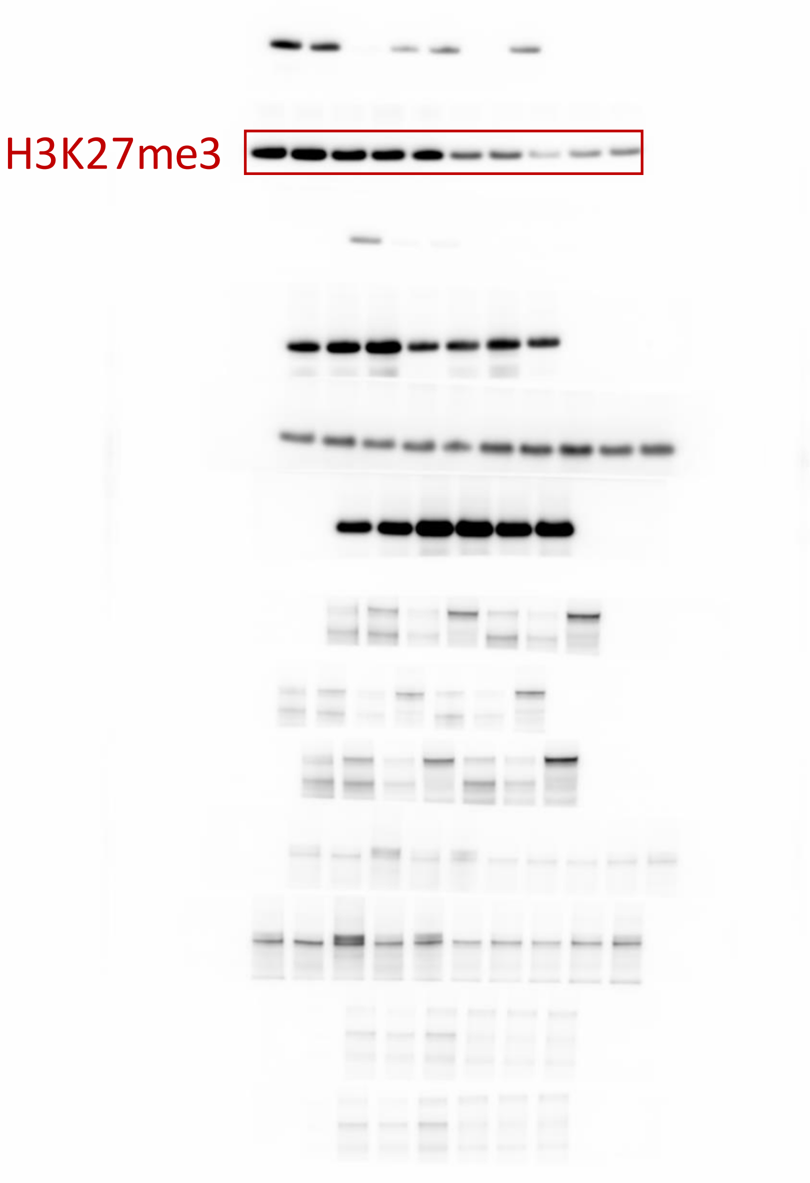

Full_unedited_gel_for_Figure_5D_
20200806_125955-03.tif

Full unedited gel for Figure 5D

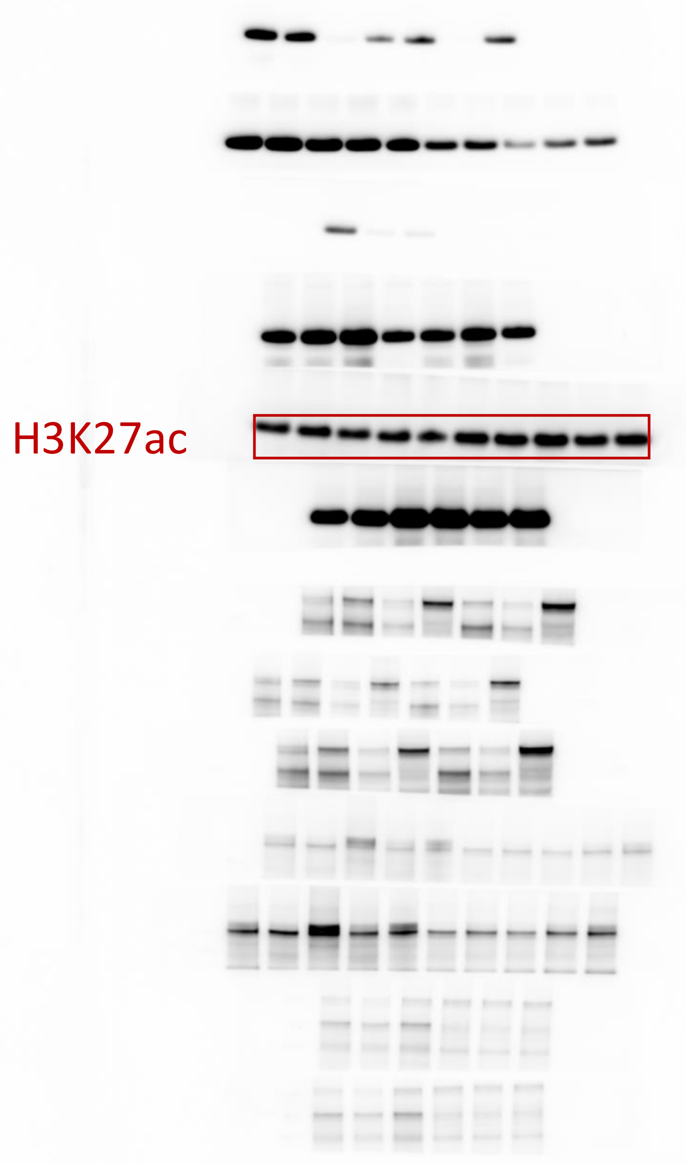

Full_unedited_gel_for_Figure_5D_
20200806_125955-06.tif

Full unedited gel for Figure 5D

Full_unedited_gel_for_Figure_5D_
20200806_125955-11.tif

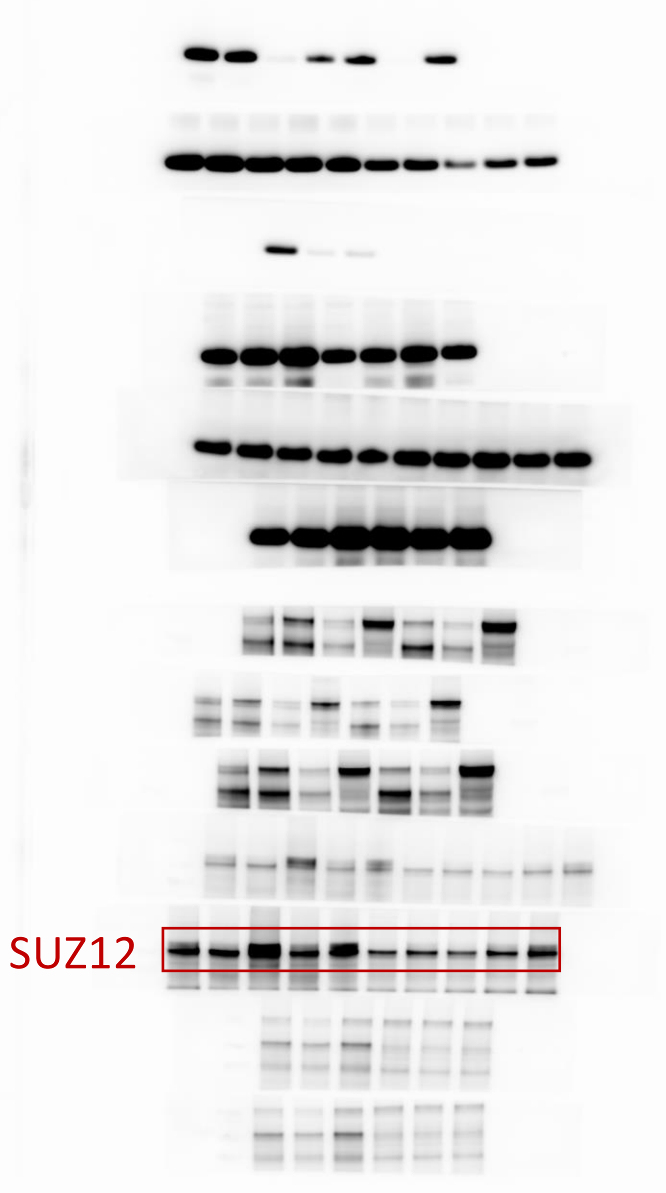

Full unedited gel for Figure 5D

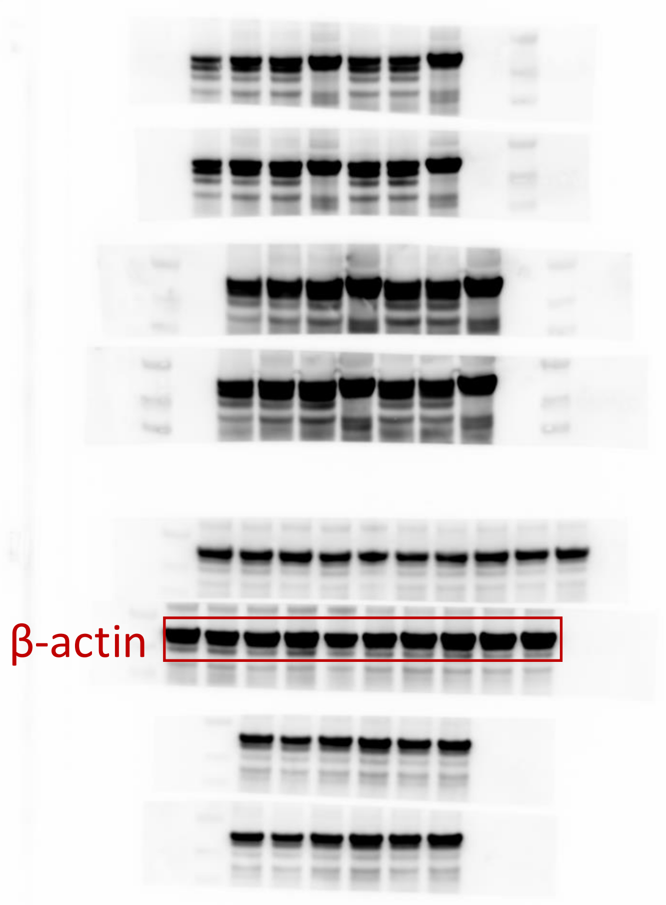

Full_unedited_gel_for_Figure_5D_
20200806_124839-04.tif

Full unedited gel for Figure 6C and Supplementary Figure 6L

Full unedited gel for Figure 6C and Supplementary Figure 6M

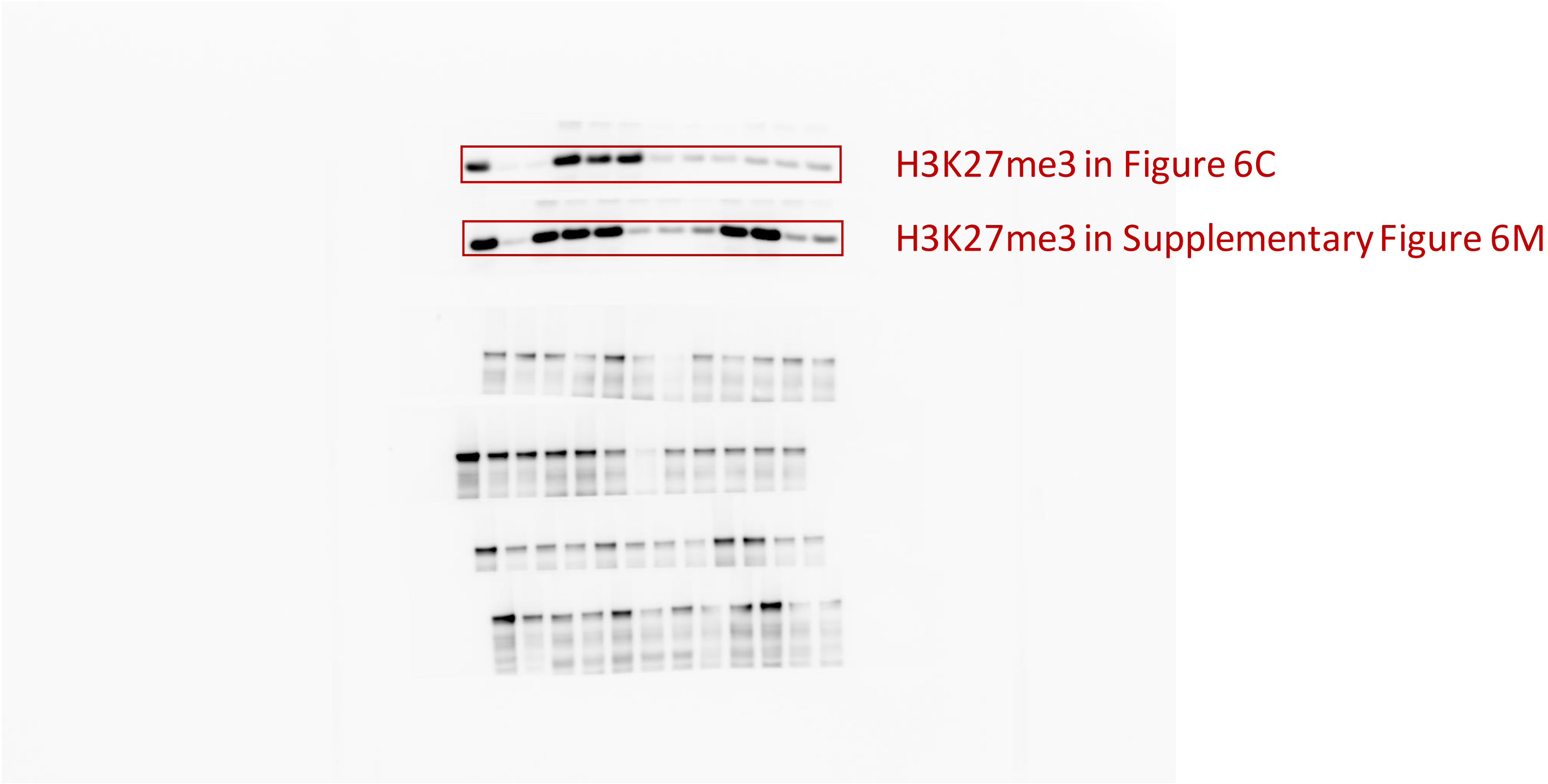

Full_unedited_gel_for_Figure_6C_and_S6M_20191220_1432_14.tif

Full unedited gel for Figure 6C and Supplementary Figure 6M

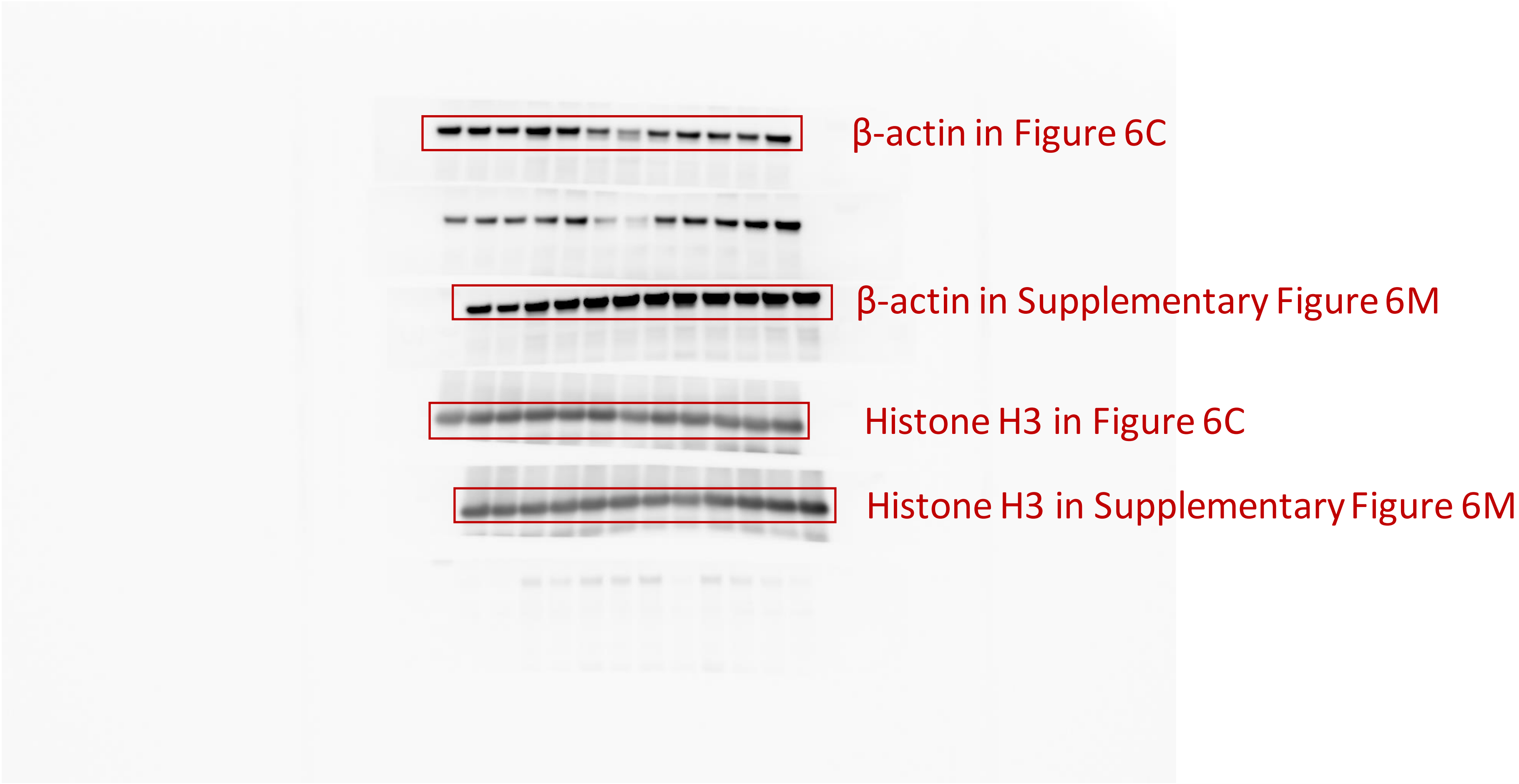

Full_unedited_gel_for_Figure_6C_and_S6M_20191220_1442_16.tif

Full unedited gel for Supplementary Figure 2B

Full unedited gel for Supplementary Figure 2B

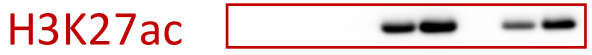

Full_unedited_gel_for_Figure_S2B
_20211008_111446-02.tif

Full unedited gel for Supplementary Figure 2B

H3K27me3

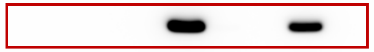

Full_unedited_gel_for_Figure_S2B
_20211008_112003-03.tif

Full unedited gel for Supplementary Figure 2B

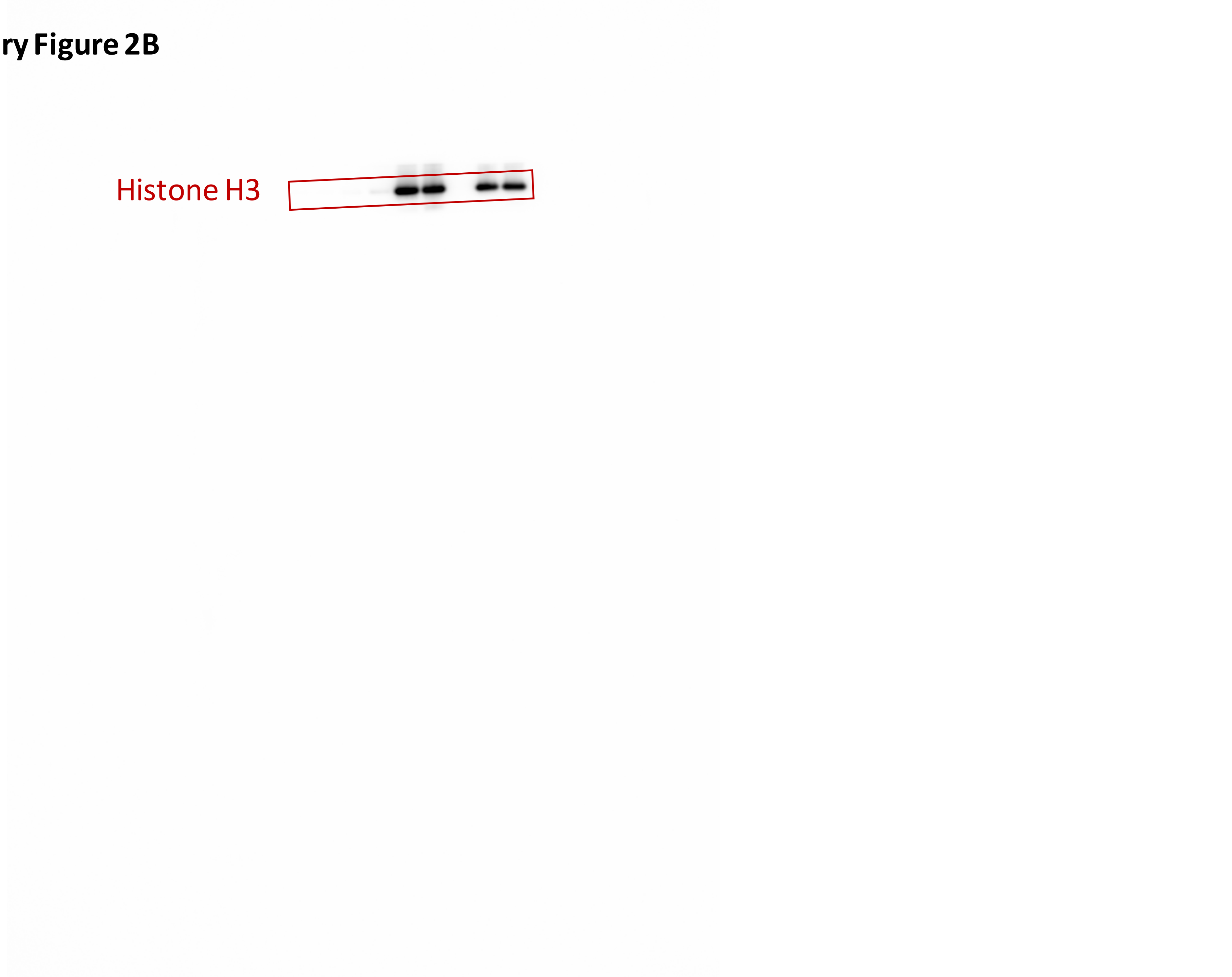

Full_unedited_gel_for_Figure_S2B
_20211008_105429-04.tif

Full unedited gel for Supplementary Figure 2B

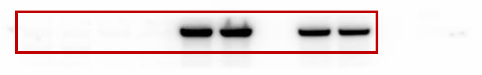

Full_unedited_gel_for_Figure_S2B
_20211010_131111-02.tif

Full unedited gel for Supplementary Figure 2B

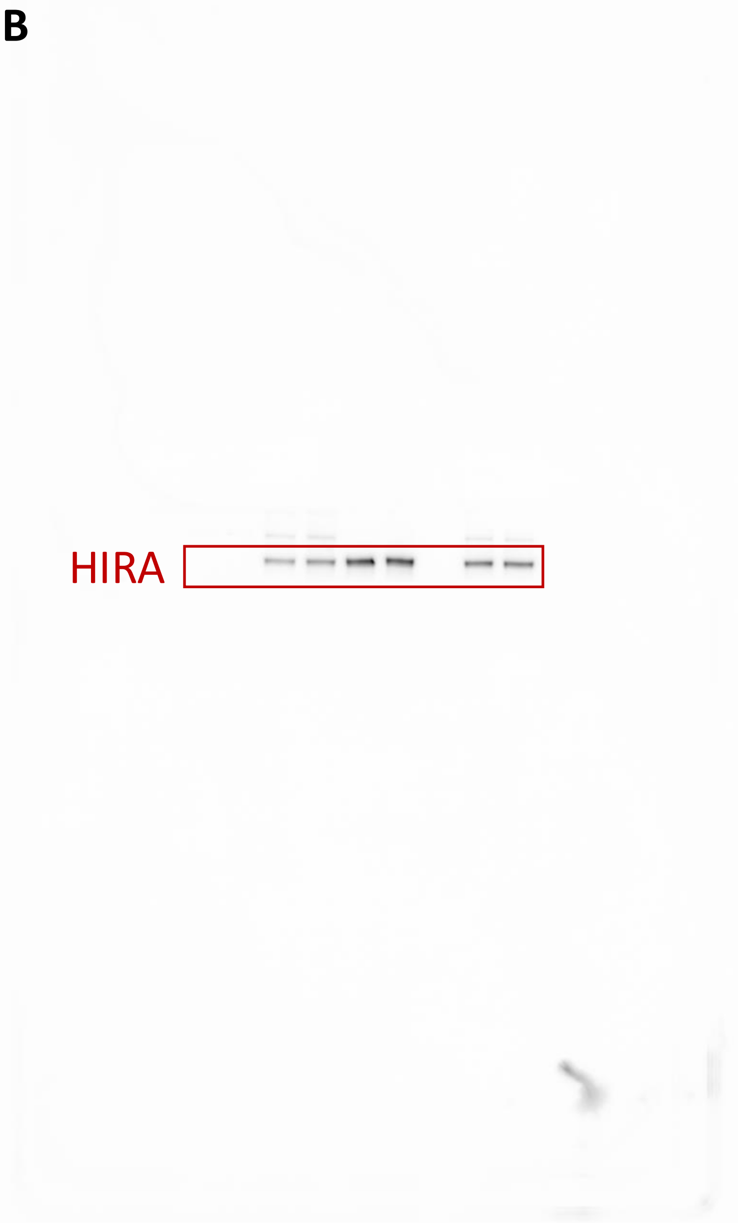

Full_unedited_gel_for_Figure_S2B
_20211004_114739-12.tif

Full unedited gel for Supplementary Figure 2B

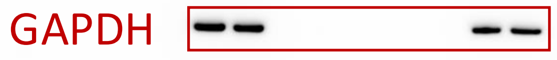

Full_unedited_gel_for_Figure_S2B
_20211008_110424-05.tif

Full unedited gel for Supplementary Figure 3A

Full unedited gel for Supplementary Figure 3A

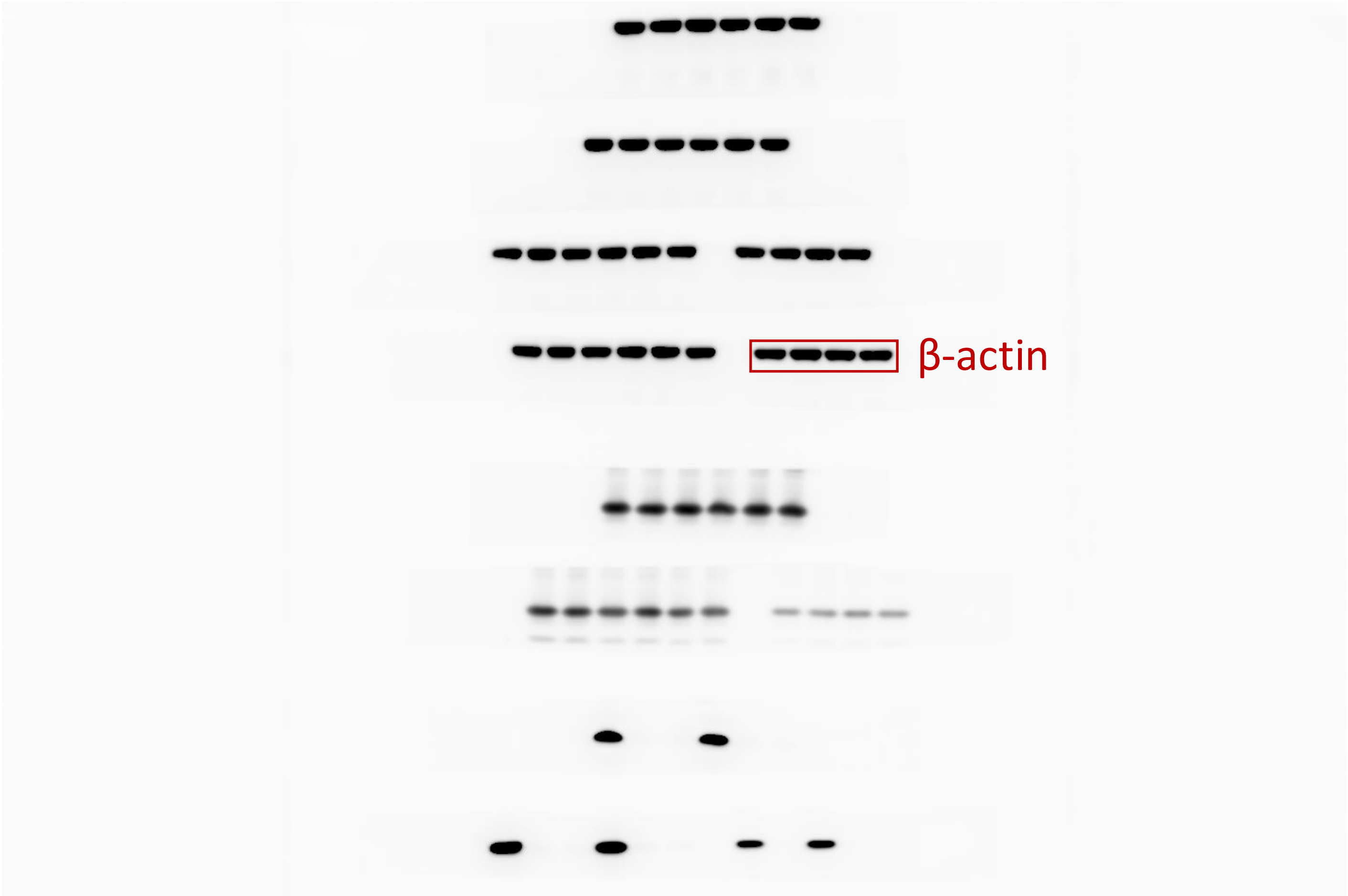

Full_unedited_gel_for_Figure_S3A_20181209_1443_3.tif

Full unedited gel for Supplementary Figure 3A

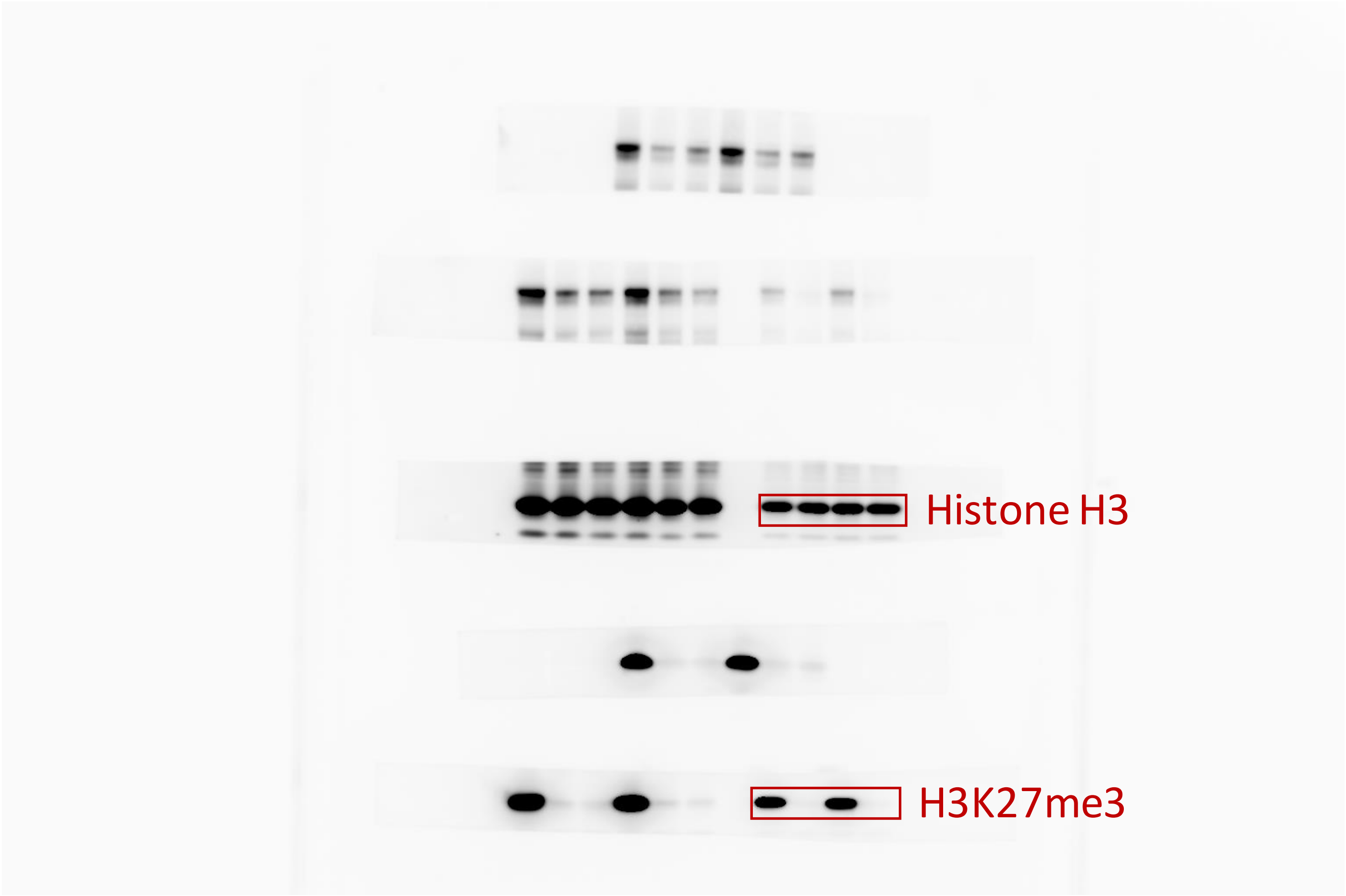

Full_unedited_gel_for_Figure_S3A_20181209_1453_7.tif

Full unedited gel for Supplementary Figure 3A

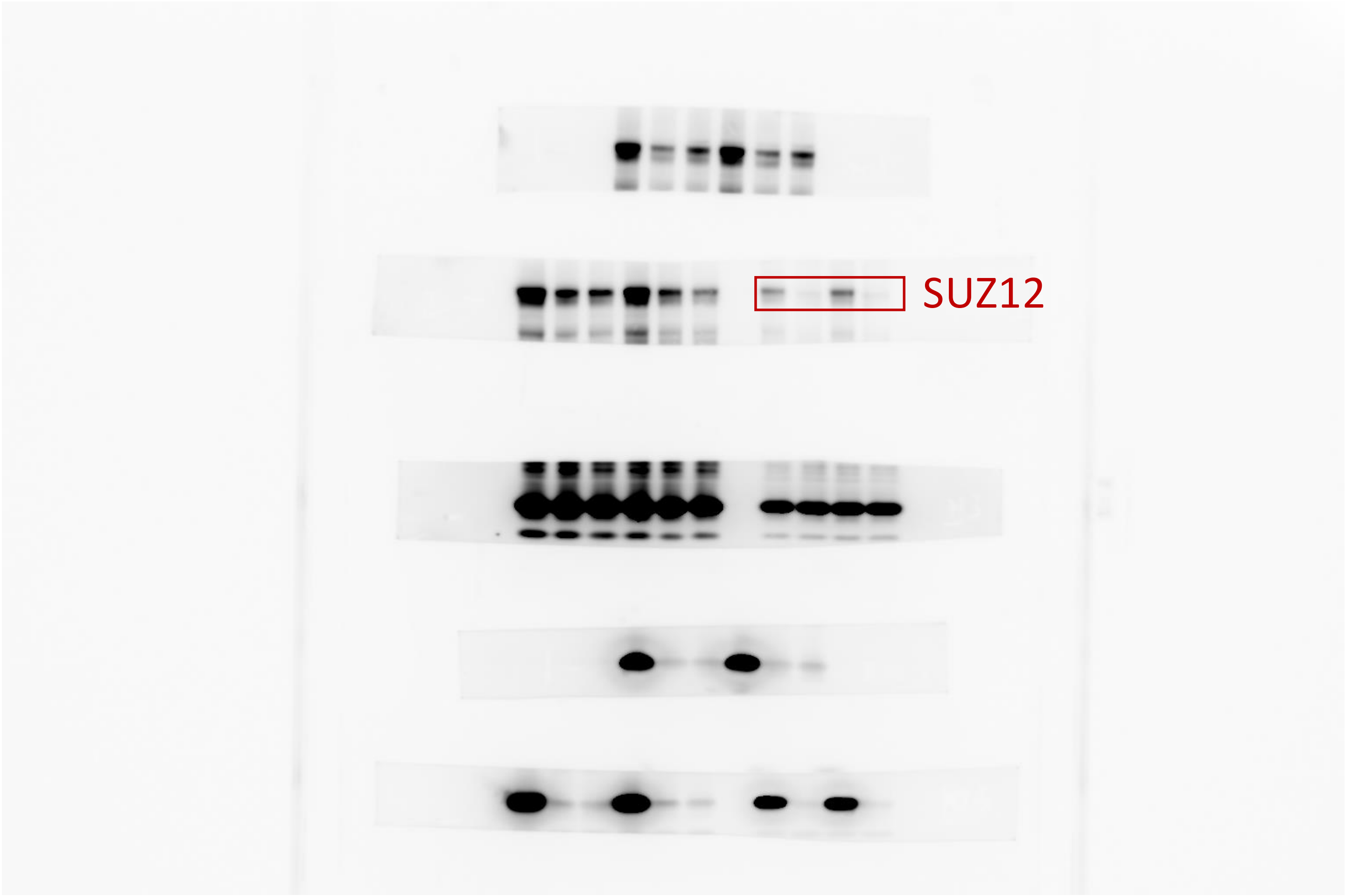

Full_unedited_gel_for_Figure_S3A_20181209_1453_15.tif

Full unedited gel for Supplementary Figure 3D

Full unedited gel for Supplementary Figure 3D

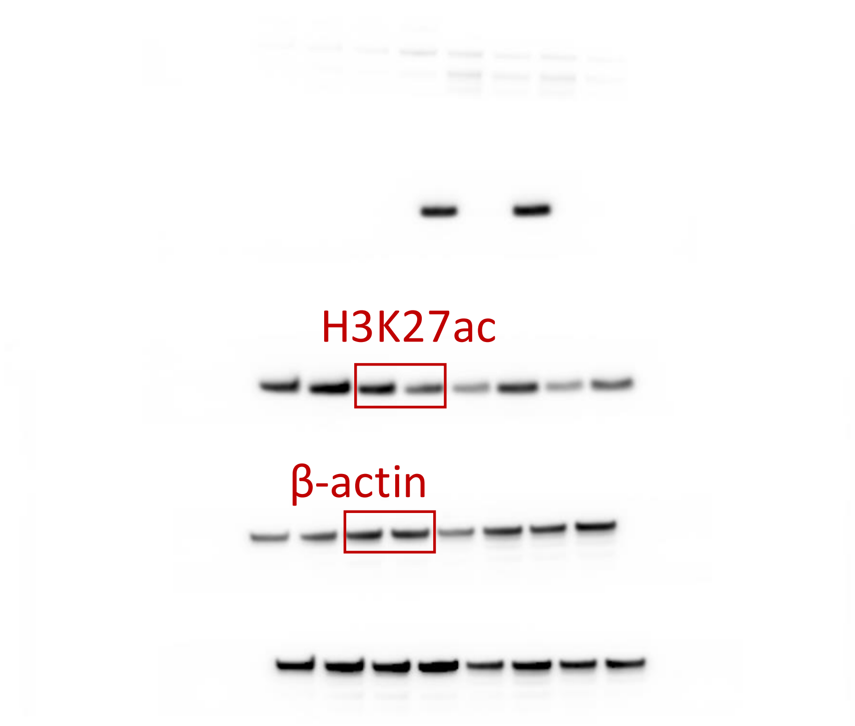

Full_unedited_gel_for_Figure_S3D_
20200717_123242-01.tif

Full unedited gel for Supplementary Figure 3D

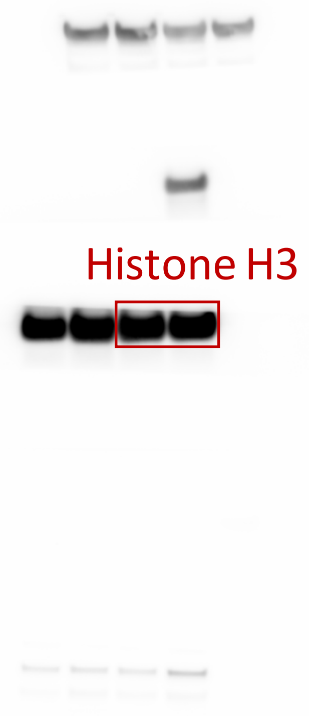

Full_unedited_gel_for_Figure_S3D_
20200719_151924-02.tif

Full unedited gel for Supplementary Figure 3D

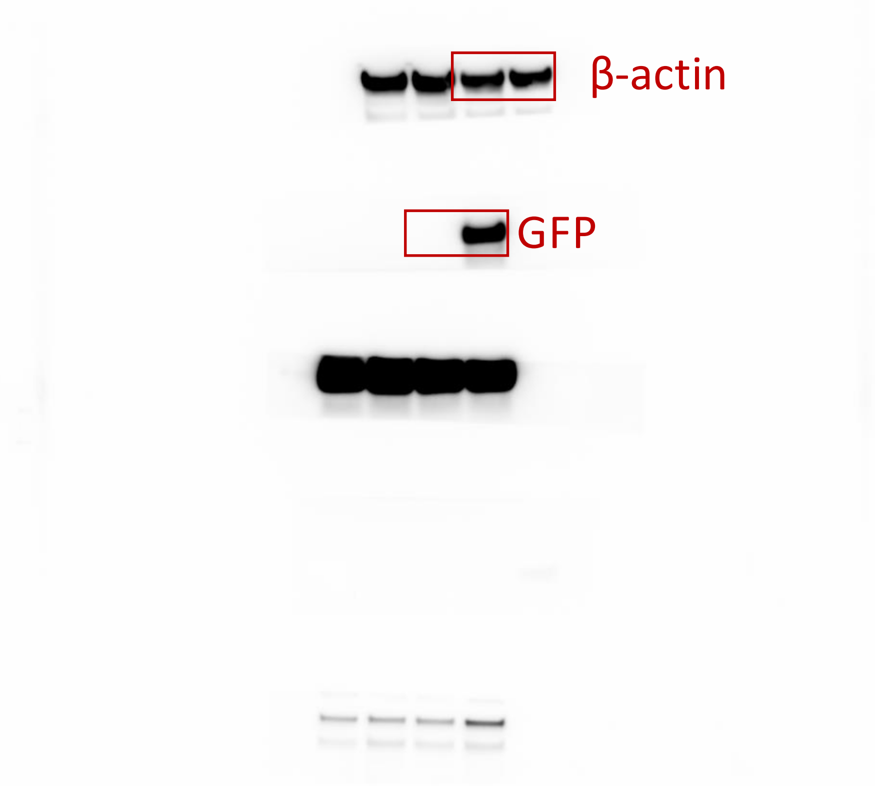

Full_unedited_gel_for_Figure_S3D_
20200719_151924-07.tif

Full unedited gel for Supplementary Figure 3D

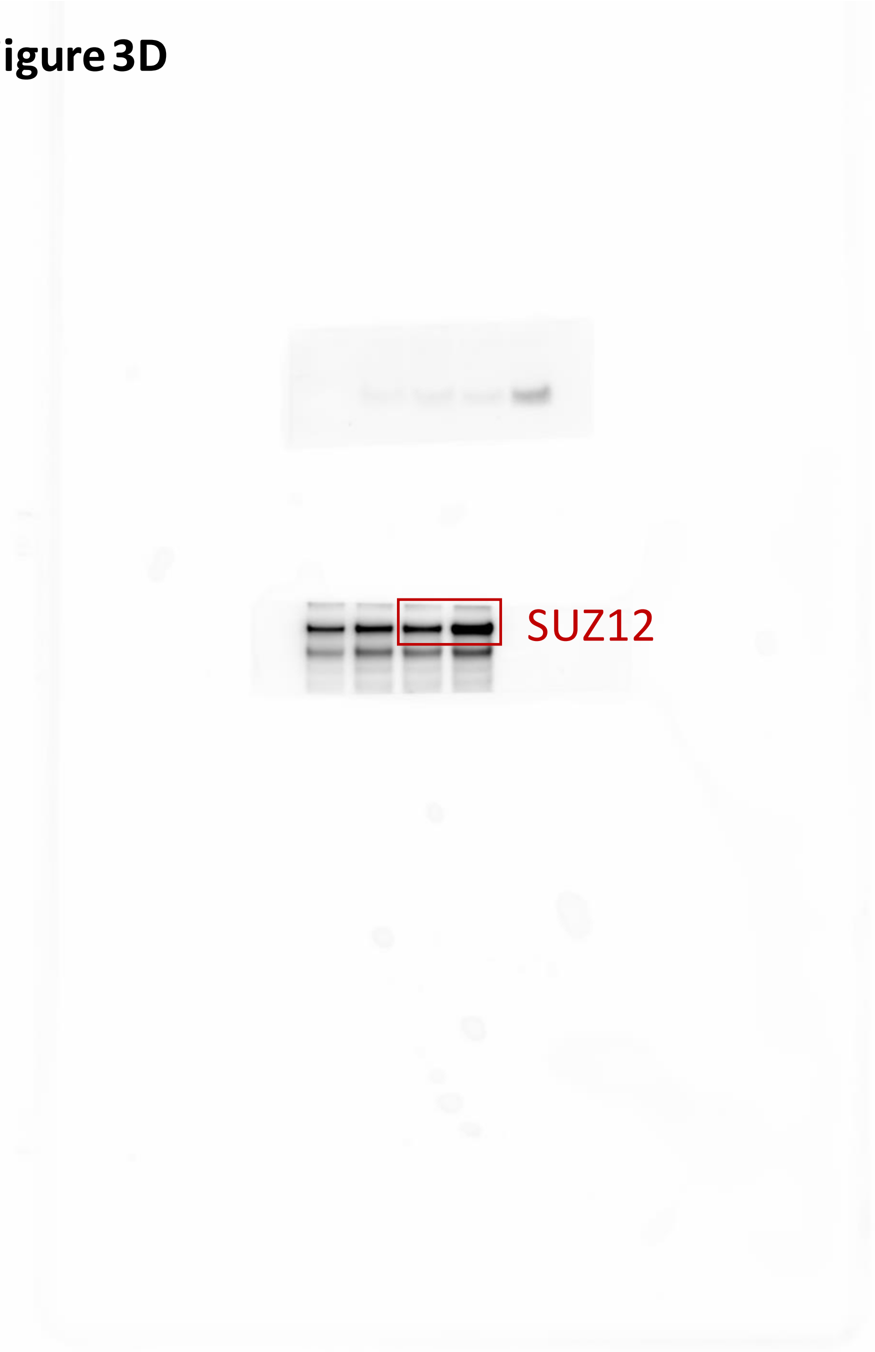

Full_unedited_gel_for_Figure_S3D_
20200719_153351-03.tif

Full unedited gel for Supplementary Figure 3D

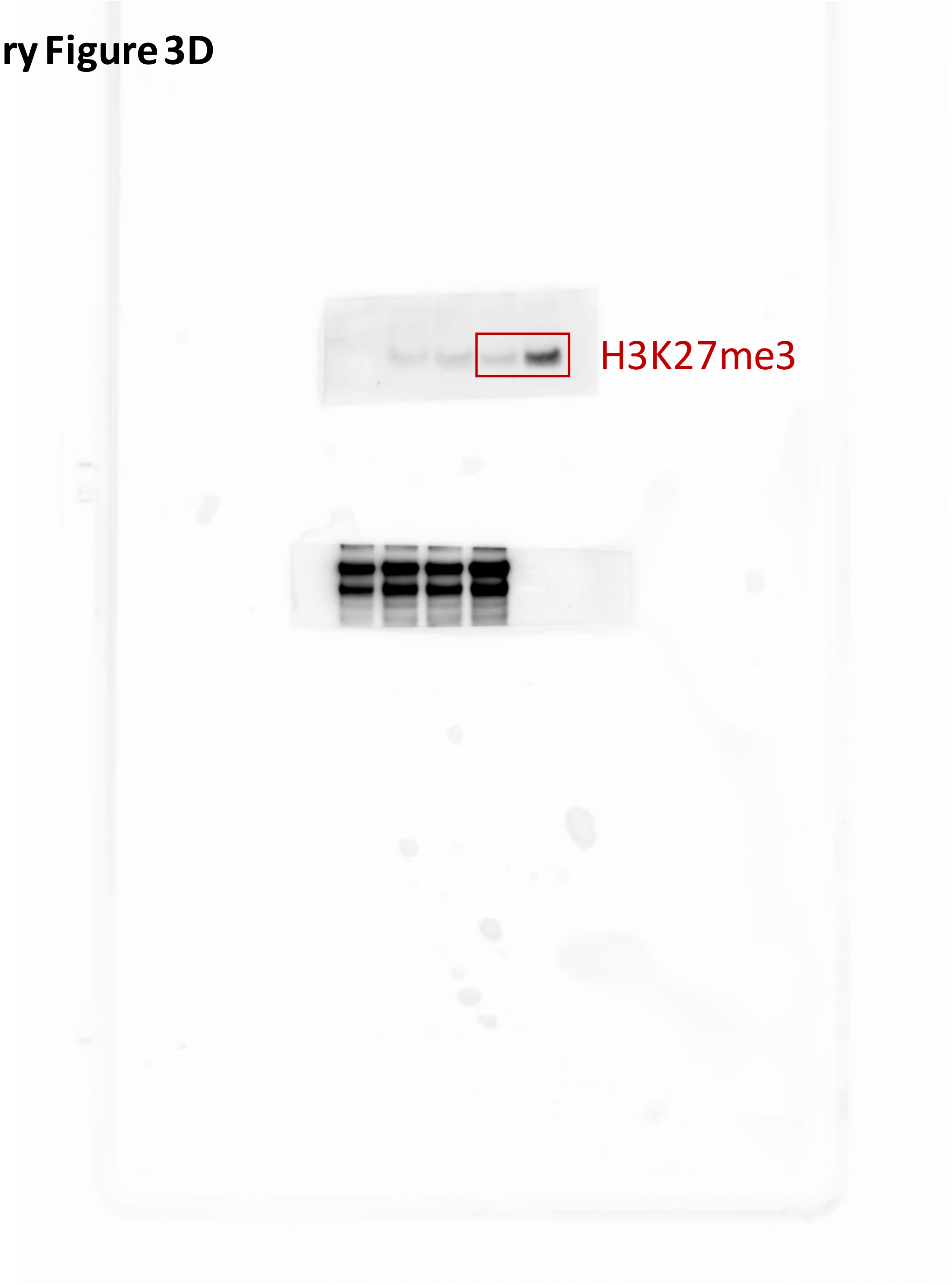

Full_unedited_gel_for_Figure_S3D_
20200719_153351-10.tif

Full unedited gel for Supplementary Figure 3D

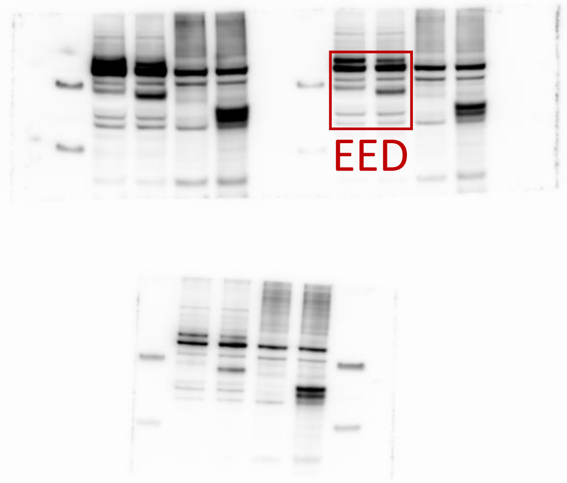

Full_unedited_gel_for_Figure_S3D_
20211010_133217-14.tif

Full unedited gel for Supplementary Figure 3D

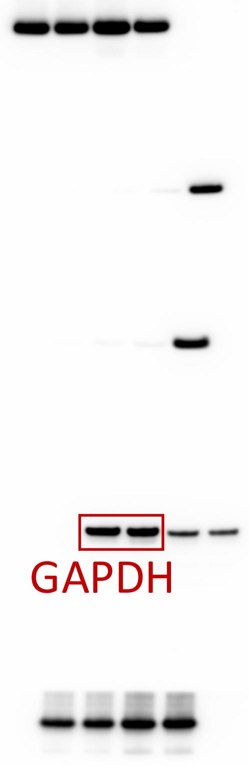

Full_unedited_gel_for_Figure_S3D_
20211010_132058-03.tif

Full unedited gel for Supplementary Figure 6D

Full unedited gel for Supplementary Figure 6D

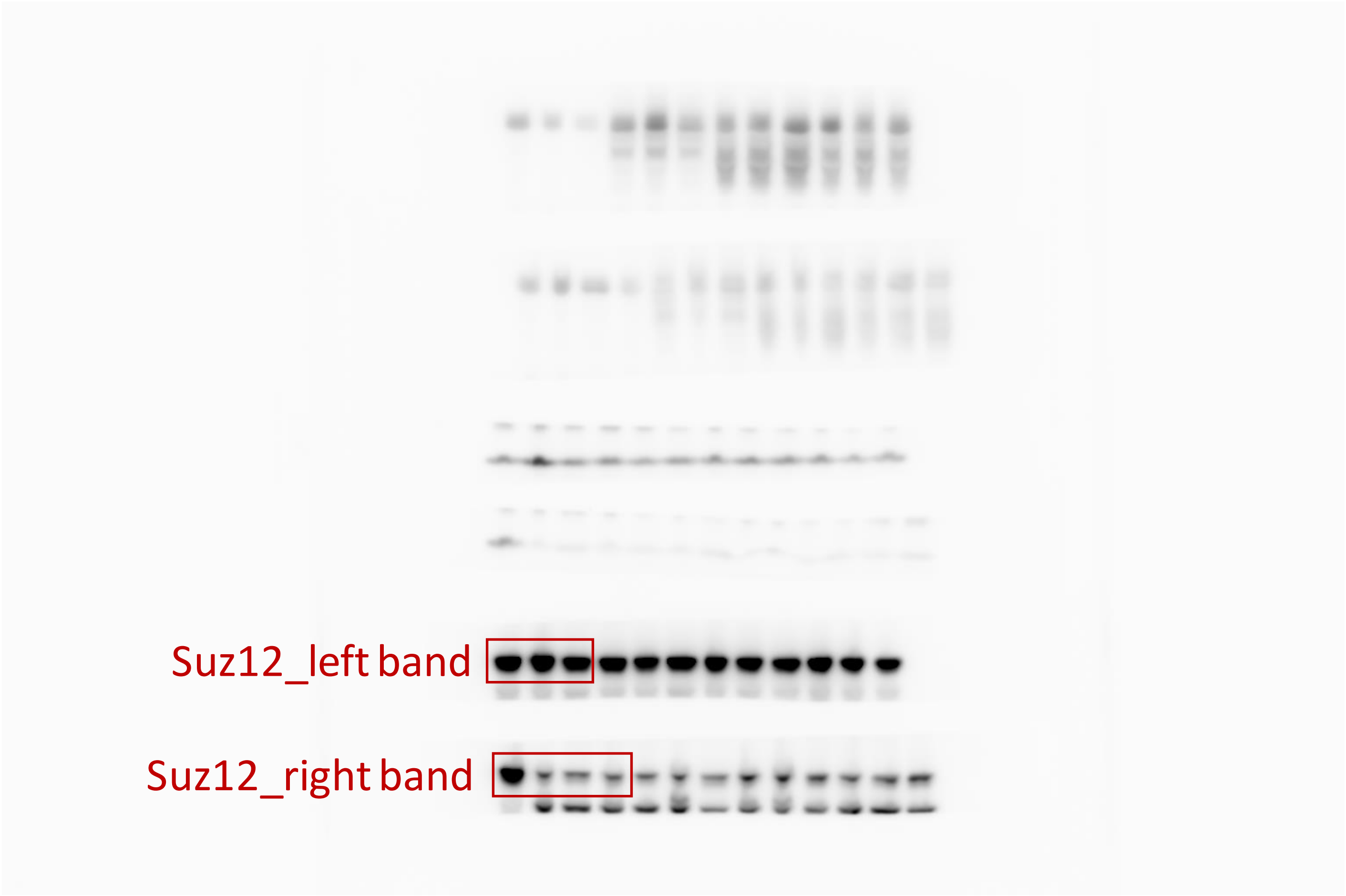

Full_unedited_gel_for_Figure_S6D_20190429_1115_2.tif

Full unedited gel for Supplementary Figure 6D

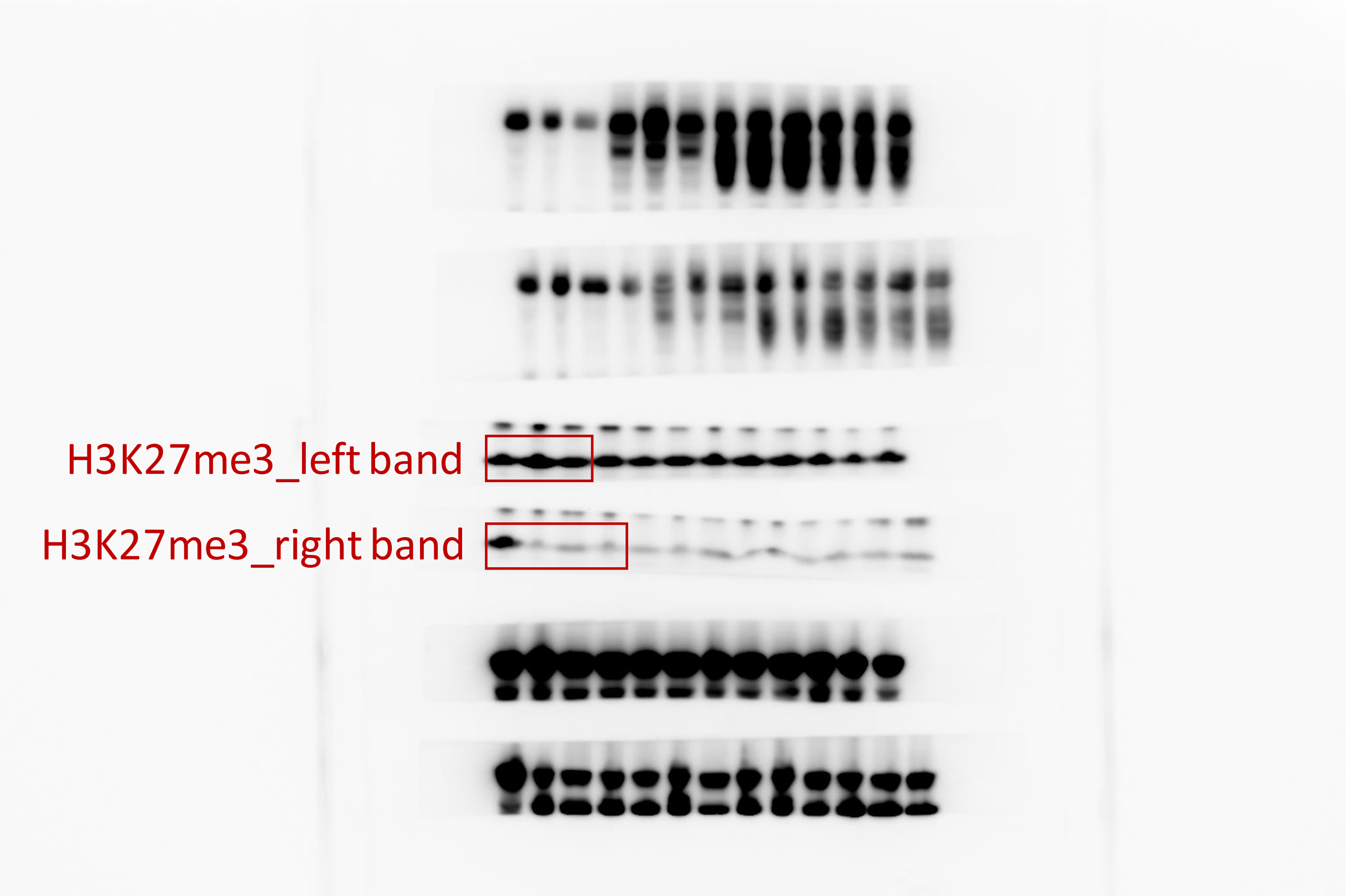

Full_unedited_gel_for_Figure_S6D_20190429_1115_13.tif

Full unedited gel for Supplementary Figure 6D

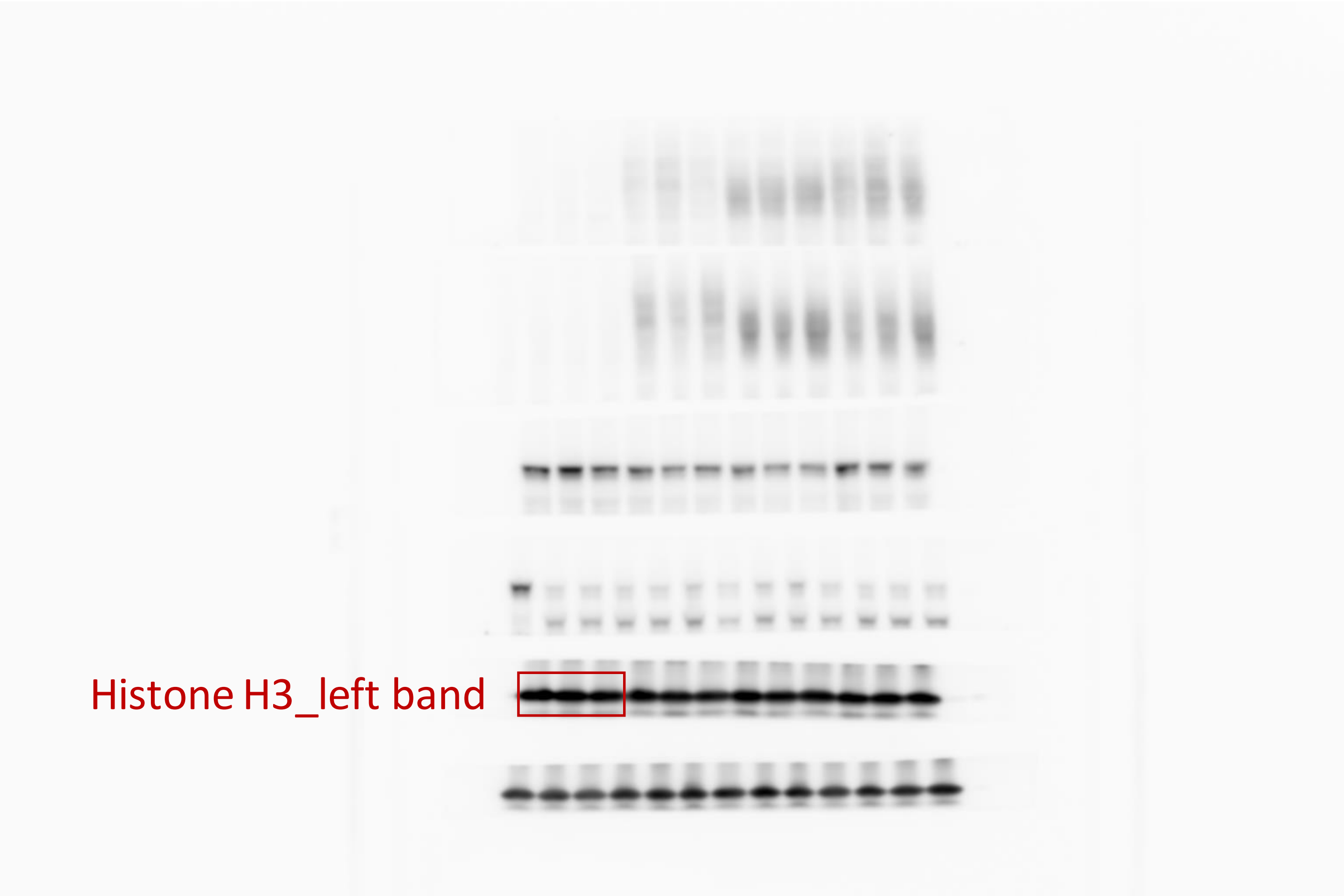

Full_unedited_gel_for_Figure_S6D_20190501_1353_6.tif

Full unedited gel for Supplementary Figure 6D

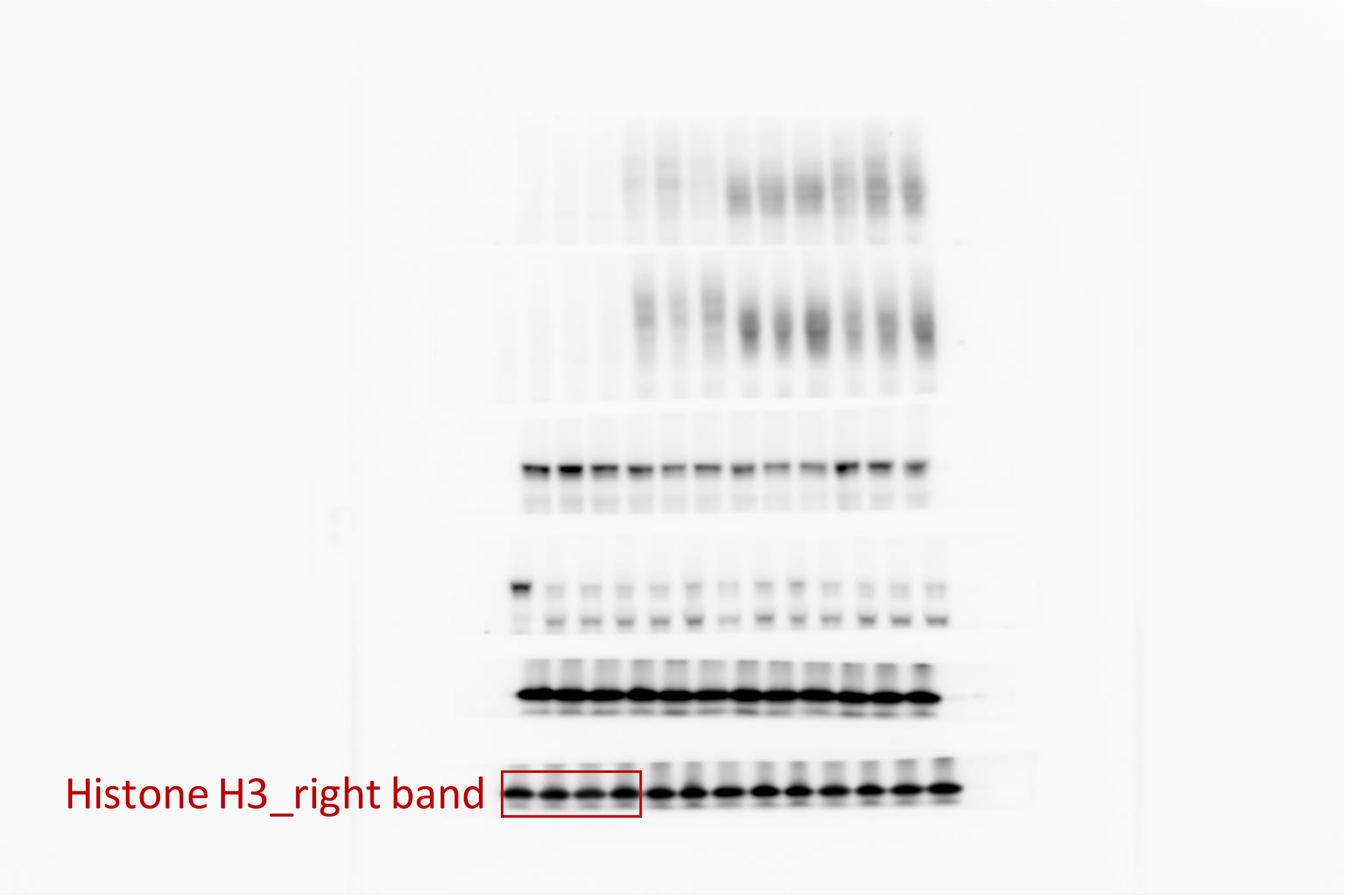

Full_unedited_gel_for_Figure_S6D_20190501_1353_8.tif
